# Local application of otoprotective compounds other than sodium thiosulfate to prevent cisplatin-induced hearing loss: a systematic review

**DOI:** 10.1080/10717544.2026.2665892

**Published:** 2026-05-01

**Authors:** Amirhossein Masroor, Nienke Streefkerk, Martine Van Grotel, James I. Geller, Marc Ansari, Eric Bouffet, Archie Bleyer, Brice Fresneau, Michael Sullivan, Kristin Knight, Per Kogner, Rudolf Maibach, Allison F. O'neill, Vassilios Papadakis, Kaukab M. Rajput, Penelope R. Brock, Gareth J. Veal, Alexander E. Hoetink, Alwin D. R. Huitema, Marry M. Van Den Heuvel- Eibrink

**Affiliations:** a Princess Máxima Center for Pediatric Oncology, Utrecht, the Netherlands; b Division of Hematology/Oncology, Department of Pediatrics, Peckham Center for Cancer and Blood Disorders, Rady Children's Hospital, San Diego, CA, United States of America; c University of California San Diego, La Jolla, CA, United States of America; d Department of Pediatrics, Gynecology and Obstetrics, Cansearch Research Platform for Pediatric Oncology and Hematology, Faculty of Medicine, University of Geneva, Geneva, Switzerland; e Division of Pediatric Oncology and Hematology, Department of Women, Child and Adolescent, University Geneva Hospitals, Geneva, Switzerland; f Division of Pediatric Neuro-Oncology, The Hospital for Sick Children, University of Toronto, Toronto, Ontario, Canada; g Department of Radiation Oncology, Knight Cancer Institute, Oregon Health and Science University, Portland, OR, United States of America; h Department of Children and Adolescents Oncology, Gustave Roussy, University Paris Saclay and Radiation Epidemiology Team, CESP, Inserm U1018, Villejuif, France; i Children's Cancer Centre and Department of Pediatric Oncology, Royal Children's Hospital, Melbourne, Victoria, Australia; j Department of Pediatric Audiology, Oregon Health and Science University, Portland, OR, United States of America; k Department of Women's and Children's Health, Pediatric Oncology, Karolinska University Hospital and Childhood Cancer Research Unit, Karolinska Institutet, Stockholm, Sweden; l ETOP IBCSG Partners Foundation, Bern, Switzerland; m Dana-Farber/Boston Children's Cancer and Blood Disorders Center, Boston, MA, United States of America; n Department of Pediatric Hematology-Oncology (TAO), Agia Sofia Children's Hospital, Athens, Greece; o Department of Pediatric Audiology, Great Ormond Street Hospital for Children NHS Foundation Trust, London, United Kingdom; p Newcastle University Centre for Cancer, Translational and Clinical Research Institute, Newcastle University, Newcastle Upon Tyne, United Kingdom; q Department of Otorhinolaryngology and Head & Neck Surgery, University Medical Center Utrecht, Utrecht University, Utrecht, the Netherlands; r Department of Clinical Pharmacy, University Medical Center Utrecht, Utrecht University, Utrecht, the Netherlands; s Department of Pharmacy and Pharmacology, Netherlands Cancer Institute, Antoni van Leeuwenhoek Hospital, Amsterdam, the Netherlands; t Division of Child Health, Wilhelmina Children's Hospital, University Medical Center Utrecht, Utrecht University, Utrecht, the Netherlands

**Keywords:** Childhood cancer, cisplatin, ototoxicity, hearing loss, local intratympanic otoprotection

## Abstract

Cisplatin-induced hearing loss (CIHL) in pediatric cancer patients is an irreversible and highly prevalent adverse effect with a devastating impact on quality of life. Sodium thiosulfate (STS) has recently been approved for systemic administration as an otoprotective agent in children. However, implementation of systemic STS has its challenges, and there is currently limited evidence to support local STS for children. This review investigates the potential value of locally administered otoprotective agents other than STS with a focus on future pediatric implementation. We conducted a systematic review on the efficacy and safety of locally applied non-STS otoprotective agents in *in vivo* settings. This included a summary of investigated drug delivery methods and administration routes. We identified 70 preclinical and eight clinical studies. Agents were categorized based on their biological mechanisms: anti-inflammatory, chemical deactivators, calcium blockers, biologicals, and miscellaneous mechanisms. Preclinical studies investigated 45 different agents. Dexamethasone and *N*-acetylcysteine were identified as efficacious agents recurrently and progressed to clinical trials. Dexamethasone was investigated in three randomized clinical trials (RCTs) and three non-randomized clinical studies and showed statistically significant but not clinically relevant benefit in two trials. *N*-acetylcysteine was investigated in two clinical trials and one RCT and was minimally effective in the RCT and in one clinical study. Our review did not identify available studies of local alternative otoprotective agents that could reliably replace systemic STS in terms of safety and efficacy for pediatric patients. Further research on the optimal dosage, delivery method, and timing of otoprotective agents is needed.

## Background

Cisplatin has played an important role in the increase of survival rates of pediatric solid and brain tumor patients over the last decades. However, this success comes at a cost, with cisplatin-induced hearing loss (CIHL) reported in 20%–70% of exposed children (Knight et al. 2007; Al-Khatib et al. 2010; Einar-Jon et al. 2011; Landier et al. 2014; Clemens et al. 2016).

Cisplatin can enter and accumulate in cochlear outer hair cells through multiple transporter pathways, including the high-affinity copper transporter 1, and the organic cation transporter 2 (Sheth et al. 2017; Yu et al. 2025). Once inside the cell, cisplatin damages outer hair cells (OHCs) in the cochlea by cross-linking DNA, disrupting transcription and replication, and causing excessive formation of reactive oxygen species (ROS). Elevated ROS overwhelm endogenous antioxidant pathways and lead to oxidative damage, lipid peroxidation, and induction of apoptosis. In addition to the induction of apoptosis, ROS also act as mediators of inflammation by activating transcription factors such as signal transducer and activator of transcription-1 (STAT1), and ion channels such as transient receptor vanilloid 1 (TRPV1). As a result, oxidative stress promotes inflammatory processes, while inflammatory signaling further increases ROS production (Sheth et al. 2017; Yu et al. 2025).

OHCs facilitate the transmission of sound to the acoustic nerve towards the auditory cortex (Peterson et al. 2025). This damage to the OHCs is usually irreversible, and hearing loss can worsen after the end of treatment (Clemens et al. 2017; Breglio et al. 2017). CIHL is known to greatly impact children's speech-language development, psychological and neuro-cognitive development, development of social skills and, therefore, relational interactions, and consequently compromises overall quality of life (Bess et al. 1998; Knight et al. 2017; Wells et al. 2018).

Numerous studies have been carried out focusing on the prevention of CIHL, and the potential otoprotective effects of many drugs have been studied (Hazlitt et al. 2018; Yu et al. 2020). Systemic application of sodium thiosulfate (STS), the most effective otoprotective agent, has been recently approved by several regulatory agencies for use in pediatric cancer patients with non-metastatic disease that require cisplatin-based treatment, based on two successful pediatric clinical trials (Freyer et al. 2017; Brock et al. 2018). However, implementation of STS in clinical settings faces economic and logistical challenges and, due to concerns for theoretical (yet unproven) tumor protection, is currently restricted to non-disseminated disease (Brock et al. 2023; Meijer et al. 2024; Ma et al. 2025).

Therefore, other administration routes for otoprotective agents have been proposed and extensively investigated in preclinical and clinical adult studies (Harned et al. 2008; Chirtes and Albu 2014; Callejo et al. 2015; Yu et al. 2020; Streefkerk et al. 2025). We recently showed that despite a strong preclinical rationale for local STS, this was not translated to tremendous efficacy in adult clinical trials (Streefkerk et al. 2025). In pediatrics, only one multicenter study (*n* = 11 patients) reporting the administration of intratympanic otoprotectives (dexamethasone) is available, which suggests the safety and feasibility of this procedure (Freyer et al. 2025).

Over the last decades, in addition to STS, several other compounds have been studied for local otoprotective use, but a comprehensive overview is lacking. Therefore, we reviewed all available preclinical and clinical studies for the safety and efficacy of local application of otoprotective agents other than STS.

## Methods

### Search strategy and selection of papers

**Figure 1.**
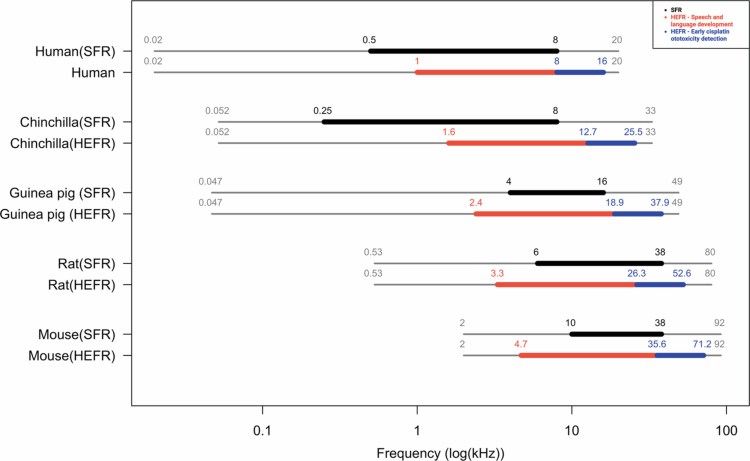
SFR and HEFR in humans and different lab species. (SFR): frequencies detectable at ~15 decibels (dB) sound pressure level (SPL) in behavioral audiometry; 2) Human equivalent frequency range (HEFR): the frequency range relevant to human speech perception (0.5–8 kHz) and early CIHL detection (8–16 kHz). SFR helps interpret auditory results and the biological relevance of the otoprotective drug, while HEFR helps determine the impact and translate the auditory results to clinical settings. SFR of chinchillas (Heffner and Heffner 1991), guinea pigs (Heffner et al. 1971), rats (Kelly and Masterton 1977; Heffner et al. 1994; Backoff and Caspary 1994), and mice (Henry et al. 1992; Zheng et al. 1999; Koay et al. 2002) have been extracted from studies reporting physiological behavioral auditory measurements. SFR of Human has been extracted from the Munson curve (Fletcher and Munson 1933; Suzuki and Takeshima 2004). HEFR of lab animals has been calculated for the frequency range of (0.5–16 kHz) in humans. SFR and HEFR are shown on grey lines representing the audible frequency range of each species.

A comprehensive literature search, using the PubMed database, was performed. The inclusion criteria were: 1) efficacy and/or safety data for locally administered otoprotective drugs in cisplatin-treated subjects, 2) studies performed in an *in vivo* setting, either clinical or preclinical, and 3) original research. The search included all reports published prior to May 2025, other than single case reports, commentaries, reviews, or articles written in languages other than English, as well as studies limited to STS. Two independent reviewers performed title/abstract screening and full-text screening, and disagreements were resolved by consulting a third reviewer. A cross-reference check was performed. Search terms (Supplementary file 1. Table 1) and the screening process, including a PRISMA flowchart (Supplementary file 1. Figure 1) and completed PRISMA checklist (Supplementary file 2), are provided as supplementary material.

From all selected studies, data were summarized on included subjects, study design, otoprotective drug, drug delivery method and vehicle, administration mode, and dose of platinum treatment, time to follow up, audiological endpoints (audiological evaluation, and/or outer hair cell (OHC) count), as well as reported efficacy and/or safety.

### Interpretation of audiological outcomes

#### Preclinical studies

Accurate interpretation of auditory results in preclinical animal settings is necessary for the translation, implementation, and testing of local otoprotective drugs in a clinical trial setting (Domarecka and Szczepek 2023). While mammalian auditory systems are broadly similar, differences exist in cochlear spiral length and audible frequency ranges (West 1985). Nonetheless, the correlation between hair cell location and frequency sensitivity follows a conserved pattern across defined mammalian species. The Greenwood's (cochlear) frequency-position function describes this correlation by mapping cochlear position to frequency on a normalized cochlear length (Greenwood 1974, 1990, 1996). It is used to determine equivalent cochlear locations and the respective frequencies across mammalian species, including humans, with similar cochlear maps, facilitating inter-species comparison (Greenwood 1990). To estimate the clinical relevance of preclinical audiological outcomes to humans, two key frequency ranges were used identified: 1) Sensitive frequency range (SFR): frequencies detectable at ~15 decibels (dB) sound pressure level (SPL) in behavioral audiometry; 2) Human equivalent frequency range (HEFR): the frequency range relevant to human speech perception (0.5–8 kHz) and early CIHL detection (8–16 kHz) (Brooks and Knight 2018; Basheer et al. 2025), translated to animal models via the Greenwood function (Figure 1).

For the purpose of this review, the criterion for otoprotective efficacy of investigational drugs in preclinical studies, based on guidelines and expert consensus, as a threshold difference of ≥20 dB at least in two adjacent tested frequencies between study arms within the SFR and HEFR was used (Audiology 2009; Domarecka and Szczepek 2023).

#### Clinical studies

For the purpose of this review, we defined an otoprotective threshold in clinical studies according to both clinical relevance and developmental impact (Knight et al. 2017; Meijer et al. 2021). In adult ototoxicity monitoring, a ≥20 dB decrease at a single frequency, a ≥10 dB decrease at two consecutive frequencies, or a ≥5 dB decrease at three consecutive frequencies, given prior normal hearing, is considered a clinically significant drop in hearing (American Speech-Language-Hearing Association 1997). Consequently, we considered an impactful otoprotective threshold as a threshold difference between the study arms in clinical studies, which is ≥20 dB in electrophysiological/behavioral threshold measurements at frequencies ≥4 kHz, as relevant, as we are trying to find evidence for use in pediatric settings where speech and language development are important (Clemens et al. 2019). For this effort, we categorized identified studies into five categories, based on functional characteristics of the investigated compounds: 1) anti-inflammatory compounds, 2) chemical deactivators, 3) non-inflammatory biologicals, 4) calcium blockers, and 5) miscellaneous compounds.

## Results

The search yielded 1049 publications, of which 76 papers were included after title/abstract and full-text screening (Supplementary file 1: Figure 1). Cross-referencing identified two additional publications. In total, 70 preclinical studies and eight clinical trials investigating 45 different compounds were identified (Tables 1 and 2).

**Table 1.** Summary of preclinical studies on the efficacy and safety of locally administered otoprotective agents.

								Design (no.)					Results							
Subjects (no.)								Ear vs. ear	Subject vs. subject				Efficacy		Safety		≥20 dB			
WR	GP	M	FR	CH	SD	R	No. of studies			Drugs enlisted (no.)	Cumulative drug dose(min–max)	TCCD(min–max)	+	−	+	−	+	−	Remarks	Ref
**Anti-inflammatories**																				
12	167	45	0	0	0	11	17^e^	6	11	Dexamethasone	0.1–40 mg/mL	8–20 mg/kg IP	15	2	6	NA	3	9	Most efficacy from near-infrared (NIR) responsive nanocomposites and hydrogel containing microcarriers/Small tympanic perforations reported	Daldal et al. (2007); Hill et al. (2008); Parham (2011); Murphy and Daniel (2011); Paksoy et al. (2011); Shafik et al. (2013); Hughes et al. (2014); Sun et al. (2015); Fernandez et al. (2016); Ozel et al. (2016); Martin-Saldana et al. (2017); Martin-Saldana et al. (2018); Chen et al. (2019); Simsek et al. (2019); Tas et al. (2021); Dindelegan et al. (2024); Mustafa et al. (2025)
50	3	22	0	0	0	0	5	2	3	Methylprednisolone	4–15 mg/mL	4–15 mg/kg IP	3	2	NA	NA	0	3		Saliba et al. (2010); Ozel et al. (2016); Martin-Saldana et al. (2016); Ramaswamy et al. (2017); Tas et al. (2021)
0	9	0	0	0	0	0	1	0	0	Fluticasone propionate	Nanoparticle	12 mg/kg IP	1	0	NA	NA	NA	NA		Pierstorff et al. (2019)
16	0	0	0	0	0	0	1	0	1	Vitamin E	4 g/kg	20 mg/kg IP	1	0	1	NA	1	0	No side effects reported	Paksoy et al. (2011)
24	0	0	0	0	0	0	1	0	1	Resveratrol	20 mg/mL	15 mg/kg IP	1	0	NA	NA	NA	NA		Simsek et al. (2019)
0	0	10	0	0	0	0	1	1	0	Ergothioneine	500 mM	2 mg/mL IT	1	0	NA	NA	0	1	Significant protection only at 32 kHz	Zhao et al. (2025)
12	0	0	0	0	0	0	1	1	0	*α*-tocopheryl succinate	Nano particle	10 mg/kg IP	1	0	NA	NA	NA	NA		Martin-Saldana et al. (2017)
0	21	0	0	0	0	0	1	0	1	Curcumin/tanshinone	CUR: 1.5 μg/kg, TSA: 3 μg/kg	12 mg/kg IP	1	0	NA	NA	1	0	Magnetic/acoustic dual-controlled drug release	Yi et al. (2024)
**Chemical deactivators**																				
0	148	0	0	0	0	0	5	2	3	*N*-acetylcysteine	1%–20%	10–24 mg/kg IP	3	2	NA	NA	1	2	4% and 20% NAC: inflammation, diffuse osteosis	Choe et al. (2004); Saliba et al. (2010); Nader et al. (2010); Mohan et al. (2014); Chen et al. (2022)
0	12	0	0	13	0	0	2	1	1	D-Methionine	25–40 mg/mL	0.66–15 mg/mL IP	2	0	NA	NA	1	NA		Korver et al. (2002); Wimmer et al. (2004)
0	33	0	0	0	0	0	2	1	1	Lactate in Ringer's	NA	3–10 mg/kg IP	2	0	1	NA	NA	NA	Better protection compared to 2% and 20% Nac	Choe et al. (2004); Nader et al. (2010)
0	40	0	0	0	0	0	2	0	2	Thermal treatment	Irrigation-cool ear bar	12 mg/kg IP	2	0	NA	NA	2	0	Low temperature of the ear canal is protective	Spankovich et al. (2016); Stanford et al. (2021)
0	0	0	23	0	0	0	1	0	1	L-Methionine	25 mg/mL	15 mg/kg IP	1	0	NA	NA	0	1		Li et al. (2001)
0	20	0	0	0	0	0	1	0	1	Thiourea	27 mg/mL	8 mg/kg IP	1	0	NA	1	0	1	Inflammation of the middle ear	Ekborn et al. (2003)
0	42	0	0	0	0	0	1	0	1	Caspase-3, 8, 9 inhibitor	72–85 mmol/l	10 mg/kg IP	1	0	NA	NA	1	0		Wang et al. (2004)
0	0	15	0	0	0	0	1	0	1	Copper sulfate	0.025 mg/kg	20 mg/kg IP	1	0	NA	NA	0	1		More et al. (2010)
0	20	0	0	0	0	0	1	1	0	Erdosteine	1.125–4.5 mg/cc	24 mg/kg IP	0	1	0	1	0	1	Diffuse inflammatory reaction and osteosis	Saliba and El Fata (2012)
7	0	0	0	0	0	0	1	1	0	Dimethyl sulphoxide	0.5% DMSO	10 mg/kg IP	0	1	NA	NA	0	1		Roldan-Fidalgo et al. (2014)
0	0	0	0	0	0	20	1^e^	1	1	PH manipulation	Acidic-basic PBS (1 µL)	13 mg/kg IP	1	0	NA	NA	0	1	Basic PH is protective	Tanaka et al. (2004)
**Calcium blockers**																				
0	20	61	0	0	0	0	3^e^	1	3	Diltiazem	2 mg/kg	8–14 mg/kg IP	3	0	NA	NA	1	1	Chitosan hydrogel increased half-life of diltiazem	Naples and Parham (2016); Naples et al. (2018); Naples et al. (2020)
0	0	0	0	0	56	0	1	0	1	L-arginine, calmodulin-dependent kinase II inhibitor	L-Arg 50 mg/kg + KN93 10 mg/kg	13.8 mg/kg IP	1	0	NA	NA	1	0		Zhou et al. (2025)
0	16	0	0	0	0	0	1	0	1	Memantine	0.2 mL of 2 mg in hydrochloride sol.	12 mg/kg IP	0	1	NA	NA	0	1		Guven et al. (2025)
**Biologicals**																				
NS	0	0	0	0	0	0	5	0	5	siRNA^a^	0.3–0.9 µg, 1 µM	11 mg/kg IP	5	0	NA	NA	1	3	Different targets in each study	Mukherjea et al. (2010); Kaur et al. (2011); Ghosh et al. (2018); Sheehan et al. (2018); Al Aameri et al. (2023)
0	14	0	0	31	0	0	3	3	0	XIAP^b^ (*n* = 46)	3–10 µL	12–32 mg/kg IP	3	0	NA	NA	2	1	Adeno-associated viral vector is a safe vehicle	Cooper et al. (2006); Chan et al. (2007); Jie et al. (2015)
28	21	0	0	0	0	0	3	1	2	BDNF^c^	7.5 µg/mL, 0.5 µg, 5 μg/μL	12–15 mg/kg IP	1	2	NA	NA	0	1	Cochleostomy is not a safe drug delivery method	Wimmer et al. (2004); Meen et al. (2010); Pisani et al. (2025)
0	7	0	0	0	0	0	1	1	0	GDNF^d^	1 mg/mL, 50 ng/mL	15 mg/kg IP	1	0	NA	NA	NA	NA		Kuang et al. (1999)
0	0	20	0	0	0	0	1	0	1	Umbilical cord-derived mesenchymal stromal cells	1.2 μg/μL	20 mg/kg IP	1	0	NA	NA	0	1		Tsai et al. (2021)
0	0	0	0	0	0	8	1	0	1	Platelet-rich plasma	0.1–0.3 mL	20 mg/kg IP	1	0	NA	NA	NA	NA		Yurtsever et al. (2020)
**Miscellaneous category**																				
11	0	28	0	0	0	0	2	0	2	Kenpaullone, AZD5438 (*n* = 39)	310 μM, 5 μL	13–30 mg/kg IP	2	0	2	0	1	1	No side effects reported for CDK2 inhibitors	Hazlitt et al. (2018); Teitz et al. (2018)
0	0	0	0	0	12	0	1	1	0	Tauroursodeoxycholic acid	0.5 mg/mL	12 mg/kg IP	1	0	1	0	1	0	No side effects reported	Wen et al. (2021)
0	0	0	0	18	0	0	1	0	1	Adenosine receptor agonist	1 mM	2 µL IT	1	0	NA	NA	1	0	Used in siRNA study vehicles	Whitworth et al. (2004)
16	0	0	0	0	0	0	1	0	1	Melatonin	0.5 mg/mL	12 mg/kg IP	1	0	NA	NA	1	0		Demir et al. (2015)
0	0	0	0	0	0	11	1	0	1	Vitamin C	100 mg/mL	16 mg/kg IP	1	0	1	0	NA	NA	No side effects reported	Celebi et al. (2013)
0	12	15	0	0	0	0	2	0	2	α-Lipoic acid	25 mg/m, 1 mM PDA@microcarrier	12 mg/kg IP	1	1	NA	NA	1	NA	PDA nanohydrogel significantly increased the therapeutic efficacy of α-lipoic acid	Ozkul et al. (2014); Chen et al. (2025)
0	0	12	0	0	0	0	1	1	0	Astaxanthine	400 µmol/L	12 mg/kg IP	1	0	NA	NA	1	0		Nan et al. (2022)
8	0	0	0	0	0	0	1	1	0	Lutein	1 mg/mL	10 mg/kg IP	0	1	1	0	0	1		Roldan-Fidalgo et al. (2016)
12	0	0	0	0	0	0	1	0	1	Folic Acid	6 mL	20 mg/kg IP	1	0	NA	NA	1	0		Tanyeli et al. (2019)
12	0	0	0	0	0	0	1	0	1	Oxytocin	0.4–1.2 mL	20 mg/kg IP	1	0	NA	NA	NA	NA		Bekmez Bilmez et al. (2016)
0	0	0	0	0	8	0	1	1	0	KR-22332	10 mM	14 mg/kg IP	1	0	NA	NA	NA	NA		Shin et al. (2013)
0	0	0	0	0	8	0	1	1	0	Epicatechin	10 mM	14 mg/kg IP	1	0	NA	NA	NA	NA		Lee et al. (2010)

TCCD: total cumulative cisplatin dosage, WR: Wistar rats, GP: guinea pigs, M: mice, FR: Fisher 334 rats, CH: chinchilla, SD: Sprague–Dawley rats, R: rats, IT: intratympanic, IP: intraperitoneal.

Detailed information about studies can be found in supplementary file 1. Table 2A–E.

^a^ siRNA list: *TRPV1, NOX3, STAT1, CB2R, R-PIA, SB225002, CXCR2.*

^b^ Gene encoding X-linked inhibitor of apoptosis protein.

^c^ BDNF: brain-derived nerve growth factor.

^d^ GDNF: glial cell line-derived neurotrophic factor.

^e^ One or more studies had both study designs.

**Table 2.** Summary of clinical studies on the efficacy and safety of locally administered otoprotective agents.

Study	No. (age)	Design	Cisplatin cumulative dose	Drug	Concentration	Results		Conclusion		Remarks
						Cases	Controls	Efficacy	Safety	
Marshak et al. (2014) (RCT)	15 (38–80)	Ear vs. ear	517 ± 184 mg 3–11 cycles	Dex	0.7–1 mL of 10 mg/mL, before each Cis treatment	PTA (dB HL): 0.5–3 kHz: 18.3 ± 9, 4-8 kHz: 41.1 ± 19.4 DPOAE (dB SPL): 1-3 kHz: 8.9 ± 4.7, 4-8 kHz: 6.7 ± 5.9	PTA (dB HL): 0.5-3 kHz: 19 ± 8.6, 4-8 kHz: 43.8 ± 18.7 DPOAE (dB SPL): 1-3 kHz: 9.1 ± 4.6, 4-8 kHz: 5.8 ± 4.8	+ –	+	Most patients had high-tone hearing loss before treatment. Slight pain during injection and short, mild vertigo were reported. Loss of injected solution through the Eustachian tube was unavoidable.
Sarafraz et al. (2018) (RCT)	57 (6–60)	Patient vs. patient	NA 1–3 cycles	Dex	0.4–0.8 mL of 24 mg/mL, during hydration	3 Cis injections (Dex): 0.25 kHz: 21.4 ± 3.1 dB, 0.5 kHz: 22.4 ± 2.9 dB, 1 kHz: 22.3 ± 2.3 dB, 2 kHz: 23.6 ± 2.5 dB, 4 kHz: 25 ± 3.5 dB, 8 kHz: 34.7 ± 5.4 dB		–	–	20 patients presented with tinnitus
Moreno and Belinchon (2022) (RCT)	23 (44.2–74.8)	Ear vs. ear	444.87 ± 235.2 mg	Dex	1 mL of 8 mg/mL/24 h microwick system during Cis treatment	PTA (dB HL): 125 Hz: 24.7 ± 12.9 dB, 250 Hz: 15 ± 11.3 dB, 500 Hz: 16.2 ± 7 dB, 1 kHz: 15.6 ± 8.1 dB, 2 kHz: 21.2 ± 12.8 dB, 3 kHz: 34.4 ± 20.4 dB, 4 kHz: 45.9 ± 35.6 dB, 6 kHz: 62.9 ± 18.7 dB, 8 kHz: 69.4 ± 19 dB	PTA (dB HL): 125 Hz: 17.4 ± 8.5 dB, 250 Hz: 11.5 ± 7.7 dB, 500 Hz: 11.5 ± 4.6 dB, 1 kHz: 10.6 ± 7.9 dB, 2 kHz: 15.9 ± 7.6 dB, 3 kHz: 27.6 ± 17.1 dB, 4 kHz: 35.6 ± 16.4 dB, 6 kHz: 46.2 ± 20.2 dB, 8 kHz: 61.8 ± 19.8 dB	+	+	Although hearing threshold differences were statistically significant, they were not clinically significant according to the ASHA hearing loss criteria. Microwick system is safe and easy to use at home by patients. 2 cases of otorrhea and 8 cases of permanent perforation were reported
Nasr et al. (2018)	20 (40–65)	Ear vs. ear	546.3 ± 111.58 mg 5.5 ± 1 cycles	Dex	0.3–0.5 mL of 4 mg/mL, after detection of HL	PTA (dB HL): 500 Hz: 13.95 ± 3.6, 1 kHz: 16.2 ± 6.1, 2 kHz: 23.25 ± 8.5, 3 kHz: 22.85 ± 8.6, 4 kHz: 40 ± 11.3, 6 kHz: 39.7 ± 11.9, 8 kHz: 43.15 ± 15.6	PTA (dB HL): 500 Hz: 15.8 ± 4.3, 1 kHz: 15.25 ± 5.7, 2 kHz: 22.58 ± 4.8, 3 kHz: 20.45 ± 7.1, 4 kHz: 40.85 ± 7.7, 6 kHz: 52.6 ± 7.4, 8 kHz: 55.7 ± 13.3	+	–	Minimal otoprotection at high frequencies when injected shortly after observing hearing loss
Gupta et al. (2023)	100 (Mean 58.2 ± 6.3)	Ear vs. ear	300 mg, 6 weeks	Dex	0.5–0.7 mL of 4 mg/mL, 1 h before Cis treatment	PTA (dB HL): 250 Hz: 27.3 ± 2.5, 500 Hz: 27.95 ± 3.2, 1 kHz: 19.9 ± 1.6, 2 kHz: 21.35 ± 2.2, 4 kHz: 35.45 ± 4.6, 6 kHz: 42.1 ± 3.2, 8 kHz: 41.65 ± 5.6, DPOAE (dB SPL): 998 Hz: 7.4 ± 0.64, 1481 Hz: 7.4 ± 0.85, 2222 Hz: 6.9 ± 0.56, 2963 Hz: 8.9 ± 1.13, 4444 Hz: 7.9 ± 0.44, 5714 Hz: 5.3 ± 0.94, 8000 Hz: 5.5 ± 1.36	PTA (dB HL): 250 Hz: 27.15 ± 2.8, 500 Hz: 28.9 ± 3.5, 1 kHz: 20.8 ± 2.6, 2 kHz: 20.25 ± 2.7, 4 kHz: 36.45 ± 4.5, 6 kHz: 45.75 ± 3.3, 8 kHz: 47.55 ± 3.5, DPOAE (dB SPL): 998 Hz: 7.4 ± 1.30, 1481 Hz: 7.4 ± 0.75, 2222 Hz: 7.8 ± 0.99, 2963 Hz: 7.2 ± 0.83, 4444 Hz: 6.1 ± 0.90, 5714 Hz: 3.4 ± 0.74, 8000 Hz: 3.1 ± 0.93	+ –	+	Hearing threshold shift seen at lower frequencies is related to the oropharynx and nasopharynx sites of HNC. Dex is less effective at high frequencies. Tinnitus due to the injection resolved shortly after.
Freyer et al. (2025) (RCT)	11 (0.5–14)	Ear vs. ear	1-3 cycles of Cis (117 to 393 mg/m^2^)	Dex	1–3 doses of 0.2 mL of OTO-104 (12 mg/m^2^) 7–64 h before Cis	After 1 st Cis cycle 2 kHz: 0.5 ± 17.1 dB HL 4 kHz: 9.5 ± 22.9 dB HL 6 kHz: 17.2 ± 25.6 dB HL 8 kHz: 20.5 ± 23.5 dB HL After 2nd Cis cycle 2 kHz: -2.9 ± 4.9 dB HL 4 kHz: 17.1 ± 22.5 dB HL 6 kHz: 31.0 ± 33.1 dB HL 8 kHz: 48.3 ± 25.6 dB HL	After 1 st Cis cycle 2 kHz: 4.1 ± 25.3 dB HL 4 kHz: 9.5 ± 28.6 dB HL 6 kHz: 17.8 ± 34.5 dB HL 8 kHz: 22.5 ± 32.1 dB HL After 2nd Cis cycle 2 kHz: -2.1 ± 4.9 dB HL 4 kHz: 14.3 ± 17.7 dB HL 6 kHz: 33.0 ± 30.5 dB HL 8 kHz: 45.8 ± 17.4 dB HL	–	+	CIHL appeared in treated and untreated ears similarly, and the trial was terminated.
Sarafraz et al. (2018) (RCT)	57 (6–60)	Patient vs. patient	NA 1–3 cycles	*N*-acetylcysteine	0.4–0.8 mL 10% solution diluted with R-lactate, during hydration	3 Cis injections (NAC): 0.25 kHz: 20 ± 2.2 dB, 0.5 kHz: 20.8 ± 2 dB, 1 kHz: 20.8 ± 2.1 dB, 2 kHz: 22.2 ± 2.7 dB, 4 kHz: 22.7 ± 2.8 dB, 8 kHz: 23.8 ± 2.8 dB		+	+	Nac-treated patients had close to baseline protection 6 months after treatment.
Riga et al. (2013)	20 (16–77)	Ear vs. ear	120–720 mg/m^2^	*N*-acetylcysteine	0.4–0.8 mL of 10% solution diluted with R-lactate, during hydration	PTA (dB HL): 0.25 kHz: 1 ± 7.4 dB HL, 0.5 kHz: 1.3 ± 7.2 dB HL, 1 kHz: 2.3 ± 6 dB HL, 2k Hz: 0.8 ± 8.3 dB HL, 4 kHz: 0.8 ± 11.6 dB HL, 8 kHz: 0.8 ± 11.3 dB HL,	PTA (dB HL): 0.25 kHz: 1.5 ± 8.3 dB HL, 0.5 kHz: 0.3 ± 5.6 dB HL, 1 kHz: 4 ± 6.6 dB HL, 2 kHz: 4.3 ± 11.4 dB HL, 4 kHz: 1.3 ± 9.4 dB HL, 8 kHz: 7.8 ± 11.8 dB HL	+	+	Significant otoprotection at 8 kHz. Acute pain shortly after injection, lasting a few minutes, was reported.
Yoo et al. (2014) (pilot)	11 (29–68)	Ear vs. ear	100 mg/m^2^ 2–6 dose	L-*N*-acetylcysteine	2–3 mL of 2% solution, 30–60 min before Cis treatment	PTA (dB HL): 0.25 kHz: 9.5 dB, 0.5 kHz: 5.5 dB, 1 kHz: 3.2 dB, 2 kHz: 4.5 dB, 3 kHz: 15.0 dB, 4 kHz: 19.1 dB, 6 kHz: 30.5 dB, 8 kHz: 36.4 dB, 9 kHz: 40.0 dB, 10 kHz: 37.7 dB, 11.2 kHz: 35.0 dB, 12.5 kHz: 21.8 dB, 14 kHz: 13.2 dB, 16 kHz: 9.1 dB, 18 kHz: 8.6 dB, 20 kHz: 5.5 dB	PTA (dB HL): 0.25 kHz: 6.4 dB, 0.5 kHz: 5.5 dB, 1 kHz: 2.7 dB, 2 kHz: 3.6 dB, 3 kHz: 20.5 dB, 4 kHz: 25 dB, 6 kHz: 32.7 dB, 8 kHz: 41.4 dB, 9 kHz: 45.5 dB, 10 kHz: 44.5 dB, 11.2 kHz: 40.0 dB, 12.5 kHz: 27.7 dB, 14 kHz: 16.8 dB, 16 kHz: 10.0 dB, 18 kHz: 9.1 dB, 20 kHz: 7.7 dB	–	+	Myringotomy and 2% Nac were safe and well-tolerated. Only two patients had a 10 dB difference between experimental and control ears. Loss of aqueous solution through the Eustachian tube.

Dex: dexamethasone, NAC: N-acetylcysteine, Cis: cisplatin, PTA: pure tone audiometry, dB: decibel, CIHL: cisplatin-induced hearing loss, HL: hearing level, SPL: sound pressure level.

### Preclinical studies on the efficacy and/or safety of local (non-STS) otoprotective agents

#### Anti-inflammatory compounds

The efficacy and safety of nine transtympanically administered anti-inflammatory agents to prevent CIHL were investigated in 22 preclinical studies. Study subjects included guinea pigs (9 reports), Wistar rats (8 reports), and mice (5 reports). Studies employed either a subject-versus-subject or ear-versus-ear design. Cisplatin dosage and administration protocols varied per study, as well as the timing, route, and dosage of the anti-inflammatory agents. Audiological outcomes were mainly assessed via auditory brainstem response (ABR) or distortion product otoacoustic emissions (DPOAE). Three studies used auditory steady-state response (ASSR) and two studies reported OHC counts alongside audiologic testing (Supplementary file 1. Table 2A).

Dexamethasone as a local otoprotective agent was the most frequently studied agent (17 reports) (Daldal et al. 2007; Hill et al. 2008; Parham 2011; Murphy and Daniel 2011; Shafik et al. 2013; Hughes et al. 2014; Sun et al. 2015; Fernandez et al. 2016; Martin-Saldana et al. 2018; Chen et al. 2019; Dindelegan et al. 2024; Mustafa et al. 2025); two studies compared dexamethasone with methylprednisolone (Ozel et al. 2016; Tas et al. 2021) and three compared dexamethasone with either vitamin E (Paksoy et al. 2011), *α*-tocopherol succinate (Martin-Saldana et al. 2017), or resveratrol (Simsek et al. 2019). Fifteen of these preclinical studies reported efficacy of local dexamethasone, of which three studies met the review-defined otoprotection criteria with ≥20 dB threshold difference between study arms in at least two adjacent measured frequencies (Paksoy et al. 2011; Dindelegan et al. 2024; Mustafa et al. 2025). The efficacy of methylprednisolone was assessed in five studies, three of which reported efficacy, but none reached the review-defined otoprotection criteria (Saliba et al. 2010; Ozel et al. 2016; Martin-Saldana et al. 2016; Ramaswamy et al. 2017; Tas et al. 2021). Fluticasone propionate (Pierstorff et al. 2019), vitamin E (Paksoy et al. 2011), and resveratrol (Simsek et al. 2019) each reported significant effects in single studies, with only vitamin E having ≥20 dB threshold difference between study arms in at least two adjacent measured frequencies. In contrast, *α*-tocopherol succinate showed partial protection at low doses (Martin-Saldana et al. 2017).

**Figure 2.**
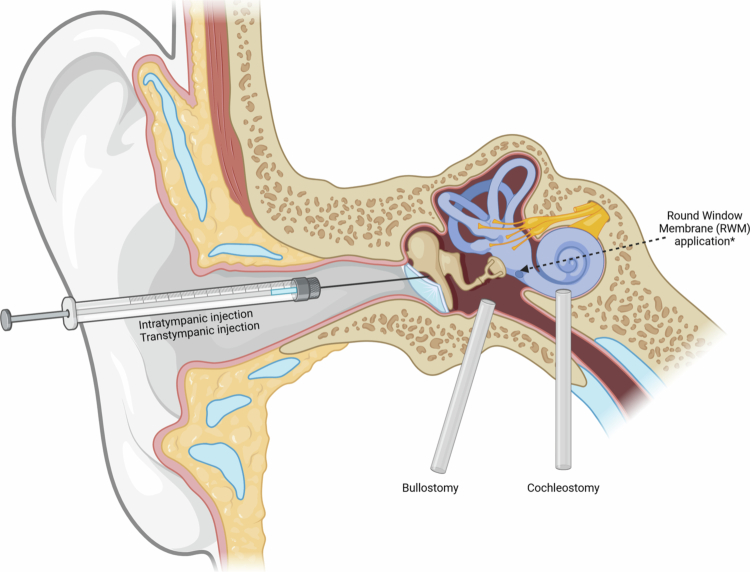
Localized administration routes.

In the anti-inflammatory studies, the drug administration route differed per study and per drug. Otoprotective agents were administered through bullostomy in three studies, round window membrane (RWM) application in three, and intratympanic injection in sixteen studies (Figure 2). Intratympanic injection involves transtympanic delivery of a compound into the middle ear cavity, relying on passive diffusion across the RWM (Hao and Li 2019). RWM application refers to the targeted placement of the therapeutic compound onto the RWM niche to promote controlled diffusion into the inner ear, often achieved through bullostomy in experimental animal models (Borkholder et al. 2014). Bullostomy creates a surgical opening of the bulla to allow visualization of the RWM and controlled middle ear drug delivery (Murillo-Cuesta et al. 2017).

In eleven studies, the influence of using novel drug delivery systems was investigated, as well as the efficacy of the otoprotectant. In seven dexamethasone studies, one *α*-tocopherol succinate study, two methylprednisolone studies, one fluticasone propionate study, and one study on the (combination of) curcumin and tanshinone, the drugs were loaded on nanoparticles (Sun et al. 2015; Martin-Saldana et al. 2016; Martin-Saldana et al. 2017; Ramaswamy et al. 2017; Martin-Saldana et al. 2018; Yi et al. 2024; Mustafa et al. 2025) or encapsulated in hydrogel suspensions (Fernandez et al. 2016; Chen et al. 2019; Pierstorff et al. 2019; Dindelegan et al. 2024). All studies reported efficacy of the novel drug preparations as compared to both control arms without intervention as well as study arms with conventional drug formulations (Sun et al. 2015; Fernandez et al. 2016; Martin-Saldana et al. 2016; Martin-Saldana et al. 2017; Ramaswamy et al. 2017; Martin-Saldana et al. 2018; Chen et al. 2019; Pierstorff et al. 2019; Dindelegan et al. 2024; Yi et al. 2024; Mustafa et al. 2025). Novel delivery systems enhanced dexamethasone efficacy: hydrogel-based matrices (Chen et al. 2019; Dindelegan et al. 2024), Near-Infrared Responsive (NIR) nanocomposites (Mustafa et al. 2025), and the use of nanoparticle carriers (Sun et al. 2015; Chen et al. 2019) showed superior outcomes compared to conventional formulations. A microrobot-based strategy for ultrasound-triggered actuation and propulsion of a multi-drug solution of curcumin and tanshinone loaded on nanoparticles into the inner ear resulted in a ≥20 dB threshold difference between study arms in at least two adjacent measured frequencies (Yi et al. 2024) (Table 3).

**Table 3.** An overview of novel localized drug delivery methods.

Drugs	Delivery route	Animal model	Vehicle technology	Particle size	Advantage	Efficacy	≥20 dB	Ref
Dexamethasone	RWM application	Guinea pigs	PEG–PLA stealth nanoparticles	130 ± 4.78 nm	Continuous release of drug within 48 hours	+	–	Sun et al. (2015)
Dexamethasone	IT injection	Guinea pigs	poloxamer hydrogel OTO-104	NA	Sustained drug delivery due to a thermally reversible glycol polymer that turns into a gel at body temperature.	+	–	Fernandez et al. (2016)
Dexamethasone	Bullostomy	Wistar rats	pH-sensitive polymeric nanoparticles	MTOS HEI14: 181 ± 11 nm, MVE HEI14: 211 ± 36 nm	Enhanced encapsulation and retention, targeted delivery, and drug release	+	–	Martin-Saldana et al. (2018)
Dexamethasone	RWM injection	Mice	Silk-polyethylene glycol hydrogel	NA	High-affinity towards the RWM	+	–	Chen et al. (2019)
Dexamethasone	IT injection	Guinea pigs	NIR-responsive nanocomposite functionalized with saponin	162.57 ± 1.20 nm	Drug release upon NIR contact. Saponin enhances RW permeability	+	+	Mustafa et al. (2025)
Dexamethasone	IT injection	Rats	Hydrogel Matrix Containing Microcarriers	25 µm (Dindelegan et al. (2022))	Prolonged release of the drug up to 6 days	+	+	Dindelegan et al. (2024)
Dexamethasone, *α*-Tocopheryl	Bullostomy	Wistar rats	pH-sensitive polymeric nanoparticles	Range: 122.3 ± 5.98– 138.2 ± 7.17 nm	Enhanced encapsulation and retention, targeted delivery, and drug release	+	–	Martin-Saldana et al. (2017)
Methylprednisolone	Bullostomy	Wistar rats	pH-sensitive polymeric nanoparticles	Range: 120.8 ± 9.6 – 128.1 ± 7.4 nm	Enhanced encapsulation and retention, targeted delivery, and drug release	+	–	Martin-Saldana et al. (2016)
Methylprednisolone	IT injection	CBA/CAJ mice	Magnetic delivery of iron-coated nanoparticles	300 nm	Increase drug contact with RWM using magnetic delivery	+	–	Ramaswamy et al. (2017)
Fluticasone propionate	RWM puncture	Guinea pigs	thermally modified polyvinyl alcohol polymer OR-102	45–212 μm	Extended drug release	+	NA	Pierstorff et al. (2019)
Curcumin + Tanshinone	IT injection	Guinea pigs	Magnetic/acoustic dual-controlled microrobots	CS NPs: 369.7 ± 6.8 nm	Directional control of the flow of drug with a magnetic field. Ejection of drugs into the inner ear, overcoming the RWM	+	+	Yi et al. (2024)
L-*N*-acetylcysteine	IT injection	Guinea pigs	PLGA/PEGnanocapsules	–	Retain a higher concentration of the drug in the middle ear	+	–	Mohan et al. (2014)
			thermosensitive chitosan hyaluronic acid hydrogel	NA				
Diltiazem	IT injection	CBA/J mice	Chitosan hydrogel	NA	Sustained and controlled drug delivery	+	+	Naples et al. (2020)
L-arginine + Calmodulin inhibitor	IT injection	SD Rats	high-adhesion injectable hydrogel microsphere drug delivery system GH@PDA@LK	L-Arg: 168.30 ± 23.72 μm KN93: 192.06 ± 31.86 μm	Increased retention in the middle ear due to high-adhesion, high-affinity towards the RWM	+	+	Zhou et al. (2025)
rhBDNF	IT injection	Wistar rats	Poloxamer hydrogel POX407	NA	Sustained drug delivery due to thermal reversibility that turns into a gel at body temperature.	+	–	Pisani et al. (2025)
*α*-lipoic acid	IT injection	Mice	bionic adhesive and NIR-responsive PDA nanogel	87.6 ± 5.0 µm	Precise drug release upon NIR contact. Increased retention time in the middle ear	+	+	Chen et al. (2025)
Astaxanthine	IT injection	Mice	Poloxamer hydrogel POX407	NA	Sustained drug delivery due to thermal reversibility that turns into a gel at body temperature.	+	+	Nan et al. (2022)

RWM: round window membrane, IT: intratympanic, IP: intraperitoneal, SD Rats: Sprague–Dawley rat, PEG–PLA: polyethylene glycol-coated polylactic acid, NIR: near‐infrared responsive, PLGA: polylactic-co-glycolic acid, PEG: polyethylene glycol, GH@PDA@: methacrylate-gelatin microsphere (GH) conjugate polydopamine (PDA), PDA: polydopamine.

In summary, with seventeen preclinical studies, dexamethasone and methylprednisolone have been the most extensively investigated anti-inflammatory agents, and dexamethasone was repeatedly reported to be efficacious with a ≥20 dB threshold difference between study arms in at least two adjacent measured frequencies. Notably, several novel drug delivery systems showed a more promising effect than conventional formulations. However, these findings have not been independently replicated.

#### Chemical deactivators

Sixteen preclinical studies evaluated eleven different local chemical deactivators as protective agents against CIHL. This included *N*-acetylcysteine (NAC) (Choe et al. 2004; Saliba et al. 2010; Nader et al. 2010; Mohan et al. 2014; Chen et al. 2022), D- and L-methionine (Li et al. 2001; Korver et al. 2002; Wimmer et al. 2004), dimethyl sulfoxide (Roldan-Fidalgo et al. 2014), lactate (Choe et al. 2004; Nader et al. 2010), erdosteine (Saliba and El Fata 2012), thiourea (Ekborn et al. 2003), copper sulfate (More et al. 2010), and caspase inhibitors (Wang et al. 2004). One study investigated the role of pH modulation in the perilymph (Tanaka et al. 2004), and two studies assessed local cooling of the ear (Spankovich et al. 2016; Stanford et al. 2021). Guinea pigs were the most studied (13 reports), followed by rats (2 reports) (Li et al. 2001; Tanaka et al. 2004), mice (1 report), and chinchillas (1 report) (Korver et al. 2002; More et al. 2010). Audiological evaluations included ABR, DPOAE, hair cell count, or compound action potential (CAP) (Supplementary file 1. Table 2B).

NAC was the most frequently studied agent, having been tested in five studies, but with contradictory results. One study reported the efficacy of 2% NAC in guinea pigs, while 4% NAC had worse threshold shifts than the control arm (Chen et al. 2022). One study found moderate otoprotection with 2% NAC diluted with Ringer's lactate following high-dose cisplatin in guinea pigs. In that study, Ringer's lactate was used as a control vehicle and provided near-complete OHC preservation in DPOAE measurements (Choe et al. 2004). Intratympanic lactate was compared to intratympanic 20% NAC injections in a subject vs. subject model in guinea pigs, and lactate showed a superior otoprotective effect in ABR measurements at lower frequencies and met the review-defined otoprotection criterion (Nader et al. 2010). Intratympanic 4% NAC demonstrated smaller threshold shifts when compared with intratympanic methylprednisolone in guinea pigs, although neither study arm had a significant otoprotective effect (Saliba et al. 2010). High NAC concentrations were associated with middle ear inflammation and reduced protective effects (Saliba et al. 2010; Nader et al. 2010). In sum, only 2% NAC met the review-defined otoprotection criteria in one study (Chen et al. 2022).

D-methionine and L-methionine demonstrated otoprotective effects in three studies. 25 mg/mL D-methionine administered by bullostomy met the review-defined otoprotection criteria in chinchillas (Korver et al. 2002). In one comparative study, the D-methionine-treated study arm had a superior otoprotective effect than fibroblast growth factor (FGF-2), brain-derived neurotrophic factor (BDNF), and STS study arms (Wimmer et al. 2004). Dimethyl sulfoxide and erdosteine were found not to be otoprotective (Saliba and El Fata 2012; Roldan-Fidalgo et al. 2014), while thiourea and copper sulfate were reported to have protective effects in individual studies (Ekborn et al. 2003; More et al. 2010). One study compared the local application of caspase inhibitors via an osmotic pump in the base of the cochlea of guinea pigs. Caspase-3 and -9 inhibitors provided protection with ≥20 dB threshold difference between study arms in all measured frequencies. On the other hand, caspase-8 and cathepsin B inhibitors did not have an otoprotective effect (Wang et al. 2004). Round window niche pH showed efficacy only at a basic pH of 9.0, while an acidic pH of 6.0 increased ABR thresholds. Two studies demonstrated that local cooling of the ear canal via irrigation or a cooling rod reduced CIHL significantly, with effects persisting even one month after treatment. Both studies met the review-defined otoprotection criteria with ≥20 dB threshold difference between study arms in at least two adjacent measured frequencies (Spankovich et al. 2016; Stanford et al. 2021).

In the chemical deactivators category, four studies used a mini pump on the RWM or the bulla for local administrations (Li et al. 2001; Ekborn et al. 2003; Wimmer et al. 2004; Wang et al. 2004), two studies administered agents via bullostomy (Korver et al. 2002; Tanaka et al. 2004), and 10 agents were delivered through intratympanic injection (Choe et al. 2004; Saliba et al. 2010; Nader et al. 2010; More et al. 2010; Saliba and El Fata 2012; Mohan et al. 2014; Roldan-Fidalgo et al. 2014; Chen et al. 2022). One study in this category studied the efficacy of L-NAC encapsulated in nanoparticles versus a thermally sensitive gel. Trans-tympanic 2% L-NAC encapsulated in polylactic-co-glycolic acid (PLGA)/polyethylene glycol (PEG) nano-capsules in a thermosensitive chitosan hyaluronic acid hydrogel had better hearing outcome than L-NAC given in a thermosensitive chitosan hyaluronic acid hydrogel in guinea pigs at 20 kHz. However, neither group reported significant otoprotection, and both groups reported similar auditory degradation to the cisplatin group (Mohan et al. 2014).

In summary, while NAC at low concentrations (2%) and D-methionine showed consistent otoprotective effects, most other chemical deactivator agents studied lack replication. Local cooling of the ear canal emerged as a promising otoprotective strategy (Spankovich et al. 2016; Stanford et al. 2021).

#### Calcium blockers

In the calcium blocker category, five studies, all administered through intratympanic injections, investigated the efficacy of four otoprotective agents. Three preclinical studies evaluated intratympanic diltiazem as a local otoprotective agent (Naples and Parham 2016; Naples et al. 2018; Naples et al. 2020) (Supplementary file 1. Table 2C). Study animals included guinea pigs (3 reports) and Sprague–Dawley rats (SD rats) (1 report). Two studies applied diltiazem (2 or 4  mg/kg) daily for five days after cisplatin administration (8 mg/kg or 14 mg/kg) to mice and guinea pigs and reported a potential effect (Naples and Parham 2016; Naples et al. 2018). In a more recent study, a single intratympanic injection of 2 mg/kg diltiazem in a chitosan hydrogel preserved the hearing of mice across all measured frequencies with ≥20 dB threshold difference between study arms in two adjacent measured frequencies (Naples et al. 2020). One study investigated memantine, an *N*-methyl-D-aspartate (NMDA) receptor antagonist. While ABR results showed no audiological benefit, SEM and light microscopy suggested a potential protective effect of hair cells (Guven et al. 2025).

In a recent study, to improve local drug retention and diffusion, methacrylate-gelatin microspheres coated with polydopamine (GH@PDA), loaded with 50 mg/kg L-arginine and 10 mg/kg calmodulin-dependent kinase II inhibitor (KN93), were used. This formulation preserved hearing and hair cell integrity in Sprague–Dawley (SD) rats with ≥20 dB threshold difference between study arms in at least four adjacent measured frequencies, while the conventional 50 mg/kg L-arginine formulation study arm and the conventional 10 mg/kg KN93 formulation did not show an otoprotective effect (Zhou et al. 2025). None of the studies reported safety data or adverse effects related to calcium channel blockers.

In summary, diltiazem loaded on chitosan hydrogel showed better efficacy compared to diltiazem in 0.9% normal saline formulation. Only the chitosan hydrogel formulation of diltiazem (Naples et al. 2020) and multi-drug administration of l-arginine and KN93 loaded on GH@PDA had ≥20 dB threshold difference between study arms in at least two adjacent measured frequencies (Zhou et al. 2025).

#### Biologicals

The biologicals category included thirteen studies exploring six agents that were envisaged to mitigate CIHL via gene silencing (e.g. siRNA), viral-mediated delivery of genes encoding otoprotective proteins, local administration of brain-derived nerve growth factor (BDNF), glial cell line-derived neurotrophic factor (GDNF), platelet-rich plasma (PRP), and umbilical cord-derived mesenchymal stromal cells (UCMSCs). Study animals included Wistar rats (6 reports), guinea pigs (3 reports), SD rats (2 reports), mice (1 report), and rats (1 report). (Supplementary file 1. Table 2D).

Five studies evaluated small interfering RNAs (siRNAs) targeting oxidative stress and inflammatory pathways, including NADPH oxidase isoform NOX3 (Mukherjea et al. 2010), STAT1 (Kaur et al. 2011), cannabinoid receptor CB2R (Ghosh et al. 2018), adenosine receptor R-PIA (Sheehan et al. 2018), and chemokine receptor CXCR2 (Al Aameri et al. 2023). siRNAs were administered to Wistar rats via intratympanic injections 48 hours prior to a single intraperitoneal cisplatin dose (11 or 13 mg/kg). ABR thresholds were measured three days later. NOX3 siRNA at 0.3 µg already provided hearing protection, while higher doses (0.6–0.9 µg) showed near-complete preservation of OHCs (Mukherjea et al. 2010). STAT1 siRNAs yielded full auditory and structural otoprotection with ≥20 dB threshold difference between study arms at two adjacent measured frequencies (Kaur et al. 2011). CB2R, R-PIA, and CXCR2 siRNAs exhibited ≥20 dB threshold difference between study arms at only 32 kHz, but this effect dissipated at 16 and 8 kHz (Ghosh et al. 2018; Sheehan et al. 2018; Al Aameri et al. 2023). Three studies assessed the RWM application of adeno-associated virus (AAV)-mediated delivery of the X-linked inhibitor of apoptosis protein (XIAP) for otoprotection (Supplementary file 1. Table 2D). In two studies from the same laboratory, RWM injection of AAV-XIAP, XIAP, and dXIAP significantly reduced ABR threshold shifts and OHC loss after cisplatin exposure, with ≥20 dB threshold difference between study arms at 16 and 32 kHz (Cooper et al. 2006; Chan et al. 2007). The third study used a minimally invasive method with ≥80% inner hair cell transfection, 7-day cisplatin exposure, and 10 days post-transfection. ABR threshold shifts were reduced by 22 dB SPL, averaged from measured frequencies between 4–16 kHz, and hair cell loss was lower compared to the control arm by 140 counts averaged throughout the cochlea (Jie et al. 2015). In one study, PRP was administered trans-tympanically to cisplatin-treated rats. While threshold shifts were significantly lower in controls after 3 weeks, both study arms showed deterioration of ABR thresholds compared to baseline (Yurtsever et al. 2020). GDNF, delivered via intratympanic injection or Alzet minipump, doubled OHC counts in treated guinea pigs in one study (Kuang et al. 1999). Exosomes from UCMSCs, administered via RWM injection, significantly reduced ABR threshold shifts at 12 kHz and supported OHC preservation in mice. The presumed mechanism involved delivery of miRNAs that enhance endogenous growth factor production, thereby reducing cochlear damage (Tsai et al. 2021). Two studies evaluated BDNF in guinea pigs. In one study, one-month post-cisplatin delivery of BDNF via cochleostomy resulted in no ABR recovery, and the surgical procedure itself caused significant hearing damage in controls (Meen et al. 2010). In contrast, in the second study, recombinant human BDNF (rhBDNF) suspended in poloxamer thermos hydrogel POX407 and intratympanically administered 1 hour post-cisplatin infusion to Wistar rats significantly preserved ABR thresholds after 7 days at all tested frequencies (Pisani et al. 2025).

In the biological category, siRNAs, rhBDNF, and PRP were administered through intratympanic administration (Mukherjea et al. 2010; Kaur et al. 2011; Ghosh et al. 2018; Sheehan et al. 2018; Yurtsever et al. 2020; Al Aameri et al. 2023; Pisani et al. 2025), viral deliveries and UCMSCs through RWM application (Cooper et al. 2006; Chan et al. 2007; Jie et al. 2015; Tsai et al. 2021), GDNF through an Alzet minipump (Kuang et al. 1999), and BDNF though cochleostomy (Meen et al. 2010). Cochleostomy involves the creation of a surgical opening in the cochlea to allow direct intracochlear administration into the scala tympani (Borenstein 2011) (Figure 2).

In summary, AAV-mediated XIAP delivery was the only biological intervention with recurrently reported efficacy in studies that met the review-defined otoprotection criterion (Cooper et al. 2006; Chan et al. 2007; Jie et al. 2015). Moreover, siRNAs (NOX3, STAT1) demonstrate intriguing results.

#### Miscellaneous compounds

Fourteen preclinical studies evaluated an additional thirteen miscellaneous compounds with proposed otoprotective mechanisms not covered by other categories. Each compound was assessed in one study only: adenosine receptor agonists (Whitworth et al. 2004), epicatechin (Lee et al. 2010), vitamin C (Celebi et al. 2013), KR-22332 (Shin et al. 2014), melatonin (Demir et al. 2015), lutein (Roldan-Fidalgo et al. 2016), oxytocin (Bekmez Bilmez et al. 2016), CDK2 inhibitors (Hazlitt et al. 2018; Teitz et al. 2018), folic acid (Tanyeli et al. 2019), astaxanthin (Nan et al. 2022), tauroursodeoxycholic acid (Wen et al. 2021), and *α*-lipoic acid was investigated in two studies (Ozkul et al. 2014; Chen et al. 2025). Eleven studies used a single cisplatin dose (10–16 mg/kg), while five applied multi-day dosing (Bekmez Bilmez et al. 2016; Tanyeli et al. 2019; Wen et al. 2021; Nan et al. 2022; Chen et al. 2025). Study animals consisted of Wistar rats (5 reports), mice (3 reports), SD rats (2 reports), guinea pigs (1 report), chinchillas (1 report), and rats (1 report). (Supplementary file 1. Table 2E).

Both adenosine agonists, R-PIA and CCPA, significantly reduced ABR threshold shifts in chinchillas with ≥20 dB threshold difference between study arms in four and five adjacent measured frequencies, respectively (Whitworth et al. 2004). ABR threshold measurements revealed CDK2 inhibitors (kenpaullone, AT7519, AZD5438) protected against CIHL in mice and Wistar rats (Hazlitt et al. 2018; Teitz et al. 2018). Lutein was effective in vitro on HEI-OC1cell lines treated with cisplatin, but it did not show an otoprotective effect in ASSR measurements when administered intratympanically to Wistar rats (Roldan-Fidalgo et al. 2016).

Other compounds, astaxanthin (Nan et al. 2022), folic acid (Tanyeli et al. 2019), melatonin (Demir et al. 2015), vitamin C (Celebi et al. 2013), KR-22332 (Shin et al. 2014), tauroursodeoxycholic acid (Wen et al. 2021), epicatechin (Lee et al. 2010), and oxytocin (Bekmez Bilmez et al. 2016), were reported to provide a promising otoprotective effect.

In this category, adenosine agonists were administered through RWM application, while all other agents were applied through intratympanic injections. Only the efficacy of *α*-lipoic acid and astaxanthin was assessed with the application of novel drug delivery methods. Astaxanthin was intratympanically administered to cisplatin-treated mice in a polaxamer hydrogel (POX407) and was reported to yield significant otoprotection at measured frequencies with ≥20 dB at 16 kHz and 24 kHz (Nan et al. 2022). A-lipoic acid delivered via NIR-responsive microcarriers preserved OHCs and rescued hearing with ≥ 20 dB threshold difference between study arms at five adjacent measured frequencies (Chen et al. 2025), whereas conventional α-lipoic acid formulation administered with intratympanic injection showed no significant effect (Ozkul et al. 2014).

In summary, while agents in this category were only tested once, several promising otoprotective agents such as Kenpaullone (Teitz et al. 2018), tauroursodeoxycholic acid (Wen et al. 2021), adenosine receptor agonists (Whitworth et al. 2004), melatonin (Demir et al. 2015), α-lipoic acid (via a microcarrier) (Chen et al. 2025), and folic acid (Tanyeli et al. 2019) met the review-defined otoprotection criteria, and therefore merit further evaluation in preclinical models.

### Clinical studies of the efficacy and/or safety of local otoprotective agents

#### Dexamethasone

In total, six clinical studies, including four randomized clinical trials (RCTs), evaluated the efficacy and/or safety of intratympanic dexamethasone in cisplatin-exposed patients (*n* = 226) (Supplementary file 1. Table 3) (Marshak et al. 2014; Nasr et al. 2018; Sarafraz et al. 2018; Moreno and Belinchon 2022; Gupta et al. 2023; Freyer et al. 2025).

Two RCTs included cisplatin-treated adults (*n* = 38) with non-specified cancers (Marshak et al. 2014; Moreno and Belinchon 2022). Marshak et al. included cancer patients aged 38–80 years and reported minimal efficacy with an average of 6 dB threshold difference at 6 kHz of 10 mg/mL intratympanic dexamethasone (Marshak et al. 2014). In the second RCT, Moreno et al. used the Microwick passive diffusion device for the delivery of 8 mg of dexamethasone during cisplatin treatment over 24 hours in adult patients of 44–74 years of age with neoplastic diseases and reported no otoprotective effect. They described two cases of otorrhea and eight cases of permanent perforation among 23 patients treated with the Microwick device (Moreno and Belinchon 2022).

The only available pediatric RCT (*n* = 11) included patients with a median age of 4 years (0.5–14) (Freyer et al. 2025). The investigational drug was OTO-104, a thermosensitive poloxamer hydrogel designed for sustained dexamethasone release. From a total of eighteen intratympanic procedures, sixteen (89%) were done under sedation or anesthesia. The results revealed no audiological benefit, and the RCT was terminated early. Freyer et al. reported clinically insignificant scarring of the tympanic membrane in five participants and mild-moderate transient ear pain (CTCAE 2) in three participants and concluded that delivery was safe and feasible (Freyer et al. 2025).

The fourth RCT, described by Sarafraz et al., compared bilateral intratympanic dexamethasone with bilateral intratympanic NAC injections in a combined cohort of pediatric and adult patients aged 6–60 years. The authors reported no significant benefit of bilateral intratympanic dexamethasone compared to bilateral intratympanic NAC. They documented twenty cases of tinnitus in patients treated with bilateral dexamethasone (Sarafraz et al. 2018).

In two observational clinical studies, adult patients were given unilateral intratympanic dexamethasone injections. One study consisted of 20 patients with various neoplastic diseases (Nasr et al. 2018), while the other included 100 participants with head and neck cancer (Gupta et al. 2023). Nasr et al. reported statistically significant differences in threshold shifts with an average of 13 dB at 6 kHz and 12 dB at 8 kHz of intratympanic dexamethasone administered shortly after the detection of CIHL. However, this effect was not consistent for lower frequencies (Nasr et al. 2018). Gupta et al. reported statistically significant differences in threshold shifts between the study ears and the control ears at 6 kHz, with an average of 4 dB and 6 dB at 8 kHz. However, the effect was inconsistent in lower frequencies (Gupta et al. 2023).

#### N-acetylcysteine

One RCT in a combined pediatric and adult cancer cohort (6–60 years of age) (Sarafraz et al. 2018) and two clinical observational studies in adults investigated the effect of intratympanic NAC on the prevention of CIHL (*n* = 88) (Supplementary file 1. Table 3) (Riga et al. 2013; Yoo et al. 2014). Across studies, patients received 2–6 cycles of cisplatin with varying NAC and cisplatin dosages.

The RCT of Sarafraz et al. compared NAC with dexamethasone in 57 patients receiving bilateral intratympanic injections before cisplatin (Sarafraz et al. 2018). Riga et al. administered 10% NAC diluted in Ringer's lactate during hydration in 20 patients (Riga et al. 2013). NAC was reported to have protective effects in both studies (*n* = 77), with average reduced threshold shifts of 7 and 12 dB at 8 kHz. However, the effect was inconsistent in lower frequencies (Riga et al. 2013; Sarafraz et al. 2018). In another RCT, Yoo et al. included 11 cisplatin-treated patients receiving L-NAC via myringotomy, 30–60 minutes before cisplatin infusion. However, L-NAC was less effective, with only two out of 11 patients showing measurable benefit (Yoo et al. 2014). No serious adverse events with NAC administration were reported.

In summary, none of the RCTs and clinical observational studies on dexamethasone or NAC reported a clear, impactful otoprotective effect.

## Discussion

A total of 45 agents were evaluated across 70 studies, with 40 agents reported to have at least a partial otoprotective effect. The large number of studies and the diverse range of drugs studied for local otoprotection reflect the urgent clinical need for otoprotection in patients treated with cisplatin. Despite the large number of preclinical studies and tested drugs, only dexamethasone, AAV-mediated XIAP, and thermal treatment of the ear canal reported efficacy in multiple studies that met the review-defined otoprotective criterion with ≥20 dB threshold difference between study arms at two adjacent measured frequencies (Table 4).

**Table 4.** Summary of the most promising localized otoprotective agents.

Otoprotective agent	No. of studies	Mechanism	Best available evidence	Efficacy summary
Dexamethasone	17 animal and 6 clinical studies	Anti-inflammatory effect	RCT	Significant otoprotection in preclinical studies, but inconsistent clinical benefit
*N*-acetylcysteine	5 animal and 3 clinical studies	Free-radical scavenging molecule	RCT	Mixed results with limited clinical confirmation
AAV-Mediated XIAP	2 animal studies	Apoptosis inhibition	Animal study	Significant otoprotective effect and outer hair cell preservation
Thermal treatment of the ear canal	2 animal studies	Reduction of cochlear cell cisplatin uptake, Reduced production of reactive oxygen species.	Animal study	Significant otoprotective effect up to two hours before cisplatin administration. Damage observed after consecutive cisplatin doses.

AAV: adeno-associated viral vector, XIAP: X-linked inhibitor of apoptosis protein, RCT: Randomized controlled trial.

Dexamethasone was reported efficacious consistently when delivered via novel technologies and vehicles rather than the conventional drug formulation. NIR-responsive nanoparticles increased RWM permeability of dexamethasone, while hydrogel matrices increased dexamethasone retention time in the middle ear (Dindelegan et al. 2024; Mustafa et al. 2025). Despite the efficacy of RWM application of AAV-mediated XIAP delivery in three preclinical studies, there are several challenges and concerns for the implementation of this intervention in a clinical setting (Cooper et al. 2006; Chan et al. 2007; Jie et al. 2015). An overexpression of XIAP could lead to impaired apoptosis and could have an oncogenic effect (Hussain et al. 2017; Li et al. 2024). Moreover, a pre-existing or therapy-induced immune response to AAV vectors could drastically reduce the efficacy of the intervention (Zaiss and Muruve 2005; Ronzitti et al. 2020). Ear canal cooling is reported to be a successful non-invasive otoprotective strategy in two independent different animal studies. Cooling has been extensively studied as a non-invasive method to reduce chemotherapy-induced hair loss, skin toxicities, peripheral neuropathy, and nail toxicity (Ladwa et al. 2024; Yodchai et al. 2024; Mallela et al. 2025; Bandla et al. 2025). The otoprotective value of ear canal cooling is yet to be tested in clinical trials.

Intratympanic Ringer's lactate demonstrated an unexpected otoprotective effect when used as a control condition in pilot experiments, which led to two studies specifically investigating its potential protective properties (Choe et al. 2004; Nader et al. 2010). Although the precise mechanism of this effect remains unclear, it has been hypothesized that lactate may exert antioxidant activity through the regeneration of nicotinamide adenine dinucleotide (NADH). In addition, the relatively low molecular weight of lactate may facilitate diffusion across the RWM, potentially enhancing inner ear availability (Choe et al. 2004).

Seventeen preclinical studies investigated the effect of drug delivery methods, including novel nanoparticle delivery, hydrogel matrices, and ultrasound and NIR-responsive to deliver 14 different agents to the cochlea; all reported significant prevention of CIHL. This observation suggests the relative importance of novel drug delivery methods and delivery vehicles (Table 3).

Despite the large number of preclinical studies, only dexamethasone and *N*-acetylcysteine were investigated in clinical settings (Table 2). While 15 out of 17 preclinical dexamethasone studies and 3 out of 5 preclinical NAC studies reported positive results, none of the clinical trials reported a meaningful otoprotective effect that met the review-defined otoprotection criteria. One possible reason for the low efficacy of dexamethasone and NAC in clinical studies might originate from the loss of aqueous solution through the Eustachian tube (Marshak et al. 2014; Yoo et al. 2014; Moreno and Belinchon 2022), which may reduce middle ear residence time and limit cochlear exposure. Several studies included in this review have therefore explored carrier-based strategies, such as hydrogels or nanoparticle formulations, to prolong drug retention in the middle ear and promote sustained diffusion across the RWM (Table 3). While these approaches show promise in improving local drug availability, further studies are needed to optimize formulation characteristics, evaluate long-term safety, and determine their translational feasibility in clinical settings.

The difference in otoprotective effect between preclinical and clinical settings is consistent with reported literature that animal experiments often poorly predict human response to therapeutic exposures (Bracken 2009; Van Norman 2019). A similar pattern has been observed in studies evaluating the efficacy of STS in preclinical and clinical settings (Streefkerk et al. 2025). Beyond fundamental interspecific differences, including cochlear anatomy, pharmacokinetics, drug delivery dynamics, and genetic variability, several factors contribute to this translational gap. First, experimental conditions differ substantially from clinical practice, as animal studies frequently use acute, single, high-dose cisplatin exposure, whereas patients typically receive multiple cisplatin cycles over time, often in combination with other treatments. In addition, inter-individual variability in susceptibility to CIHL further complicates interpretation. Second, candidate otoprotective agents should not compromise the antitumor efficacy of cisplatin, which substantially restricts the range of viable therapeutic strategies. Finally, a major limitation is the difficulty of measuring drug pharmacokinetics within the human inner ear, making it challenging to determine whether drug concentrations shown to be protective in animal studies are achieved in the cochlea of treated patients (Bracken 2009; Van Norman 2019; Yu et al. 2025; Iațentiuc et al. 2025; Orasan et al. 2025).

Related, such challenges restricted cross-study comparison in this review. We adopted the Greenwood function to estimate relevant human equivalent frequency ranges of study animals and defined an otoprotective criterion (≥20 dB threshold difference in at least two adjacent measured frequencies) for harmonized comparison. Conformance to available universal guidelines for the design of an animal experiment and the guidelines on the reporting of animal research in the ototoxicity field could greatly enhance cross-study comparison (Bregman et al. 2003; Services et al. 2019; Percie du Sert et al. 2019; Domarecka et al. 2021; Domarecka and Szczepek 2023; Manickam and Zallocchi 2025).

Beyond methodological considerations, the findings of this review also highlight that route and method of administration may influence otoprotective efficacy. Although non-surgical methods such as intratympanic injections represent a more suitable procedure in a clinical setting, animal study outcomes do not fully translate to human studies, as previously discussed (El Kechai et al. 2015; Streefkerk et al. 2025). On the other hand, surgical approaches such as RWM application enable direct drug delivery into the inner ear and may ensure sufficient inner ear drug concentrations. However, these methods are highly invasive and hard to implement when a patient requires multiple doses of an otoprotective solution (Meen et al. 2010). This may suggest that agents that have a long half-life and are consequently retained in the anatomical site of interest may enable single applications, potentially impacting clinical feasibility.

In addition to efficacy, the safety profile of local drug delivery approaches should be considered. Across the included clinical studies, intratympanic administration was generally well tolerated. Reported adverse events were mostly mild and transient and included short-lasting pain during or shortly after injection, transient vertigo, and tinnitus (Marshak et al. 2014; Sarafraz et al. 2018; Gupta et al. 2023). In a minority of cases, otorrhea and permanent tympanic membrane perforation were reported (Moreno and Belinchon 2022). Loss of the injected solution through the Eustachian tube was also described as an unavoidable limitation of intratympanic administration. In one study, clinically insignificant tympanic membrane scarring and mild-to-moderate transient ear pain (CTCAE grade 2) were observed, but the procedure was still considered safe and feasible (Yoo et al. 2014; Freyer et al. 2025). Overall, these findings suggest that intratympanic delivery is generally well tolerated, although standardized reporting of adverse events remains limited.

For pediatric patients receiving cisplatin, the successful implementation of local otoprotective strategies will require several translational advances. First, well-designed clinical trials in cisplatin-treated pediatric populations are needed to determine the real-world efficacy and safety of local interventions (Meijer et al. 2024; Freyer et al. 2025). In parallel, the development of predictive models to identify children at high risk of CIHL may help target patients most likely to benefit from such procedures (Millstein et al. 2025). In addition, greater consensus on optimal application routes and treatment protocols is required. From a technical perspective, device development should prioritize minimally invasive delivery systems that minimize tympanic membrane trauma, such as sustained-release gels or diffusion-based carriers. Finally, practical clinical implementation will require streamlined procedural logistics, including adequate sedation, pain control, and immobilization, as well as behavioral management techniques such as preparation, distraction, coaching, and reinforcement to promote cooperation to ensure that local delivery can be performed with minimal burden for pediatric patients and caregivers (Cohen et al. 2015, 2022).

## Conclusions

In conclusion, the variety and large number of agents investigated for their otoprotective properties illustrate the need for prevention of ototoxicity due to cisplatin treatment. Although we found multiple agents exhibiting significant potential in preventing CIHL in a preclinical setting, our review did not identify available studies of local alternative otoprotective agents that could reliably replace systemic STS in terms of safety and efficacy to be considered for pediatric patients. Novel drug delivery methods, including hydrogel matrices and nanoparticles, may enhance the otoprotective effect in clinical setups. Further research into the optimal dosages, effective delivery to the middle ear, and timing of delivery is necessary before these drugs and delivery methods can become part of standard clinical practice.

## Supplementary Material

Supplementary_File_2 Supplementary_File_2

Suplementary_file_1_drug_delivery.docx Suplementary_file_1_drug_delivery.docx

## Data Availability

Data sharing is not applicable to this article as no new data were created or analyzed in this study.

## Abbreviations

AAV: adeno-associated virus
ASSR: auditory steady-state response
BDNF: brain-derived neurotrophic factor
CAP: compound action potential
CB2R: cannabinoid receptor
CDK2: cyclin-dependent kinase 2
CIHL: cisplatin-induced hearing loss
CXCR2: chemokine receptor
dB: decibel
DPOAE: distortion otoacoustic emissions
EMA: European Medicine Agency
FDA: Food and Drug Administration
FGF-2: fibroblast growth factor
GDNF: glial cell line-derived neurotrophic factor
GH@PDA: methacrylate–gelatin microspheres coated with polydopamine
HEFR: human equivalent frequency range
KN93: calmodulin-dependent kinase II inhibitor
MHRA: Medicines and Health Regulatory Authority
NAC: N-acetylcysteine
NIR: near-Infrared response
NMDA: N-methyl-D-aspartate
NOX3: NADPH oxidase isoform
OHC: outer hair cell
PEG: polyethylene glycol
PLGA: polylactic-co-glycolic acid
POX407: poloxamer thermos hydrogel
PRP: platelet-rich plasma
R-PIA: adenosine receptor
RCT: randomized controlled trial
rhBDNF: recombinant human Brain-derived neurotrophic factor
ROS: reactive oxygen species
RWM: round window membrane
SD rat: Sprague–Dawley rat
SFR: sensitive frequency range
siRNA: small interfering RNA
SPL: sound pressure level
STAT1: signal transducer and activator of transcription-1
STS: sodium thiosulfate
TRPV1: transient receptor vanilloid
UCMSC: umbilical cord-derived mesenchymal stromal cells
XIAP: X-linked inhibitor of apoptosis protein
