# Supplementary material for: Local application of otoprotective compounds other than sodium thiosulfate to prevent cisplatin-induced hearing loss: a systematic review
Source: Drug Deliv. 2026 May 1;33(1):2665892. doi: 10.1080/10717544.2026.2665892 (PMC13137753; doi:10.1080/10717544.2026.2665892)
Supplement: Suplementary_file_1_drug_delivery.docx [file IDRD_A_2665892_SM4292.docx]

**Supplemental tables and figures**

Supplementary table 1. Mesh terms, used in the search strategy.

| **Search Terms:** |
| --- |
| (("ototoxicity"[MeSH Terms] OR "ototox*"[Title/Abstract] OR "otol*"[Title/Abstract] OR "cochleotox*"[Title/Abstract] OR "vestibulotox*"[Title/Abstract] OR "cochlear toxic*"[Title/Abstract] OR "vestibular toxic*"[Title/Abstract] OR "tinnitus"[MeSH Terms] OR "tinnitus"[Title/Abstract] OR "hearing imp*"[Title/Abstract] OR "hearing loss"[MeSH Terms] OR "hearing loss*"[Title/Abstract] OR "hearing"[Title/Abstract] OR "deaf*"[Title/Abstract] OR "vertigo"[MeSH Terms] OR "vertigo"[Title/Abstract] OR "dizz*"[Title/Abstract] OR "audio*"[Title/Abstract]) AND("cisplatin"[Title/Abstract] OR "platinum"[Title/Abstract] OR "platin"[Title/Abstract]) AND (“otoprotect*”[Title/Abstract] OR “oto-protect*”[Title/Abstract] OR “protect*”[Title/Abstract] OR “defend*”[Title/Abstract] OR “help*”[Title/Abstract] OR “preserv*”[Title/Abstract] OR “conserv*”[Title/Abstract] OR “support*”[Title/Abstract] OR “shield*”[Title/Abstract] OR “keep*”[Title/Abstract])) |

**
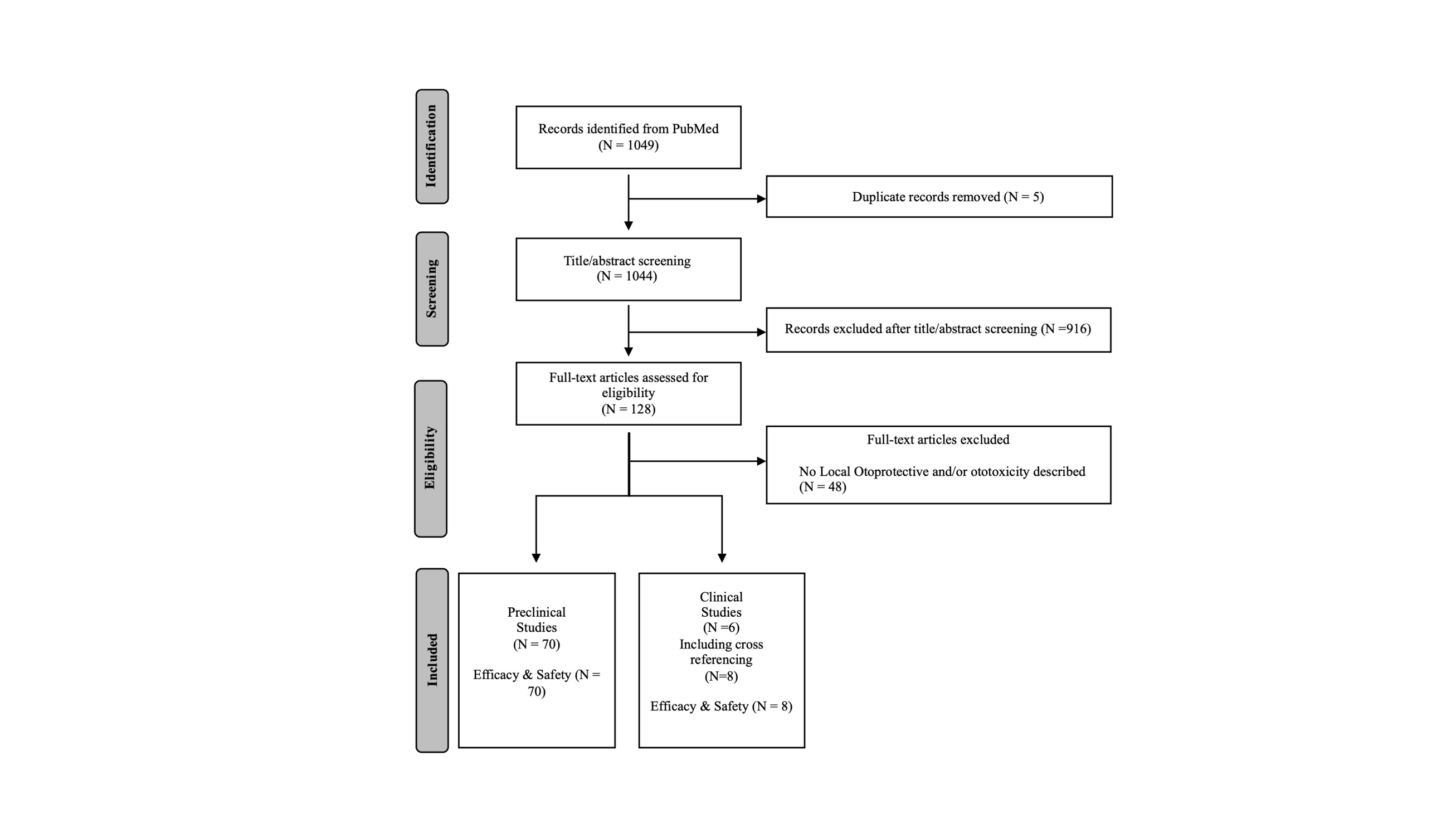
**

**Supplementary Figure 1. Flow diagram of study selection.**

Supplementary Table 2A. Preclinical studies on the efficacy and safety of locally administered anti-inflammatory otoprotective drugs.

| **Anti-inflammatory** | | | | | | | | | | | **End point Result** | | | **Conclusion** | | |
| --- | --- | --- | --- | --- | --- | --- | --- | --- | --- | --- | --- | --- | --- | --- | --- | --- |
| **Author, Year** | **Intervention drug** | **Model (age)** | **Design** | **Study arm, treatment (No. subjects)** | **Comparison arm, treatment (No. subjects)** | **Cisplatin dose, frequency** | **Intervention Drug Specification** | **Intervention Drug administration** | **Evaluation Method** | **FU time**  **post-Tx** | **Hearing function outcomes** | | **p-value** | **Efficacy** | **safety** | **≥20dB** |
|  |  |  |  |  |  |  |  |  |  |  | **With intervention drug** | **Without intervention drug** |  |  |  |  |
| **Daldal 2007 [1]** | Dex | Adult female Albino guinea pigs | 2 | Cis + Dex (n=6) | Cis + saline (n=6) | 12-16mg/kg IP, single dose | 4 mg/ml IT | IT injection | DPOAE | 3 d | 1kHz: 1.2 dB SPL*  1.4kHz: 13.1 dB SPL*  2.0kHz: 14.3 dB SPL*  2.8kHz:10.4 dB SPL*  **4kHz: 7.7 dB SPL***  **6kHz: 14.6 dB SPL*** | 1kHz: 8 dB SPL*  1.4kHz: 15.8 dB SPL*  2.0kHz: 15.5 dB SPL*  2.8kHz: 15.4 dB SPL*  **4kHz: 9.4 dB SPL***  **6kHz: 16.3 dB SPL*** | **0.000 0.000 0.000 0.000 0.000 0.000** | HC+  HC+  HC+  HC+  HC+  HC+ | + | NA |
| **Hill 2008**  **[2]** | Dex | (4-8 weeks) Mice | 1 | Cis + Dex (n=10) | Cis + saline (n=10) | 14mg/kg IP, single dose | 24mg/ml IT, daily for 5d | IT injection | Click evoked ABR | 5 d | Click: 2 ± 2 dB | Click: 14 ± 6 dB | **0.0005** | + | + | NA |
|  |  |  |  |  |  |  |  |  | Pure tone evoked ABR |  | PT 8kHz: 2 ± 4 dB  **PT 16kHz: 1 ± 2 dB**  **PT 32kHz: 23 ± 11 dB** | PT 8kHz: 8 ± 4 dB  **PT 16kHz: 11 ± 6 dB**  **PT 32kHz: 20 ± 17 dB** | **0.03**  **0.002**  0.8 | -  +  - |  | - |
| **Murphy 2011**  **[3]** | Dex | Adult female Albino guinea pigs | 2  1 | I: Cis 10mg/kg + Dex BL (n=6) | I: Cis 12mg/kg + Dex BL (n=6) | 10mg/kg IP | I: 24mg/ml | IT injection | ABR threshold shifts | 3 d | Baseline:  **8kHz: 38± 3.4 dB SPL*, 16kHz:34± 2.4 dB SPL*,**  25kHz:27± 3.1 dB SPL | |  | **++**  **+**  **-** | + | - |
|  |  |  |  |  |  | 12mg/kg IP |  |  |  |  | 10mg/kg cisplatin - day 3:  **8kHz: 60± 7.6 dB SPL***  **16kHz:78.3± 8.2 dB SPL***  25kHz:79.5± 7 dB SPL* | 12mg/kg cisplatin - day 3:  **8kHz:84.6 ± 5.8 dB SPL***  **16kHz:91.5± 3.5 dB SPL***  25kHz:84.4 ± 2.4 dB SPL* | P<0.01  P<0.01  P<0.01 |  |  |  |
|  |  |  |  | II: Cis + Dex UL (n=26) | II: Cis + Saline UL (n=26) | II: 12mg/kg IP | II: 0.15mL  4mg/ml,  1d before and on same d as Cis |  |  |  | II: 4mg/ml:  **8kHz: 41.0 ± 4.9 dB SPL***  **16kHz: 50.7 ± 1.7 dB SPL***  25kHz: 37.6 ± 5.3 dB SPL* | II: 4mg/ml:  **8kHz: 36.2 ± 10.2 dB SPL***  **16kHz: 56.1 ± 9.4 dB SPL***  25kHz: 38.8 ± 8.0 dB SPL* | P= 0.66 | -  -  - |  | - |
|  |  |  |  |  |  |  | II: 0.15mL,  10mg/ml  1d before and on same d as Cis |  |  |  | 10mg/ml:  **8kHz: 59.8 ± 9.3 dB SPL***  **16kHz: 61.2 ± 5.4 dB SPL***  25kHz: 59.5 ± 4.8 dB SPL* | 10mg/ml:  **8kHz: 54.6 ± 5.3 dB SPL***  **16kHz:59.4 ± 5.7 dB SPL***  25kHz: 59.6 ± 6.2 dB SPL* |  | -  -  - |  | - |
|  |  |  |  |  |  |  | II: 0.15mL,  40mg/ml,  1d before and on same d as Cis |  |  |  | 40mg/ml:  **8kHz: 34.1 ± 7.6 dB SPL**  **16kHz: 50.7 ± 2.4 dB SPL***  25kHz: 48.1 ± 2.4 dB SPL* | 40mg/ml:  **8kHz: 37.4 ± 6.0 dB SPL***  **16kHz: 52.7 ± 4.7 dB SPL***  25kHz: 52.5 ± 3.6 dB SPL* |  | -  -  - |  | - |
| **Parham 2011**  **[4]** | Dex | 24-month-old CBA/J Mice | 1 | Cis + Dex (n=13) | Cis + Saline (n=13) | 14mg/kg IP, single dose | 24mg/ml IT, daily for 6d | IT injection | ABR threshold shift | 7 d | Baseline ABR:  8kHz: 69.2 dB*  **16kHz: 68.5 dB***  **24kHz: 72.5 dB***  **32kHz: 72.0 dB*** | | 0.36  0.075 0.026 **0.0006** | -  -  -  - | NA | - |
|  |  |  |  |  |  |  |  |  |  |  | Post study ABR: 8kHz: 74.9dB*  **16kHz: 74.8dB***  **24kHz: 72.9dB***  **32kHz: 73.3dB*** | Psot study ABR:  8kHz:75.7 dB*  **16kHz: 77.7 dB***  **24kHz: 77.7 dB***  **32kHz: 81.6 dB*** |  |  |  | - |
| **Shafik 2013 [5]** | Dex | adult male Guinea pigs | 2 | I: Dex 1h prior to Cis (n=6) | Cis only (n=6) | 8mg/kg IP, single dose | 4 mg/ml, daily for 5d  I: start 1h prior to Cis | IT injection | Click evoked ABR threshold shift | 5 d | I: 11.67 ± 1.67 dB | 58.33 ± 4.41 dB | **<0.05** | ++ | + | NA |
|  |  |  |  | II: Dex 1d prior to Cis (n=6) |  |  | 4 mg/ml, daily for 5d II: start 1d prior to Cis |  |  |  | II: 26.67 ± 3.33 dB |  | **<0.05** | ++ |  | NA |
| **Hughes 2014  [6]** | Dex | CBA/J mice (8-10  Weeks) | 1 | Cis + Dex (n=15) | Cis + Saline (n=15) | 3mg/kg/d IP, for 5 d | 24mg/ml | IT injection | ABR threshold shift | 7, 14, 21 + 28 d | Average difference in ABR threshold (saline – Dex):  D7: **8kHz: 2.7 dB; 16kHz: 1.7 dB;** 24kHz: 0.0 dB; 32kHz: 2.0 dB *  D14: **8kHz:-0.6 dB; 16kHz: 3.7 dB;** 24kHz: 1.7 dB; 32kHz: 1.3 dB *  D21: **8kHz:-2.9 dB; 16kHz:-0.3 dB;** 24kHz: 2.3 dB; 32kHz: 1.7 dB *  D28: **8kHz:-3.0 dB; 16kHz:-2.7 dB;** 24kHz: 0.0 dB; 32kHz: 2.0 dB * | | 0.67 | -  -  -  - | NA | - |
| **Sun 2015 [7]** | Dex | Guinea pigs | 2 | I: Cis + Dex (n=8) | Cis only (n=8) | 12mg/kg IP single dose | 5 µL, 10 mg/mL  I: Free dex | RWM application | ABR threshold shift,  Hair cell count | 3 d | I: **4kHz: 81.88 ± 6.51 dB**  **8kHz: 89.38 ± 6.23 dB**  I: HCC 42 ± 7%* | **4kHz: 85.63 ± 11.78 dB**  **8kHz: 88.13 ± 9.98 dB**  HCC: 36 ± 8%* | NS  NS  NS | -  - | + | - |
|  |  |  |  | II: Cis + Dex-NP (n=10) |  |  | 5 µL, 10 mg/mL II: dex-NP suspension |  |  |  | II: **4kHz: 63.5 ± 15.75 dB**  **8kHz: 69.00 ± 17.68 dB**  II: HCC 65 ± 11%* |  | **<0.01**  **<0.05**  **<0.001** | ++  + |  | - |
| **Fernandez 2016 [8]** | Dex | Guinea pigs  (6-8  Weeks) | 2 | Cis + Dex (n=6) | Cis only (n=6) | 4mg/kg, 3 weeks 1x/week | Dex: OTO-104 6.0%. 1x/week prior to Cis | IT injection | ABR threshold shift | 23 d | **4kHz: 10.0 ± 3.5 dB**  **10kHz: 7.5 ± 4.8 dB**  **16kHz: 11.3 ± 7.3 dB** | **4kHz: 27.5 ± 5.2 dB**  **10kHz: 26.3 ± 13.9 dB**  **16kHz: 41.3 ± 10.3 dB** | **0.022**  0.247  **0.041** | +  +  ++ | NA | - |
| **Martín-Saldaña 2018**  **[9]** | Dex | Wistar rats | 1 | I: Cis + MTOS HEI14-10Dx (n=8) | I: Cis + PBS (n=8) | 10 mg/kg IP | 50 ul, (2mg/ml) NP suspension 10% Dex | bullostomy | ASSR | 3 d | I: **8kHz: -4.6 ± 11.6 dB SPL***  **12kHz: 10.0 ± 10.8 dB SPL***  **16kHz: 10.0 ± 16.2 dB SPL***  **20kHz: 19.9 ± 4.5 dB SPL***  **24kHz: 5.2 ± 11.0 dB SPL***  **28kHz: 10.1 ± 8.8 dB SPL***  **32kHz: 15.2 ± 11.6 dB SPL*** | **I: 8kHz: 10.1 ± 8.2 dB SPL***  **12kHz: 26.6 ± 5.9 dB SPL***  **16kHz: 25.0 ± 13.0 dB SPL***  **20kHz: 30.0 ± 11.3 dB SPL***  **24kHz: 40.0 ± 17.0 dB SPL***  **28kHz: 30.0 ± 11.8 dB SPL***  **32kHz: 30.0 ± 5.1 dB SPL*** | >0.05  >0.05  **<0.05**  **<0.05**  **<0.05**  **<0.05**  **<0.05** | +  +  +  +  ++  +  + | NA | - |
|  |  |  |  | II: Cis + MVE HEI14-10Dx (n=8) | II: Cis + PBS (n=8) |  |  |  |  |  | **II: 8kHz: 5.1 ± 7.1 dB SPL***  **12kHz: 35.1 ± 7.1 dB SPL***  **16kHz: 49.9 ± 0.0 dB SPL***  **20kHz: 49.4 ± 25.5 dB SPL***  **24kHz: 40.0 ± 14.2 dB SPL***  **28kHz: 45.0 ± 6.9 dB SPL***  **32kHz: 43.5 ± 2.9 dB SPL*** | **II: 8kHz: 15.3 ± 6.8 dB SPL***  **12kHz: 35.1 ± 20.9 dB SPL***  **16kHz: 34.9 ± 7.3 dB SPL***  **20kHz: 44.5 ± 21.7 dB SPL***  **24kHz: 35.1 ± 7.6 dB SPL***  **28kHz: 30.0 ± 14.0 dB SPL***  **32kHz: 34.9 ± 7.2 dB SPL*** | >0.05  >0.05  >0.05  >0.05  >0.05  >0.05  >0.05 | +  -  -  -  -  -  - |  | - |
| **Chen 2019 [10]** | Dex | C57BL/6J Mice (8-weeks) | 2 | I: Cis + Dex-SILK (n=6) | I: Cis + drug-free SILK (n=6) | 12mg/kg IP, single dose | I: Dex-SILK mixture 1µl, 1 h prior to Cis | RWM application | ABR threshold,  Hair cell count (distance form apex) | 5 d | ABR:  I: 4kHz: 69.17±3.76 dB  8kHz: 59.17±4.92 dB  **16kHz: 64.17±7.36 dB**  HCC:  20%: 241  40%: 292  60%: 244  80%: 239  100%: 164 | ABR:  I: 4kHz: 87.50 ±4.18 dB  8kHz: 85.83±4.92 dB  **16kHz: 75.83 ±5.85 dB**  HCC:  20%: 131  40%: 122  60%: 154  80%: 52  100%: 148 | **<0.001**  **<0.001**  **<0.01** | +  ++  + | NA | - |
|  |  |  |  | II: Cis + Dex (n=4) | II: Cis only (n=6) |  | II: 8% Dex-gelfoam 1µl, 1 h prior to Cis |  |  |  | ABR: II: 4kHz: 90.6 dB*  8kHz: 85.6 dB*  **16kHz: 79.3 dB***  **16kHz: 81.4 dB***  HCC  20%: 247  40%: 151  60%: 249  80%: 249  100%: 127 |  | >0.05 | -  -  - |  | - |
| **Mustafa 2024 [11]** | Dex, AuNR@DEX-MS-saponin | Guinea pigs | 2 | Cis + Dex (n=10) | Sham IT (n=8) | 10mg/kg, hypodermic | Equ. 100µg/mL Dex | IT injection, 2d before Cis | ABR threshold, OHC loss *(no. turn) | 4d | ABR:  **4kHz: 75.5 ± 28.23 dB**  **8kHz: 71.5 ± 33.42 dB**  **16kHz: 76.5 ± 27.89 dB**  24kHz: 81.0 ± 21.58 dB  32kHz: 89.0 ± 11.50 dB  click: 78.0 ± 25.30 dB SPL  OHC loss:  1st:11%  2nd:7%  3rd:11%  4th:16% | ABR:  **4kHz: 81.87 ± 14.37 dB**  **8kHz: 80.62 ± 18.60 dB**  **16kHz: 83.75 ± 14.33 dB** 24kHz: 88.12 ± 10.32 dB 32kHz: 94.37 ± 1.76 dB  click: 81.25 ± 19.22 dB SPL  OHC loss:  1st:2%  2nd:12%  3rd:10%  4th: 14% | NS | ABR:  -  -  -  -  - | NA | - |
|  |  |  |  | Cis + Dex + Laser (n=10) |  |  |  |  |  |  | ABR:  **4kHz: 63.5 ± 22.24 dB**  **8kHz: 59.5 ± 30.13 dB**  **16kHz: 68.5 ± 26.36 dB**  24kHz: 80.5 ± 19.21 dB  32kHz: 86.0 ± 11.50 dB  click: 64.9 ± 20.31 dB SPL  OHC loss:  1st: 0.1%  2nd:1%  3rd:2%  4th:6% |  | NS | ABR:  +  ++  +  -  - | NA | - |
|  |  |  |  | Cis + NPs/DEX + DEX (n=12) | Cis + Sham IT |  |  |  |  |  | ABR:  **4kHz:62.08 ± 25.80 dB**  **8kHz:55.83 ± 31.47 dB**  **16kHz:65.83 ± 25.75 dB**  24kHz:72.92 ± 20.83 dB  32kHz:85.42 ± 10.76 dB  click:65.42 ± 23.78 dB SPL  OHC loss:  1st:1%  2nd:1%  3rd:3%  4th:3% |  | NS | ABR:  +  ++  +  +  - | NA | - |
|  |  |  |  | Cis + NPs/DEX + DEX + Laser(n=12) |  |  |  |  |  |  | ABR:  **4kHz:33.33 ± 7.49 dB**  **8kHz:24.58 ± 5.42 dB**  **16kHz:33.33 ± 7.18 dB**  24kHz:45.83 ± 12.94 dB  32kHz:63.75 ± 19.67 dB  click:35.83 ± 7.02 dB SPL  OHC loss:  1st:1%  2nd:0.5%  3rd:0.3%  4th:1% |  | ABR:  p <0.0001  p <0.0001  p <0.0001  p <0.0001  p <0.0001  p <0.0001  OHC:  p<0.05  P<0.01  P<0.05  P<0.001 | ABR:  ++  ++  ++  ++  ++ | NA | + |
| **Dindelegan 2024 [12]** | Dex | Male Wistar Rats  (8-9 weeks) | 1 | Lmp/Dex(n=5) | Dex(n=11) | 15mg/kg IP,  single dose | 30 µL, equv. 1.5 mg/ml | IT injection | ABR threshold | 7 d | **8kHz: 12.8 dB***  **16kHz: 30.9 dB***  **24kHz:0.3 dB***  **32kHz:16.8 dB***  Click: 1.6 dB* | **8kHz: 33.3 dB***  **16kHz: 47.5 dB***  **24kHz:25.3 dB***  **32kHz:51.5 dB***  Click:6.1 dB* | p>0.05  p>0.05  p>0.05  **p=0.0057**  p>0.05 | ++  +  ++  ++  - | NA | + |
|  |  |  |  | Vitrogel-Lmp/Dex(n=6) |  |  |  |  |  |  | **8kHz: 6.4 dB***  **16kHz: 8.0 dB***  **24kHz:4.8 dB***  **32kHz:9.9 dB***  Click:0 dB* | **8kHz: 38.7 dB***  **16kHz: 48.5 dB***  **24kHz:26.1 dB***  **32kHz:45.3 d**B*  Click:9.4 dB* | p=0.0004  p<0.0001  p=0.0302  **p<0.0001**  p>0.05 | ++  ++  ++  ++  - |  | + |
| **Martín-Saldaña 2016**  **[13]** | Pred | Wistar rats | 1 | I: Cis + NP-MVE-0 (n=2) | Cis + PBS (n=22) | 10 mg/kg IP | 50µl of NP solution  I: NP – vit E, no pred | Bullostomy | ASSR | 72 h | I: 1kHz: 72.5 ± 7.4 dB SPL*  2kHz: 62.3 ± 5.2 dB SPL*  4kHz: 52.6 ± 4.6 dB SPL*  **8kHz: 57.5 ± 4.8 dB SPL***  **10kHz: 65.1 ± 7.0 dB SPL***  **12kHz: 65.1 ± 5.5 dB SPL***  **14kHz: 69.7 ± 10.3 dB SPL***  **16kHz: 64.9 ± 7.0 dB SPL*** | I: 1kHz: 77.6 ± 4.7 dB SPL*  2kHz: 69.9 ± 0.0 dB SPL*  4kHz: 55.0 ± 5.7 dB SPL*  **8kHz: 57.7 ± 9.1 dB SPL***  **10kHz: 63.4 ± 5.6 dB SPL***  **12kHz: 72.5 ± 4.6 dB SPL***  **14kHz: 69.9 ± 10.1 dB SPL***  **16kHz: 70.3 ± 0.0 dB SPL*** | I: >0.05  >0.05  >0.05  >0.05  >0.05  >0.05  >0.05  >0.05 | -  -  -  -  -  -  -  - | NA | - |
|  |  |  |  | II: Cis + NP-MTOS-0(n=4) |  |  | 50µl of NP solution  II: NP - α-Tos, no pred |  |  |  | II: 1kHz: 72.5 ± 5.8 dB SPL*  2kHz: 65.0 ± 3.3 dB SPL*  4kHz: 59.9 ± 2.7 dB SPL*  **8kHz: 59.9 ± 8.6 dB SPL***  **10kHz: 70.0 ± 7.0 dB SPL***  **12kHz: 76.7 ± 2.5 dB SPL***  **14kHz: 66.6 ± 3.7 dB SPL***  **16kHz: 66.6 ± 3.7 dB SPL*** | II: 1kHz: 74.8 ± 10.1 dB SPL*  2kHz: 59.9 ± 8.4 dB SPL*  4kHz: 57.5 ± 3.3 dB SPL*  **8kHz: 60.1 ± 8.2 dB SPL***  **10kHz: 72.5 ± 9.6 dB SPL***  **12kHz: 73.4 ± 5.9 dB SPL***  **14kHz: 72.5 ± 9.5 dB SPL***  **16kHz: 72.3 ± 9.8 dB SPL*** | II: >0.05  >0.05  >0.05  >0.05  >0.05  >0.05  >0.05  >0.05 | -  -  -  -  -  -  -  - |  | - |
|  |  |  |  | III: Cis + NP-MVE-15(n=12) |  |  | 50µl of NP solution  III: NP – vit E, 15% pred |  |  |  | III: 1kHz: 70.0 ± 6.8 dB SPL*  2kHz: 54.2 ± 7.3 dB SPL*  4kHz: 49.8 ± 6.8 dB SPL*  **8kHz: 53.5 ± 6.9 dB SPL***  **10kHz: 52.4 ± 9.0 dB SPL***  **12kHz: 53.7 ± 13.5 dB SPL***  **14kHz: 57.1 ±7.6 dB SPL***  **16kHz: 52.0 ± 11.0 dB SPL*** | III: 1kHz: 72.6 ± 6.3 dB SPL*  2kHz: 60.0 ± 7.7 dB SPL*  4kHz: 55.4 ± 8.1 dB SPL*  **8kHz: 55.6 ± 5.2 dB SPL***  **10kHz: 61.0 ± 7.3 dB SPL***  **12kHz: 68.1 ± 8.7 dB SPL***  **14kHz: 73.2 ± 7.0 dB SPL***  **16kHz: 75.1 ± 5.1 dB SPL*** | III: >0.05  >0.05  >0.05  >0.05  **<0.05**  >0.05  >0.05  >0.05 | -  -  -  -  -  +  +  ++ |  | - |
|  |  |  |  | IV: Cis + NP-MTOS-15(n=4) |  |  | 50µl of NP solution  IV: NP - α-Tos, 15% pred |  |  |  | IV: 1kHz: 62.4 ± 4.7 dB SPL*  2kHz: 42.3 ± 5.3 dB SPL*  4kHz: 45.0 ± 5.8 dB SPL*  **8kHz: 60.0 ± 8.4 dB SPL***  **10kHz: 52.4 ± 4.8 dB SPL***  **12kHz: 64.8 ± 5.8 dB SPL***  **14kHz: 62.4 ± 5.2 dB SPL***  **16kHz: 59.9 ± 2.7 dB SPL*** | IV: 1kHz: 62.4 ± 4.7 dB SPL*  2kHz: 44.8 ± 5.9 dB SPL*  4kHz: 45.0 ± 5.7 dB SPL*  **8kHz: 62.4 ± 5.6 dB SPL***  **10kHz: 64.8 ± 5.9 dB SPL***  **12kHz: 67.3 ± 4.8 dB SPL***  **14kHz: 67.3 ± 4.8 dB SPL***  **16kHz: 62.4 ± 4.7 dB SPL*** | IV: >0.05  >0.05  >0.05  >0.05  >0.05  >0.05  **<0.05**  **<0.05** | -  -  -  -  +  -  -  - |  | - |
| **Ramaswamy 2017**  **[14]** | Pred | CBA/CAJ mice (10 weeks old) | 1/2 | I: Cis + Pred UL(n=6) | I: Cis + saline UL (n=6),  Untreated right ear (n=12) | 4mg/kg 2 cycl. 2d on - 10d off +  1 cycl. 4d on – 12d off | I: 1.8µl pred 40mg/ml | IT injection + magnetic field in NP groups, 1d before 2^nd^ + 3^rd^ cis cycle, | ABR threshold,  Hair cell loss | 47 d (after 3^rd^ cycle) | ABR:  8kHz:11.7±2.6dB*  **16kHz:17.6±6.2dB***  **32kHz:28.6±6.2 dB***  I: HCC: 33% | ABR:  8kHz:17.5±5.4dB*  **16kHz:17.6±8.4dB***  **32kHz:31.1± 7.4dB***  I: HCC: 72% | ABR:  NS  HCC:  NS | ABR:  -  -  -  HCC: HC+ | NA | - |
|  |  |  |  | II: Cis + Pred-NP UL (n=6) | II: Cis + NP UL (n=4),  Untreated right ear (n=10) |  | II: 1.8µl magnetic pred-NP suspension, 82 µg/ml |  |  |  | ABR:  8kHz:8.4±4.12 dB*  **16kHz:14.3± 3.68dB***  **32kHz:15.2± 6.3dB***  II: HCC: 9% | ABR:  8kHz:19.7±35.8dB*  **16kHz:29.0±13.08dB***  **32kHz:34.4±3.5dB***  II: HCC: 65% | ABR:  **<0.05**  **<0.05**  **<0.05**  HCC:  NS | ABR:  +  +  +  HCC: HC+ |  | - |
| **Pierstorff 2019 [15]** | Flu  (OR-102) | Young Guinea pigs | 1 | Cis + OR-102 implant (n=9) | Cis only (n=9) | 12mg/kg, 14d after implant | PVA coated Flu particles, cylindrical/hexagonal shape | RWM puncture | Click ABR threshold,  DPOAE | 1, 14 and 28 d | ABR click: Day 1: 19.4 ± 1.3 dB  Day 14: 25.6 ± 1.3 dB  Day 28: 25.0 ± 3.1 dB DPOAE: Day 1: 14.8 ± 4.5 dB  Day 14:11.1 ± 3.4 dB  Day 28:13.6 ± 4.8 dB | ABR click: Day 1: 22.8 ± 1.2 dB  Day 14: 23.9 ± 2.3 dB  Day 28: 40.7 ± 2.5 dB  DPOAE:  Day 1: 15.1 ± 4.5 dB  Day 14: 7.9 ± 5.6 dB  Day 28: 0.0 ± 4.7 dB | ABR click:  **P=0.695**  **P=0.695 <0.0001**  DPOAE:  >0.05  >0.05  >0.05 | ABR: -  -  + DPOAE:  HC-  HC-  HC- | NA | NA |
| **Zhao 2024 [16]** | EGT | C57BL/6J mice  (7 weeks) | 1 | Cis + EGT(n=10) | Cis only(n=10) | 2mg/ml IT, single dose | 500mM | IT injection | ABR threshold*,  OHC loss *% (dfa)* | 7 d,  14 d | 7d: 8kHz: 5.2dB  **16kHz: 5.5dB**  **32kHz: 20.3dB**  14d:  8kHz:9.9dB  **16kHz 14.7dB**  **32kHz: 9.7dB**  20-40% OHC loss  3.5mm: 15%  4.0mm: 26%  4.5mm: 30%  5.0mm: 34% | 7d:  8kHz:10.2dB  **16kHz:10.6dB**  **32kHz: 20.3dB**  14d:  8kHz:14.9dB  **16kHz 10.1dB**  **32kHz: 44.2dB**  50-80% OHC loss  3.5mm: 30%  4.0mm: 55%  4.5mm: 81%  5.0mm: 30% | **p< 0.05**  p>0.05  p>0.05  p>0.05  p>0.05  **p < 0.001**  **p< 0.001**  **p< 0.001**  **p< 0.001**  **p< 0.001** | 7d:  -  -  -  14d:  -  -  ++ | NA | - |
| **Paksoy 2011[17]** | Dex,  Vit E | Wistar rats | 2 | I: Cis + Dex (n=8) | Cis only (n=8) | 20mg/kg IP, single dose | I: 4 mg/kg Dex, 30min prior to cis | IT injection | ABR threshold shifts | 3 d | I: clicks: 8.64 ± 2.17 dB  4kHz: 1.60 ± 1.31 dB  **8kHz: 4.75 ± 2.40 dB**  **12kHz: 8.70 ± 3.37 dB**  **16kHz: 4.26 ± 2.06 dB** | Clicks: 39.66 ± 1.44 dB  4kHz: 7.25 ± 2.63 dB  **8kHz: 8.44 ± 1.60 dB**  **12kHz: 71.12 ± 4.20 dB**  **16kHz: 71.85 ± 5.85 dB** | **I: <0.001** | ++  -  -  ++  ++ | + | + |
|  |  |  |  | II: Cis + Vit E (n=8) |  |  | II: 4 g/kg Vit E, 30min prior to cis |  |  |  | II: clicks: 8.64 ± 2.17 dB  4kHz: 3.30 ± 1.44 dB  **8kHz: 7.20 ± 2.09 dB**  **12kHz: 10.75 ± 1.99 dB**  **16kHz: 13.30 ± 3.14 dB** |  | **II: <0.001** | ++  -  -  ++  ++ | + | + |
| **Özel 2016[18]** | Dex,  Pred | Wistar Hannover rats (15–17 months) | 2 | I: Cis + Dex (n=2) | Cis + NaCl (n=2) | 8 mg/kg IP, single dose | I: 4mg/ml Dex, day 0, 3, 6 and 9 | IT injection | DPOAE | 10 d | I: 996Hz: 8.7 dB SPL*  1416Hz: 12.5 dB SPL*  2001Hz: 10.5 dB SPL*  2832Hz: 11.6 dB SPL*  4003Hz: 8.5 dB SPL*  4755Hz: 12.4 dB SPL*  5654Hz: 16.6 dB SPL*  6728Hz: 23.0 dB SPL*  7998Hz:26.9 dB SPL* | 996Hz: 17.8 dB SPL*  1416Hz: 20.8 dB SPL*  2001Hz: 21.3 dB SPL*  2832Hz: 22.8 dB SPL*  4003Hz: 26.3 dB SPL*  4755Hz: 29.2 dB SPL*  5654Hz: 30.0 dB SPL*  6728Hz: 35.7 dB SPL*  7998Hz: 46.2 dB SPL* | **I: <0.05** | HC+ HC+  HC+  HC+  HC+  HC+  HC+  HC+ HC+ | NA | NA |
|  |  |  |  | II: Cis + Pred (n=2) |  |  | II: 40mg/ml Pred, day 0, 3, 6 and 9 |  |  |  | II: 996Hz: 9.7 dB SPL*  1416Hz: 12.5 dB SPL*  2001Hz: 12.5 dB SPL*  2832Hz: 13.0 dB SPL*  4003Hz: 12.7 dB SPL*  4755Hz: 16.6 dB SPL*  5654Hz: 17.4 dB SPL*  6728Hz: 21.0 dB SPL*  7998Hz: 26.9 dB SPL* |  | **II: <0.05** | HC+  HC+  HC+  HC+  HC+  HC+  HC+  HC+  HC+ | NA | NA |
| **Martín-Saldaña 2017**  **[19]** | Dex,  α-Toc | Albino Wistar rats | 1 | I: Cis + NP-MVE-15Dx (n=6) | I: Cis + PBS (n=6) | 10 mg/kg IP, single dose | I: Dx-loaded NP + MVE polymer | Bullostomy | ASSR | 3 d | I: **8kHz: 3.9 ± 17.1 dB SPL***  **12kHz: 15.6 ± 15.1 dB SPL***  **16kHz: 13.7 ± 11.4 dB SPL***  **20kHz: 16.4 ± 8.0 dB SPL***  **24kHz: 13.7 ± 15.1 dB SPL***  **28kHz: 11.4 ± 10.2 dB SPL***  **32kHz: 7.7 ± 21.6 dB SPL*** | I: **8kHz: 19.8 ± 17.4 dB SPL***  **12kHz: 32.3 ± 9.4 dB SPL***  **16kHz: 35.7 ± 9.0 dB SPL***  **20kHz: 31.9 ± 9.8 dB SPL***  **24kHz: 31.5 ± 14.5 dB SPL***  **28kHz: 29.6 ± 10.2 dB SPL***  **32kHz: 46.3 ± 9.8 dB SPL*** | **I: < 0.05** | +  +  ++  +  +  +  ++ | NA | - |
|  |  |  |  | II: Cis + NP-MTOS-15Dx (n=6) | II: Cis + PBS (n=6) |  | II: Dx-loaded NP + MTOS polymer |  |  |  | II: **8kHz: 9.2 ± 7.6 dB SPL***  **12kHz: 25.5 ± 16.7 dB SPL***  **16kHz: 25.5 ± 16.0 dB SPL***  **20kHz: 33.4 ± 15.1 dB SPL***  **24kHz: 31.9 ± 12.9 dB SPL***  **28kHz: 30.4 ± 12.5 dB SPL***  **32kHz: 34.5 ± 9.0 dB SPL*** | II: **8kHz: 14.1 ± 11.7 dB SPL***  **12kHz: 23.6 ± 14.7 dB SPL***  **16kHz: 28.5 ± 18.5 dB SPL***  **20kHz: 35.3 ± 26.6 dB SPL***  **24kHz: 34.9 ± 15.9 dB SPL***  **28kHz: 31.9 ± 10.5 dB SPL***  **32kHz: 34.2 ± 12.5 dB SPL*** | II: NS | -  -  -  -  -  -  - |  | - |
|  |  |  |  | III: Cis + NP-MVE-10a-TOS (n=6) | III: Cis + PBS (n=6) |  | III: α-TOS-loaded NP + MVE polymer |  |  |  | III: **8kHz: 6.4 ± 13.5 dB SPL***  **12kHz: 8.4 ± 13.3 dB SPL***  **16kHz: 4.9 ± 11.1 dB SPL***  **20kHz: 11.7 ± 16.1 dB SPL***  **24kHz: 16.9 ± 17.3 dB SPL***  **28kHz: 21.7 ± 15.8 dB SPL***  **32kHz: 20.1 ± 16.9 dB SPL*** | III: **8kHz: 7.6 ± 3.9 dB SPL***  **12kHz: 12.4 ± 9.4 dB SPL***  **16kHz: 17.9 ± 19.6 dB SPL***  **20kHz: 27.1 ± 12.2 dB SPL***  **24kHz: 25.9 ± 8.3 dB SPL***  **28kHz: 23.2 ± 13.4 dB SPL***  **32kHz: 26.4 ± 18.9 dB SPL*** | III: NS | -  -  +  +  -  -  - |  | - |
|  |  |  |  | IV: Cis + NP-MTOS-10a-TOS (n=6) | IV: Cis + PBS (n=6) |  | IV: α-TOS-loaded NP + MTOS polymer |  |  |  | IV: **8kHz: 8.5 ± 7.9 dB SPL***  **12kHz: 20.0 ± 7.9 dB SPL***  **16kHz: 29.9 ± 9.9 dB SPL***  **20kHz: 31.5 ± 11.5 dB SPL***  **24kHz: 32.0 ± 9.9 dB SPL***  **28kHz: 35.2 ± 8.7 dB SPL***  **32kHz: 17.9 ± 9.4 dB SPL*** | IV: **8kHz: 16.4 ± 10.6 dB SPL***  **12kHz: 36.5 ± 5.9 dB SPL***  **16kHz: 35.0 ± 10.6 dB SPL***  **20kHz: 63.0 ± 11.8 dB SPL***  **24kHz: 45.0 ± 10.2 dB SPL***  **28kHz: 36.8 ± 6.2 dB SPL***  **32kHz: 54.1 ± 7.1 dB SPL*** | **IV: <0.05** | +  +  -  ++  +  -  ++ |  | - |
| **Simsek 2019[20]** | Dex,  Res | Albino Wistar Rats (3 months) | 2 | I: Cis + Dex (n=8) | I: Cis only (n=8) | 15mg/kg IP, single dose | I: 0.05ml Dex 4mg/ml | IT injection | Click ABR threshold  Mean(range)  ,  DPOAE | 72 h | I: Click ABR  55 (50–60) dB  DPOAE:  2832kHz: 16.48±2.03 dB  4004kHz: 18.88±2.96 dB  5652kHz: 21.33±2.40 dB | I: Click ABR  70 (60–85) dB  DPOAE:  2832kHz: 11.60 ± 1.19 dB  4004kHz: 11.80 ± 0.99 dB  5652kHz: 13.29 ± 1.62 dB | **I: <0.001** | I: ABR:  +  DPOAE:  HC+  HC+  HC+ | NA | NA |
|  |  |  |  | II: Cis + Res (n=8) | II: Cis + NaCl (n=8) |  | II: 0.05ml Res 20mg/ml |  |  |  | II: Click ABR  52 (50–60) dB  DPOAE:  2832kHz: 15.30 ± 2.421 dB  4004kHz: 17.73 ± 2.57 dB  5652kHz: 18.83 ± 1.97 dB | II: Click ABR  72 (65–80) dB  DPOAE:  2832kHz: 11.96 ± 1.02 dB  4004kHz: 11.37 ± 2.11 dB  5652kHz: 14.01 ± 3.43 dB | **II: <0.001** | II: ABR:  +  DPOAE:  HC+  HC+  HC+ |  | NA |
|  |  |  |  |  | III: Cis + DMSO (n=8) |  |  |  |  |  |  | III: Click ABR  70 (60–85) dB  DPOAE:  2832kHz: 11.60 ± 1.66 dB  4004kHz: 12.60 ± 1.08 dB  5652kHz: 12.04 ± 1.28 dB |  |  |  | NA |
| **Taş 2021[21]** | Dex,  Pred | Albino Wistar Rats  (3 months) | 2 | I: Cis + Dex (n=8) | I: Cis only (n=8) | 15mg/kg IP, single dose | I: 0.05ml Dex 4mg/ml | IT injection | Click ABR threshold  Mean(range)  ,  DPOAE | 72 h | I: Click ABR  55 (50-60) dB  DPOAE:  2832kHz: 16.48 ± 2.03 dB  4004kHz: 18.88 ± 2.96 dB 5652kHz: 21.33 ± 2.40 dB | I: Click ABR  70 (60-85) dB  DPOAE:  2832kHz: 11.60 ± 1.19 dB  4004kHz: 11.80 ± 0.99 dB  5652kHz: 13.29 ± 1.62 dB | ABR:  P > 0.5  DPOAE:  **<0.001** | II: ABR:  +  DPOAE:  HC+  HC+  HC+ |  | NA |
|  |  |  |  | II: Cis + Pred (n=8) | II: Cis + NaCl (n=8) |  | II: 0.05ml Pred 62.5 mg/ml |  |  |  | II: Click ABR  60 (55-70) dB  DPOAE:  2832kHz: 13.81 ± 1.09 dB  4004kHz: 15.99 ± 1.82 dB  5652kHz: 18.60 ± 1.398 dB | II: Click ABR  72 (65-80) dB  DPOAE:  2832kHz: 11.96 ± 1.02 dB  4004kHz: 11.37 ± 2.11 dB  5652kHz: 14.01 ± 3.43 dB | ABR:  P > 0.5  DPOAE:  **<0.001** | II: ABR:  +  DPOAE:  HC+  HC+  HC+ |  | NA |
| **Yi 2024**  **[22]** | CUR,  TSA | Guinea pigs | 2 | CS@CUR NPs, (n=3) | Cis only (n=3) | 12mg/kg IP,  single dose | CUR: 1.5 μg/kg,  TSA: 3 μg/kg | IT injection, postauricular incision, 1h before Cis | Pure tone and click ABR threshold shifts,  OHC survival rate | 3d | ABR:  **4kHz: 31.6 dB***  **8kHz: 38.3 dB***  Click: 38.2 dB*  OHC:  Basal: 70%  Middle:77%  Apical:89% | ABR:  **4kHz: 63.5 dB***  **8kHz:65.1 dB***  Click:65.2 dB*  OHC:  Basal:33%  Middle:49%  Apical:78% | ABR:  p<0.01 | ABR:  ++  ++  ++  OHC:  + | NA | + |
|  |  |  |  | CS@TSA NPs, (n=3) |  |  |  |  |  |  | ABR:  **4kHz: 35.0 dB***  **8kHz:36.7 dB***  Click:38.3 dB*  OHC:  Basal: 66%  Middle:78%  Apical:85% |  | ABR:  p<0.01 | ABR:  ++  ++  ++  OHC:  + |  | + |
|  |  |  |  | CS@CUR/TSA NPs, (n=3) |  |  |  |  |  |  | ABR:  **4kHz: 16.5 dB***  **8kHz:21.5 dB***  Click:21.5 dB*  OHC:  Basal:84%  Middle:91%  Apical:95% |  | ABR:  p<0.01 | ABR:  ++  ++  ++  OHC:  + |  | + |
|  |  |  |  | TTMR-CS@CUR NPs, (n=3) |  |  |  |  |  |  | ABR:  **4kHz: 18.1 dB***  **8kHz:18.1 dB***  Click:20 dB*  OHC:  Basal:82%  Middle:85%  Apical:95% |  | ABR:  p<0.01 | ABR:  ++  ++  ++  OHC:  + |  | + |
|  |  |  |  | TTMR-CS@TSA NPs, (n=3) |  |  |  |  |  |  | ABR:  **4kHz: 21.5 dB***  **8kHz:18.2 dB***  Click:18.2 dB*  OHC:  Basal:80%  Middle:88%  Apical:92% |  | ABR:  p<0.01 | ABR:  ++  ++  ++  OHC:  + |  | + |
|  |  |  |  | TTMR-CS@CUR/TSA NPs, (n=3) |  |  |  |  |  |  | ABR:  **4kHz: 8.1 dB***  **8kHz:11.5 dB***  Click:8.2 dB*  OHC:  Basal:96%  Middle:97%  Apical:98% |  | ABR:  p<0.01 | ABR:  ++  ++  ++  OHC:  + |  | + |
| *HC+/-: hair cell protection+/damage-, ABR: auditory brainstem response; α-Toc: α-tocopheryl; BL: bilateral; Cis: cisplatin; Dex: dexamethasone; DMSO: dimethylsulfoxide; DPOAE: distortion product otoacoustic emission; Flu: flucticasone; EGT: ergothioneine; IT: intratympanic; IP: intraperitoneal; NA: not applicable; NaCl: sodium chloride 0.9%; NP: nano-particle; PBS: phosphate buffered saline; Pred: methylprednisolone; Res: resveratrol; UL: unilateral; vit E: vitamin E; HCC: Hair cell count, AuNR@DEX-MS-saponin, NPs/DEX: gold nanorod@dexamethasone-mesoporous silica-saponin, CUR: curcumin , TSA: tanshinone, TTMR-CS:tube type microrobot – chitosan nanoparticle ,CS NP: chitosan nano particle, Vitrogel-Lmp/Dexa: Dexa-loaded lipid/pectin/BSA microparticles, dfa: distance from apex*  **Results derived from figure using WebPlotDigitizer*  *Design: 1. Ear vs ear; 2. Subject vs subject* | | | | | | | | | | | | | | | | |

Supplementary Table 2B. Preclinical studies on the efficacy and safety of locally administered chemical deactivator otoprotectives.

| **Chemical deactivators** | | | | | | | | | | | **End point Result** | | | **Conclusion** | | |
| --- | --- | --- | --- | --- | --- | --- | --- | --- | --- | --- | --- | --- | --- | --- | --- | --- |
| **Author, Year** | **Intervention drug** | **Model**  **(age)** | **Design** | **Study arm, treatment (No. subjects)** | **Comparison arm, treatment (No. subjects)** | **Cisplatin dose, frequency** | **Intervention Drug Specification** | **Intervention Drug administration** | **Evaluation Method** | **FU time**  **post-Tx** | **Hearing function outcomes** | | **p-value** | **Efficacy** | **safety** | **≥20dB** |
|  |  |  |  |  |  |  |  |  |  |  | **With intervention drug** | **Without intervention drug** |  |  |  |  |
| **Mohan 2014**  **[23]** | L-NAC | Young Guinea pigs | 2 | I: Cis + L-NAC NC in gel (n=5) | Cis only (n=5) | 5.5 mg/kg/d IP, 3d | I: L-NAC NC in gel solution | 2h prior to Cis,  IT injection | ABR threshold shift | 3 d | I: 2kHz: 64.7 ± 5.77 dB HL  **4kHz: 92.5 ± 0.00 dB HL**  **8kHz: 74.0 ± 0.00 dB HL**  **16kHz: 73.8 ± 21.26 dB HL**  20kHz: 63.9 ± 8.75 dB HL | 2kHz: 64.9 ± 11.47 dB HL  **4kHz: 77.4 ± 8.11 dB HL**  **8kHz: 67.9 ± 12.60 dB HL**  **16kHz: 78.6 ± 16.53 dB HL**  20kHz: 76.3 ± 10.33 dB HL | >0.05 | **-**  **-**  **-**  **-**  **+** | NA | - |
|  |  |  |  | II: Cis +L-NAC 2% in gel (n=5) |  |  | II: L-NAC 2% in gel solution |  |  |  | II: 2kHz: 81.3 ± 8.78 dB HL  **4kHz: 84.2 ± 2.89 dB HL**  **8kHz: 84.8 ± 14.65 dB HL**  **16kHz: 81.3 ± 17.02 dB HL**  20kHz: 79.0 ± 13.92 dB HL |  |  | **-**  **-**  **-**  **-**  **+** |  | - |
| **Chen 2022**  **[24]** | NAC | adult male Hartley albino Guinea pigs | 2 | I: Cis + NAC 1% (n=16) | Cis + saline (n=16) | 10 mg/kg IP | I: NAC 1% | 3d prior to Cis  0.2–0.3 ml,  IT injection | ABR threshold shift | 4 w | I: click: 76 ± 10 dB*  1kHz: 83 ± 11 dB*  2kHz: 80 ± 16 dB*  **4kHz: 70 ± 6 dB***  **8kHz: 74 ± 22 dB*** | Click: 89 ± 5 dB*  1kHz: 91 ± 5 dB*  2kHz: 98 ± 11dB*  **4kHz: 85 ± 12 dB***  **8kHz:81 ± 11dB*** | I: >0.05 | -  +  +  - | NA | - |
|  |  |  |  | II: Cis + NAC 2% (n=16) |  |  | II: NAC 2% |  |  |  | II: click: 30 ± 5 dB*  1kHz: 31 ± 9 dB*  2kHz: 24 ± 3 dB*  **4kHz: 33 ± 3 dB***  **8kHz: 37 ± 5 dB*** |  | **II: <0.05** | ++  ++  ++  ++ |  | + |
|  |  |  |  | III: Cis + NAC 4% (n=16) |  |  | III: NAC 4% |  |  |  | III: click: 104 ± 15 dB*  1kHz: 114 ± 20 dB*  2kHz: 117 ± 7 dB*  **4kHz: 103 ± 2 dB***  **8kHz: 99 ± 17 dB*** |  | III: >0.05 | -  -  -  - |  | - |
| **Choe 2004**  **[25]** | NAC,  Lac | Hartley Guinea pigs | 2 | I: Cis + NAC (n=12) | I: Cis only (n=10) | 10mg/kg/d IP, 2d | I: 2% NAC diluted in saline | IT injection | DPOAE | 4 d | I: 2kHz: -7.5 dB*  **4kHz: -1.0 dB***  **8kHz: -0.6 dB***  **16kHz: -4.2 dB*** | I: 2kHz: -6.5 dB*  **4kHz: -9.1 dB***  **8kHz: -11.6 dB***  **16kHz: -15.3 dB*** | **I: <0.05** | HC-  HC-  HC+  HC+ | NA | NA |
|  |  |  |  | II: Cis + Lac (n=12) | II: Cis + saline (n=12) |  | II: Lac in Ringer’s |  |  |  | II: 2kHz: -0.9 dB*  **4kHz: 3.3 dB***  **8kHz: 7.2 dB***  **16kHz: -0.6 dB*** | II: 2kHz: -9.9 dB*  **4kHz: -6.5 dB***  **8kHz: -5.4 dB***  **16kHz: -11.3 dB*** | **II: <0.005** | HC-  HC-  HC+  HC+ |  | NA |
| **Nader 2010**  **[26]** | NAC,  Lac | Hartley Guinea pigs | 1 | I: Cis + NAC (n=7) | I: Cis + saline (n=7) | 3mg/kg/w IP, 8w | I: 20% NAC 200mg/ml | 30 min prior to Cis  IT injection | ABR mean threshold | 3w + 8w | I: 3w: 81.5 ± 8.4 dB  8w: 79.6 ± 8.2 dB  2kHz: 82 dB*  **4kHz: 81 dB***  **6kHz: 81 dB***  **8kHz: 89 dB*** | 3w: 17.7 ± 4.7 dB  8w: 40.8 ± 6.2 dB 2kHz: 25 dB  **4kHz: 32 dB**  **6kHz: 29 dB**  **8kHz: 27.5 dB** | >0.05 | -  -  -  - | NAC: - | NA |
|  |  |  |  | II: Cis + Lac (n=9) | II: Cis + saline (n=9) |  | II: Lac in Ringer’s |  |  |  | II: 3w: 23.7 ± 6 dB  8w: 34.5 ± 6.1 dB 2kHz: 7.5 dB  **4kHz: 18 dB**  **6kHz: 20 dB**  **8kHz: 23dB** |  | **<0.05** | +  +  -  - | Lac: + | NA |
| **Saliba 2010 [27]** | NAC, Pred | Hartley Guinea pigs | 1 | I: Cis + NAC (n=4) | I: Cis only (n=4) | 3mg/kg/w IP, 5w | I: 4% NAC | 30 min prior to Cis  IT injection | ABR threshold shift | 6 w | 1kHz: 25dB*  2kHz: 21dB*  3kHz: 18dB*  **4kHz: 13dB***  **6kHz: 12dB***  **8kHz: 8dB*** | 1kHz: 15dB*  2kHz: 16dB*  3kHz: 20dB*  **4kHz: 12dB***  **6kHz: 4dB***  **8kHz: 1dB*** | >0.05 | -  -  -  -  -  - | NA | - |
|  |  |  |  | II: Cis + Pred (n=3) | II: Cis only (n=3) |  | II: 62.5 mg/mL Pred |  |  |  | 1kHz: 45 dB*  2kHz: 44 dB*  3kHz: 44 dB*  **4kHz:54 dB***  **6kHz:59 dB***  **8kHz:48 dB*** | 1kHz: 24 dB*  2kHz: 20 dB*  3kHz: 20 dB*  **4kHz: 20 dB***  **6kHz:14 dB***  **8kHz: 13 dB*** | NAC vs Pred: **0.02** | -  -  -  -  -  - |  | - |
| **Li 2001**  **[28]** | L-met | Fisher 334 rats (7-weeks) | 2 | Cis + L-met (n=11) | Cis only (n=12) | 5mg/kg IP, 3x every 72h | L-met 25mg/ml, continuous from day -2 until 10 | Osmotic minipump RWN | ABR threshold shift  SEM (OHC loss) | 10 d | ABR:  1kHz: 0.7 dB SPL*  2kHz: 0.6 dB SPL*  4kHz: 0.6 dB SPL*  **8kHz: 1.3 dB SPL***  **16kHz: 2.7 dB SPL***  **18kHz: 0.6 dB SPL***  OHC loss  Basal: <2±2.72%  Middle: 2.86±3.65% | ABR:  1kHz: -0.7dB SPL*  2kHz: -1.5dB SPL*  4kHz: -0.8dB SPL*  **8kHz: 2 dB SPL***  **16kHz: 12 dB SPL***  **18kHz: 10 dB SPL***  OHC loss  Basal: 56.67±26.31%  Middle: 13.33±8.81% | **<0.05**  **<0.001**  **<0.05** | ABR:  -  -  -  -  -  - OHC loss | NA | - |
| **Korver 2002**  **[29]** | D-met | Adult  Chinchillas | 1 | Cis + D-met (n=13) | Cis only (n=13) | 6 µL of 0.66 mg/mL, topical on RWM | 5 µL of 25 mg/mL | Bullostomy, topical on RWM | ABR threshold shift,  SEM (OHC loss) | 7 d | ABR  Click: 6 ± 11 dB*  **8kHz: 10 ± 12 dB***  16kHz: 14 ± 14 dB*  OHC loss  Basal: 2%  Middle: 5%  Apical: 6% | ABR  Click: 67 ± 10 dB*  **8kHz: 71 ± 5 dB***  16kHz: 52 ± 11 dB*  OHC loss  Basal: 82%  Middle: 55%  Apical: 42% | **10^-13^**  **10^-11^**  **10^-8^**  NA NA  NA | ABR  ++  ++ OHC loss | NA | + |
| **Wimmer 2004**  **[30]** | D-met  BDNF  FGF-2 | pigmented female Guinea pigs | 2 | I: Cis + Dmet (n=6) | Cis + saline (n=6) | 3mg/kg/d IP, 5d | I: D-met 40 mg/mL | Mini-osmotic pump in bulla | OAE threshold | 6 d | 2kHz: 66.6 dB SPL*  **4kHz: no emission**  **5kHz: no emission** | 2kHz: 72.5 dB SPL*  **4kHz: no emission**  **5kHz: no emission** | >0.05 | D-met: +- | NA | NA |
|  |  |  |  | II: Cis + B-DNF (n=6) |  |  | II: BDNF 7.5 µg/ml |  |  |  | 2kHz: 67.2 dB SPL*  **4kHz: no emission**  **5kHz: no emission** |  | >0.05 | BDNF: - |  | NA |
|  |  |  |  | III: Cis + FGF-2 (n=6) |  |  | III: FGF-2 7.5 0g/ml |  |  |  | 2kHz: 67.9 dB SPL*  **4kHz: no emission**  **5kHz: no emission** |  | >0.05 | FGF-2: - |  | NA |
| **Ekborn 2003**  **[31]** | TU | pigmented  Guinea pigs | 2 | Cis + TU (n=10) | Cis +AP (n=10) | 8mg/kg IP, single dose | 27 mg/ml TU dissolved in AP, UL | Osmotic pump into scala tympani | ABR threshold shift,  OHC | 5 d | **4kHz: 1±1 dB**  **12kHz: 14±9.9 dB**  **16kHz: 22±13 dB**  OHC  loss sign. lower | **4kHz: 14±5.7 dB**  **12kHz: 35±10 dB**  **16kHz: 33±9.4 dB**  OHC  loss sign. higher | >0.09  **<0.05** | +  ++  + | +/- | - |
|  |  |  |  |  |  |  |  |  |  |  |  |  |  | OHC |  |  |
| **Wang 2004**  **[32]** | Cas-3 inh | Guinea pigs | 2 | I: Cis + cas-3 inh (n=6) | Cis only (n= 21) | 10mg/kg IP, single dose | I: 83-mmol/L cas-3 in AP, 100µmol/L | Osmotic minipump in basal cochlea | CAP | 3 d | I: 2kHz: -2.0 dB*  **4kHz: -0.6 dB***  **6kHz: -0.1 dB***  **8kHz: -4.8 dB***  **10kHz: -0.3 dB***  **12kHz: -0.5 dB***  **16kHz: -4.6 dB***  20kHz: -14.3 dB*  26kHz: -19.2 dB* | 2kHz: -19.5 dB*  **4kHz: -21.5 dB***  **6kHz: -29.6 dB***  **8kHz: -33.7 dB***  **10kHz: -34.8 dB***  **12kHz: -36.9 dB***  **16kHz: -42.4 dB***  20kHz: -52.3 dB*  26kHz: -60.5 dB* | **NA** | **+**  **++**  **++**  **++**  **++**  **++**  **++**  **++**  **++** | NA | + |
|  | Cas-8 inh |  |  | II: Cis + cas-8 inh (n=5) |  |  | II: 85-mmol/L cas-8 in AP, 100µmol/L |  |  |  | II: 2kHz: -14.9 dB*  **4kHz: -18.7 dB***  **6kHz: -16.7 dB***  **8kHz: -21.1 dB***  **10kHz: -28.6 dB***  **12kHz: -30.9 dB***  **16kHz: -48.4 dB***  20kHz: -48.4 dB*  26kHz: -40.6 dB* |  |  | -  -  +  +  -  -  -  -  ++ |  | - |
|  | Cas-9 inh |  |  | III: Cis + cas-9 inh (n=5) |  |  | III: 81-mmol/L cas-9 in AP, 100µmol/L |  |  |  | III: 2kHz: 3.0 dB*  **4kHz: 6.5 dB***  **6kHz: 4.8 dB***  **8kHz: -3.2 dB***  **10kHz: -6.6 dB***  **12kHz: -8.7 dB***  **16kHz: -13.2 dB***  20kHz: -11.0 dB*  26kHz: -10.0 dB* |  |  | +  +  ++  ++  ++  ++  ++  ++  ++ |  | + |
|  | Cat-B inh |  |  | IV: Cis + cat-B (n=5) |  |  | IV: 72-mmol/L cat-B in AP, 100µmol/L |  |  |  | IV: 2kHz: -25.0 dB*  **4kHz: -30.0 dB***  **6kHz: -25.7 dB***  **8kHz: -28.6 dB***  **10kHz: -38.5 dB***  **12kHz: -38.0 dB***  **16kHz: -44.4 dB***  20kHz: -43.5 dB*  26kHz: -44.0 dB* |  |  | -  -  -  -  -  -  -  -  ++ |  | - |
| **Tanaka 2004**  **[33]** | pH manipulation | male rats | 1/2 | I: Cis + pH 6.0 (n=6) | I: Cis + pH 7.4 (n=6) | 13mg/kg IP, single dose | I: acidic PBS (pH 6.0) | Bullostomy, RWN application | ABR threshold changes, | 3 d | I: Click: 5.0 ± 5.0 dB  2kHz: 6.7 ± 4.9 dB  4kHz: 3.3 ± 2.1 dB  **8kHz: 5.0 ± 3.4 dB**  **16kHz: 13.3 ± 5.6 dB**  **32kHz: 15.0 ± 6 dB** | I: Click: 18.3 ± 3.1 dB  2kHz: 20.0 ± 5.8 dB  4kHz: 16.7 ± 4.9 dB  **8kHz: 18.3 ± 6.5 dB**  **16kHz: 31.7 ± 6.0 dB**  **32kHz: 36.7 ± 8.4 dB** | **<0.05**  **<0.05**  **<0.05**  **<0.05**  **<0.01**  **<0.01** | +  +  +  +  ++ | NA | - |
|  |  |  |  | II: Cis + pH 9.0 (n=6) | II: Cis + pH 7.4 (n=6) |  | II: basic PBS (pH 9.0) |  |  |  | II: Click: 28.3 ± 4.8 dB  2kHz: 30.0 ± 3.7 dB 4kHz: 25.0 ± 3.4 dB  **8kHz: 30.0 ± 2.6 dB**  **16kHz: 48.3 ± 7.5 dB**  **32kHz: 50.0 ± 6 dB** | II: Click: 20.0 ± 4.5 dB  2kHz: 25.0 ± 4.3 dB  4kHz: 18.3 ± 1.7 dB **8kHz: 26.7 ± 2.1 dB**  **16kHz: 31.7 ± 5.4 dB**  **32kHz: 45.0 ± 6 dB** | **<0.05**  >0.05  **<0.05**  >0.05  **<0.05**  >0.05 | -  -  -  -  - |  | - |
| **More 2010**  **[34]** | CPS | FVB and C57BL/6 wild-type Mice (3–4 weeks) | 2 | I: Cis + CPS (30min) (n=5) | Cis + H_2_O (n=5) | 20mg/kg IP, single dose | 0.025 mg/kg CPS  I: injected 30min before cis | IT injection | ABR threshold shift | 72 h | I: Click: 9.75 ± 1.77 dB  8kHz: 4.88 ± 0.91 dB  **16kHz: 1.50 ± 1.25 dB**  **32kHz: 21.38 ± 3.71 dB** | Click: 36.1 ± 5.23 dB  8kHz: 17.9 ± 4.29 dB  **16kHz: 14.4 ± 5.16 dB**  **32kHz: 33.9 ±1.72 dB** | **<0.0001**  **<0.01**  **<0.05**  **<0.05** | +  +  + | NA | - |
|  |  |  |  | II: Cis + CPS (4h) (n=5) |  |  | 0.025 mg/kg CPS  II: injected 4 h before cis |  |  |  | II: Click: 11.4 ± 1.94 dB  8kHz: 5.11 ± 1.84 dB  **16kHz: NS**  **32kHz: NS** |  | **<0.0001**  **<0.01**  >0.05  >0.05 | + |  | - |
| **Saliba 2012 [35]** | Erd | Hartley Guinea pigs | 1 | I: Cis + Erd1 (n=10) | I: Cis + saline (n=10) | 3mg/kg/w IP, 8w | Erd1: 1.125 mg/cc | 45 min prior to Cis,  IT injection | ABR mean threshold | 9 w | 1kHz:43.5 dB*  2kHz:39.5 dB*  4kHz44 dB*  **6kHz:39 dB***  **8kHz:34 dB*** | 1kHz:25.5 dB*  2kHz:27.5 dB*  4kHz:35 dB*  **6kHz:31 dB***  **8kHz:30 dB*** | Saline vs Erd <0.03 | Erd1  -  -  -  -  - | **-** | - |
|  |  |  |  |  |  |  | Erd2: 2.25 mg/cc |  |  |  | 1kHz:46.5 dB*  2kHz:47.5 dB*  4kHz51.5 dB*  **6kHz:33 dB***  **8kHz:41 dB*** | 1kHz:56 dB*  2kHz: 51.5 dB*  4kHz: 58.5 dB*  **6kHz: 39.5 dB***  **8kHz:42.5 dB*** |  | Erd2  -  -  -  -  - |  | - |
|  |  |  |  | II: Cis + Erd2 (n=10) | II: Cis + Erd4 (n=10) |  | Erd4: 4.5 mg/cc |  |  |  |  |  |  | Erd4  -  -  -  -  - |  | - |
| **Spankovich 2016**  **[36]** | Therm | young cava albino Guinea pigs | 2 | I: Cis + cool (n=7) | Cis + euthermic (n=4) | 12mg/kg IP, single dose | Cool: 30°C | 2h prior to cis,  Ext. ear canal | ABR threshold shift,  OHC count | 3 d | I: **4kHz: 0.667 ± 12.4 dB**  **8kHz: 4.667 ± 13.0 dB**  **16kHz: 7.33 ± 15.4 dB**  24kHz: 3.33 ± 11.56 dB  I: OHC count: 348 ± 8.68 | **4kHz: 3.33 ± 13.8 dB**  **8kHz: 5.83 ± 13.6 dB**  **16kHz: 34.16 ± 16.0 dB**  24kHz: 30.83 ± 12.1 dB  OHC count: 234 ± 65.01 | **I: <0.05** | Cool:  -  -  ++  ++ | NA | + |
|  |  |  |  | II: Cis + warm (n=5) |  |  | Euthermic: 37°C |  |  |  | II: **4kHz: 34.67 ± 12.4 dB**  **8kHz: 47.33 ± 13.0 dB**  **16kHz: 42.67 ± 15.4 dB** 24kHz: 27.33 ± 1.56 dB  II: OHC count: 187 ± 41.40 |  | II: >0.05 | Euthermic:  -  -  -  - |  | - |
|  |  |  |  |  |  |  | Warm: 44°C |  |  |  |  |  |  | Warm: - |  | - |
| **Stanford 2021[37]** | Therm | Cava albino Guinea pigs  (8-10weeks) | 2 | I: Cis + cool water (n= 9) | Cis only (n= 6) | 4mg/kg/w IP, 3w | I: Cool water 22°C | Ext. ear canal | ABR threshold shift.  DPOAE,  OHC count | 1 m | ABR differences:  *Cis only vs water:*  **8kHz: 19.1 ± 6.5 dB**  **12kHz: 33.6 ± 8.6 dB**  20kHz: 41.3 ± 8.6 dB  24kHz: 34.1 ± 7.3 dB  32kHz: 25.9 ± 8.5 dB  *Cis only vs ear bar:*  **8kHz: 14.8 ± 6.5 dB**  **12kHz: 28.0 ± 8.6 dB**  20kHz: 33.75 ± 8.6 dB  24kHz: 35.4 ± 7.3 dB  32kHz: 29.6 ± 8.5 dB  OHC count:  I: <40% loss  II: <40% loss | DPOAE differences:  *Cis only vs water:*  **8kHz: 28.0 ± 8.6 dB**  **12kHz: 24.0 ± 7.1 dB**  20kHz: 23.1 ± 6.5 dB  24khz: 25.1 ± 6.1 dB  32kHz: 13.3 ± 7.4 dB  *Cis only vs ear bar:*  **8kHz: 21.12 dB**  **12kHz: 16.5 ± 7.1 dB**  20kHz: 23.1 ± 6.5 dB  24kHz: 22.6 ± 6.1 dB  32kHz: 11.4 ± 7.4 dB  OHC count:  Cis only: 80% loss | **I: <0.05**  **II: <0.05** | Water:  +  ++  ++  ++  ++ | NA | + |
|  |  |  |  | II: Cis + cool bar (n= 9) |  |  | II: Cool ear bar 15°C |  |  |  |  |  |  | Ear bar:  +  ++  ++  ++  ++ |  | + |
| **Roldan-Fidalgo 2014 [38]** | DMSO | Female Wistar rats | 1 | Cis + DMSO (n=7) | Cis + saline (n=7) | 10mg/kg IP, single dose | Prior to Cis  0.5 % DMSO (0.03 ml) | IT injection | ASSR | 5 d | **8kHz: 45.9 ± 9.0dB SPL***  **12kHz: 53.7 ± 14.0dB SPL***  **16kHz: 53.7 ± 16.1dB SPL***  **20kHz: 42.0 ± 15.6dB SPL***  **24kHz: 62.0 ± 13.7dB SPL***  **28kHz: 52.8 ± 12.0dB SPL***  **32kHz: 53.9 ± 15.3dB SPL*** | **8kHz: 52.2 ± 7.6dB SPL***  **12kHz: 59.9 ± 16.7dB SPL***  **16kHz: 59.9 ± 12.1dB SPL***  **20kHz: 45.6 ± 11.3dB SPL***  **24kHz: 62.1 ± 9.0dB SPL***  **28kHz: 61.9 ± 9.1dB SPL***  **32kHz: 53.0 ± 11.7dB SPL*** | >0.05 | -  -  -  -  -  -  - | NA | - |
| *ABR: auditory brainstem response; AP: artificial perilymph; ASSR: Auditory Steady State Response; BDNF: Brain-derived neurotrophic factor; BL: bilateral; CAP: compound action potential; cas: caspase; cat: cathepsin; Cis: cisplatin; CPS: copper sulfate; D-met: D-methionine; DMSO: dimethylsulfoxide; DPOAE: distortion product otoacoustic emission; Erd: erdosteine; FGF-2: Fibroblast growth factor-2; inh: inhibitor; IT: intratympanic; IP: intraperitoneal; Lac: lactate; L-met: L-methionine; L-NAC: L-N-acetylcysteine; NA: not applicable; NAC: N-acetylcysteine; NS: not significant; OHC: outer hair cell; RWM: round window membrane; RWN: round window niche; SEM: Scanning Electron Microscopy; Therm: thermal treatment; TU: thiourea; UL: unilateral*  **Results derived from figure using WebPlotDigitizer Design: 1. Ear vs ear; 2. Subject vs subject* | | | | | | | | | | | | | | | | |

Supplementary Table 2C. Preclinical studies on the efficacy and safety of locally administered calcium channel blocker otoprotectives.

| **Calcium channel blockers** | | | | | | | | | | | **End point Result** | | | **Conclusion** | | |
| --- | --- | --- | --- | --- | --- | --- | --- | --- | --- | --- | --- | --- | --- | --- | --- | --- |
| **Author, Year** | **Intervention drug** | **Model (age)** | **Design** | **Study arm, treatment (No. subjects)** | **Comparison arm, treatment (No. subjects)** | **Cisplatin dose, frequency** | **Intervention Drug Specification** | **Intervention Drug administration** | **Evaluation Method** | **FU time**  **post-Tx** | **Hearing function outcomes** | | **p-value** | **Efficacy** | **Safety** | **≥20dB** |
|  |  |  |  |  |  |  |  |  |  |  | **With intervention drug** | **Without intervention drug** |  |  |  |  |
| **Naples 2016**  **[39]** | Dil | CBA/J mice (4-week) | 1 | I: Cis + Dil 2mg/kg UL (n=9) | I: Cis + saline UL (n=9) | 14mg/kg IP, single dose | I: Dil 2mg/kg, UL injection | All daily for 5 days after cis,  IT injection | I: Tone evoked ABR, % change from baseline | 7,14,21d | I: Day 7:  8kHz: 9.2 ± 13.0%*  **16kHz: 7.2 ± 18.1%***  **24kHz: -4.2 ± 7.3%***  **32kHz: 2.2 ± 11.9%***  no significant difference at day 14 and 21 | I: Day 7:  8kHz: 13.8 ± 13.1%*  **16kHz: 18.2 ± 11.1%***  **24kHz: 5.1 ± 9.7%***  **32kHz: 4.6 ± 10.9%***  no significant difference at day 14 and 21 | I: **0.038**  0.051 | -  +  -  - | NA | - |
|  |  |  | 2 | II: Cis + Dil 2mg/kg BL (n=7) | II: Cis + saline BL (n=8) |  | II: Dil 2mg/kg BL injection |  | II,III: Click evoked ABR, % change from baseline |  | II: Day 7: 13.6 ± 10.7%*  Day 14: 16.3 ± 17.5%*  Day 21: 11.9 ± 24.5%* | II: Day 7: 48.8 ± 25.8%  Day 14: 35.9 ± 35.6%  Day 21: 30.2 ± 45.1%* | II: **0.001** | 2: + |  |  |
|  |  |  | 2 | III: Cis + Dil 4mg/kg BL (n=8) | III: Cis + saline BL (n=7) |  | III: Dil 4mg/kg BL injection |  |  |  | III: Day 7: 14.2 ± 47.4%*  Day 14: 13.6 ± 44.2%*  Day21: 9.5 ± 43.1%* |  | III: **0.011** | 3: + |  |  |
| **Naples 2018 [40]** | Dil | Hartley Guinea pigs | 2 | Cis + Dil (n=10) | Cis + saline (n=10) | 8mg/kg IP, single dose | Dil 2mg/kg, 5 consecutive days | IT injection | Click ABR threshold shift | 14 d | Day 1: 2.5 ± 6.4 dB*  Day 2: 4.5 ± 10 dB*  Day 3: 12.5 ± 9.4 dB*  Day 7: 2.5 ± 9.3 dB*  Day 14: 5.1 ± 3 dB* | Day 1: -1.2 ± 2.5 dB*  Day 2: 3.8 ± 4.3 dB*  Day 3: 6.3 ± 2.8 dB*  Day 7: 11.3 ± 4 dB*  Day 14: 11.3 ± 3.6 dB* | NA | + | NA | NA |
| **Naples 2021 [41]** | Dil | CBA/J mice (4 weeks) | 2 | Cis + Dil UL (n=9) | Cis + saline UL (n=13) | 14mg/kg IP, single dose | 5µl IT CGP hydrogel 2mg/kg Dil | IT injection | ABR threshold shift | 7 d | Click: 7.7±1.08dB SPL  4kHz: 0.56±2.29 dB  8kHz: 3.89±1.24 dB  **16kHz: 7.22±1.31 dB**  **24kHz: 7.78±2.2 dB**  **32kHz: 10±3.8 dB** | Click: 16.5±1.00 dB SPL  4kHz: 9.6±1.84 dB  8kHz: 17.3±1.93 dB  **16kHz: 16.5±1.67 dB**  **24kHz: 28.0±4.97 dB**  **32kHz: 31.9±4.82 dB** | **<0.001**  **0.009 <0.001 0.001**  **0.006**  **0.005** | -  +  -  ++  ++ | NA | + |
| **Güven 2024**  **[42]** | Mem | young-adult Hartley guinea pigs | 2 | Cis + Mem (n=8) | Cis + Phys serum (n=8) | 12mg/kg IP, single dose | 0.2ml of 2 mg in hydrochloride sol. | IT injection 30 min before Cis | ABR threshold shift,  DPOAE | 3d | **ABR before:** **8kHz:15.71±5.1 dB**  **12kHz:15±5.1 dB at 12kHz, 16kHz:13.75±5 dB** 24kHz:14.38±5.1 dB  32kHz: 17.5±4.4 dB  **ABR after:**  **8kHz:67.86±9.2 dB 12kHz:77.86±4.2 dB 16kHz:77.14±4.6 dB** 24kHz:77.86±5.7 dB  32kHz: 77.86±4.2  **DPOAE before:**  1kHz:10±8.45 dB  1.5kHz :14.19±7.65 dB  2kHz :17.5±10.75 dB  3kHz:16.56±8.8 dB  **4kHz**:**14±9.2 dB at 4kHz, 6kHz: 17.38±9.9 dB at, 8kHz:32.81±9.2 dB at, 12kHz: 36.75±7.9 dB at 16kHz:27.56±3.3 dB at 16kHz (HF): 30±10.5 dB at** 24kHz: 35.13±5.2 dB,  32 kHz :26.18±8.4 dB  **DPOAE after:**  1kHz:0±6.3 dB  1.5kHz :2.21±5.29 dB  2kHz :4.86±11.1 dB  3kHz: 2±13.7 dB  **4kHz: −2.21±7.55 dB**  **6kHz: −0.86±8.96 dB**  **8kHz: −1.79±4.22 dB**  **12kHz: 6.21±12.9 dB**  **16kHz:18.43±9.8 dB**  **16kHz (HF): 3.5±5.3 dB**  24kHz: 7.29±14.5 dB  32 kHz: 6.71±6.3 dB | **ABR before:**  **8kHz:19.38±2.5 dB at 8kHz, 12kHz:18.75±3.4 dB at 16kHz:14.38±5.1 dB at** 24kHz:16.25±5 dB  32kHz: 16.25±5 dB  **ABR after:**  **8kHz:66.25±12.5 dB**  **12kHz: 67.5±14.8 dB 16kHz:63.12±20.5 dB** 24kHz:73.75±12.5 dB 32kHz: 70±16.3 dB  **DPOAE before:**  1kHz:3.56±6.96 dB  1.5kHz :8.06±10 dB  2kHz :13.69±8.37 dB  3kHz:13.25±9.49 dB  **4kHz**:**8.87±11.5 dB**  **6kHz: 11.81±11.5 dB**  **8kHz:14.25±15.3 dB**  **12kHz: 32.69±7.78 dB**  **16kHz:27±2.7 dB**  **16kHz (HF): 15.8±10 dB** 24kHz:31.31±5.5 dB  32 kHz:24.1±7.31 dB  **DPOAE after:**  1kHz: −4.44±11.52 dB  1.5kHz: −0.63±8.37 dB  2kHz :1.75±8.38 dB  3kHz:8.44±8 dB  **4kHz:4.06±9.9 dB**  **6kHz: 4.25±11.2 dB**  **8kHz:6.88±15.6 dB**  **12kHz: 21.19±8.7 dB**  **16kHz:23.31±6 dB**  **16kHz (HF): 8±8.1 dB**  24kHz: 24.13±11.6 dB  32 kHz: 12.5±10.71 dB | ABR:  **p<0.05**  **p<0.05**  **p<0.05**  **p<0.05**  **p<0.05**  DPOAE:  p>0.05  p>0.05  p>0.05  **p<0.05**  p>0.05  **p<0.05**  **p<0.05**  **p<0.05**  **p<0.05**  p>0.05  **p<0.05**  **p<0.05** | ABR:  -  -  -  -  -  DPOAE:  HC-  HC -  HC -  HC -  HC -  HC -  HC -  HC -  HC -  HC -  HC +  HC - | NA | - |
| **Zhou 2025 [43]** | L-arg,  KN93, GH@PDA@LK | SD Rats (7-weeks) | 2 | Cis + L-Arg (n=7) | Cis only (n=7) | 4.6mg/kg IP, 3 d | 50mg/kg | IT injection, 1 day before Cis | ABR, * OHC  Survival%* | 2 d | **8kHz: 39.3dB SPL ***  **16kHz: 47.7dB SPL ***  **24kHz: 57.4dB SPL ***  **32kHz: 56.8dB SPL***  40kHz: 68.2dB SPL*****  Apex; 95%  Middle: 42%  Base: 20% | **8kHz: 45.0 ± 7.9 dB SPL 16kHz: 48.0 ± 13.0 dB SPL 24kHz: 57.0 ± 7.5 dB SPL 32kHz: 58.0 ± 10.36 dB SPL** 40kHz: 67.0 ± 9.08 dB SPL  Apex; 95%  Middle: 44%  Base: 23% | NA,  p>0.05 | -  -  -  -  - | NA | + |
|  |  |  |  | Cis + KN93 (n=7) | Saline (no Cis) (n=7) |  | 10mg/kg |  |  |  | **8kHz: 37.1dB SPL***  **16kHz: 43.3dB SPL***  **24kHz: 53.5dB SPL***  **32kHz: 61.4dB SPL***  40kHz: 63.0dB SPL*****  Apex: 95%  Middle: 45%  Base: 22% |  | NA, p>0.05 | -  -  -  -  - |  |  |
|  |  |  |  | Cis + L-Arg + KN93 (n=7) | Saline (no Cis) (n=7) |  | L-arg 50mg/kg +  KN93 10mg/kg |  |  |  | **8kHz: 35.5dB SPL***  **16kHz: 46.6dB SPL***  **24kHz: 53.8dB SPL***  **32kHz: 57.0dB SPL***  40kHz: 63.9dB SPL*****  Apex; 96%  Middle: 43%  Base: 23% |  | NA,  p>0.05 | -  -  -  -  - |  |  |
|  |  |  |  | GH@PDA@LK (n=7) | GH@PDA (n=7) |  | L-Arg 50mg/kg + KN93 10mg/kg |  |  | 2 d | **8kHz: 18.3dB SPL***  **16kHz: 20.3dB SPL***  **24kHz: 29.3B SPL***  **32kHz: 38.3dB SPL***  40kHz: 41.4dB SPL*****  Apex; 95%  Middle: 74%  Base: 48% |  | NA,  **p<0.001** | ++  ++  ++  +  ++ |  |  |
|  |  |  |  |  |  |  |  |  |  | 32d | **8kHz: 39.4dB SPL***  **16kHz: 45.1dB SPL***  **24kHz: 53.4dB SPL ***  **32kHz: 55.9dB SPL***  40kHz: 59.3dB SPL*  Apex; 95%  Middle: 74%  Base: 48% | **8kHz: 62.5dB SPL ***  **16kHz: 72.2dB SPL ***  **24kHz: 77.1dB SPL ***  **32kHz: 78.3dB SPL** *****  40kHz: 78.5dB SPL*****  Apex; 84%  Middle: 61%  Base: 36% | NA,  **p<0.001** | ++  ++  ++  ++  + |  |  |
| *Design: 1. Ear vs ear; 2. Subject vs subject ; ABR: auditory brainstem response; BL: bilateral; CGP: chitosan-glycerophosphate; Mem: Memantine; L-arg: L-arginine, KN93: calmodulin-dependent kinase II inhibitor, GH@PDA@: methacrylate-gelatin microsphere (GH) conjugate polydopamine (PDA) layer, Cis: cisplatin; Dil: diltiazem; IT: intratympanic; IP: intraperitoneal; NA: not applicable; UL: unilateral.*  **Results derived from figure using WebPlotDigitizer;* | | | | | | | | | | | | | | | | |

Supplementary Table 2D. Preclinical studies on the efficacy and safety of locally administered biological otoprotectives.

| ***Biologicals*** | | | | | | | | | | | **End point Result** | | | **Conclusion** | | |
| --- | --- | --- | --- | --- | --- | --- | --- | --- | --- | --- | --- | --- | --- | --- | --- | --- |
| **Author Year** | **Intervention drug** | **Model**  **(age)** | **Design** | **Study arm, treatment (No. subjects)** | **Comparison arm, treatment (No. subjects)** | **Cisplatin dose, frequency** | **Intervention Drug Specification** | **Intervention Drug administration** | **Evaluation Method** | **FU time**  **post-Tx** | **Hearing function outcomes** | | **p-value** | **Efficacy** | **Safety** | **≥20dB** |
|  |  |  |  |  |  |  |  |  |  |  | **with intervention drug** | **without intervention drug** |  |  |  |  |
| **Mukherjea 2010**  **[44]** | siRNA | male Wistar rats | 2 | Cis + NOX3 siRNA | Cis + scramble siRNA | 11mg/kg IP over 30 min | NOX3 siRNA  0.3µg, 0.6µg, 0.9µg | 48h prior to Cis,  IT injection | ABR shift, OHC damage% | 3-5 d | ABR: 23 ± 5 dB  OHC damage:  5% | ABR: day 3: 35 ± 5 dB  day 5: 49 ± 5 dB  OHC damage:  55% | **<0.05** | ++  ++  ++  ++  ++ | NA | NA |
| **Kaur 2011**  **[45]** | siRNA | male Wistar rats | 2 | Cis + STAT1 siRNA | Cis + scramble siRNA | 11mg/kg IP | STAT 1 siRNA 0.9µg | 48h prior to Cis,  IT injection | ABR shift | 72h | **8kHz: 11 ± 2 dB**  **16kHz: 12 ± 3 dB**  **32kHz: 15 ± 3 dB** | **8kHz: 25 ± 3 dB**  **16kHZ: 35 ± 5 dB**  **32kHz: 42 ± 4 dB** | **<0.05**  (n=6) | +  ++  ++ | NA | + |
| **Ghosh 2018**  **[46]** | siRNA | male Wistar rats  (5–6 weeks) | 2 | Cis + CB2R siRNA | Cis only | 11mg/kg i.p. | CB2R siRNA (JWH015) 50 µl of 2.5 nmol in PBS | IT injection | ABR shift | 72h | **8kHz: 0.6 ± 0.6 dB**  **16kHz: 1.9 ± 1 dB**  **32kHz: 5.6 ± 1.5 dB** | **8kHz: 5 ± 1.5 dB**  **16kHz: 15 ± 2.5 dB**  **32kHz: 27 ± 1.7 dB** | **<0.05**  (n=12) | -  +  ++ | NA | - |
| **Sheehan 2018**  **[47]** | siRNA | male Wistar rats | 2 | Cis + R-PIA | Cis only | 11mg/kg i.p. | 50 µl R-PIA solution 1 µM | IT injection | ABR shift | 72h | **8kHZ: 0 dB***  **16kHz: 0 dB***  **32kHz: 2.7 ± 1.1 dB*** | **8kHz: 5.2 ± 1.8 dB***  **16kHz: 8.6 ± 2.5 dB***  **32kHz: 30.2 ± 2.3 dB*** | **<0.05** (n=5) | -  -  ++ | NA | - |
| **Al Aameri 2023**  **[48]** | siRNA | male Wistar rats | 2 | I: Cis + SB225002 (n=4-6) | I: Cis (n=4-6)  II: Cis (n=4-6) | 11mg/kg IP, single dose | 50 µl of 1.4 nmol SB225002 | IT injection | ABR shift | 72h | I: **8kHZ: 6.0 ± 3.2 dB**  **16kHz: 3.3 ± 1.8 dB**  **32kHz: 10.0 ± 4.8 dB** | I: **8kHz: 17.5 ± 4.1 dB**  **16kHz: 22.5 ± 5.3 dB**  **32kHz: 32.5 ± 5.3 dB** | **<0.0001** | +  +  ++ | NA | - |
|  |  |  |  | II: Cis + CXCR2 siRNA (n=4-6) |  |  | 50 µl; 0.9 µg CXCR2 siRNA |  |  |  | II: **8kHz: 3.3 ± 2.1 dB**  **16kHz: 7.5 ± 3.5 dB**  **32kHz: 20.0 ± 3.0 dB** | II: **8kHz: 11.8 ± 3.1 dB**  **16kHz: 24.3 ± 4.3 dB**  **32kHz: 40.6 ± 3.5 dB** | **<0.0001** (n=4) | -  +  ++ |  |  |
| **Cooper 2006**  **[49]** | viral delivery | Adult male Sprague-Dawley rats | 1 | I: Cis + AAV.XIAP (n=4) | I: Cis only (n=4) | 16 mg/kg IP  in lactated Ringer’s solution over 30 min | I: 2-5 µl XIAP solution | 2 m prior to Cis, RWM injection | ABR shifts,  OHC count per cross section | 72h | I: 4kHz: 14.9 ± 7.8 dB SPL*  **8kHz: 4.6 ± 8.3 dB SPL***  **16kHz: 12.1 ± 5.4 dB SPL***  **32kHz: 6.2 ± 5.7 dB SPL***  OHC: 1.98 ± 0.42 | I: 4kHz: 7.5 ± 5.1 dB SPL*  **8kHz: 7.6 ± 2.7 dB SPL***  **16kHz: 32.9 ± 3.3 dB SPL***  **32kHz: 27.0 ± 5.8 dB SPL***  OHC: 1.08 ± 0.58 | >0.05  >0.05  **<0.05**  **<0.05**  **<0.002** | -  -  ++  ++ | NA | + |
|  |  |  |  | II: Cis + AAV.GFP (n=5) | II: Cis only (n=5) |  | II: 2-5 µl GFP solution |  |  |  | II: 4kHz: 36.0 ± 7.7 dB SPL*  **8kHz: 48.2 ± 9.3 dB SPL***  **16kHz: 38.6 ±10.2 dB SPL***  **32kHz: 28.7 ± 4.0 dB SPL***  OHC: 0.66±0.34 | II: 4kHz: 31.3 ± 7.3dBSPL*  **8kHz: 37.3 ± 6.9 dB SPL***  **16kHz: 48.2 ± 7.9 dB SPL***  **32kHz: 38.5 ± 5.1 dB SPL***  OHC: 0.94 ±0.24 | GFP NS  XIAP vs. GFP **<0.05** | -  -  -  - |  |  |
| **Chan 2007**  **[50]** | viral delivery | male Sprague-Dawley rats | 1 | Cis + AAV.d-XIAP (N=7) | Cis + AAV.YFP (n=7) | 12 mg/kg IP in lactated Ringer’s solution  over 3d | 3 µl viral solution | 2 w prior to Cis  RWM injection | ABR shifts | 2w | I: 4kHz: 1 dB SPL*  **8kHz: 6.1dB SPL***  **16kHz: 7.7 dB SPL***  **32kHz: 12.6 dB SPL*** | 4kHz: 9.8 dB SPL*  **8kHz: 13.2 dB SPL***  **16kHz: 32.4 dB SPL***  **32kHz: 40.8 dB SPL*** | >0.05  >0.05  **<0.05**  **<0.05** | -  -  ++  ++ | NA | + |
|  |  |  |  | Cis + AAV.d-XIAP-d (N=8) |  |  |  |  |  |  | II: 4kHz: 8.1 dB SPL*  **8kHz: 7.9 dB SPL***  **16kHz: 26.8 dB SPL***  **32kHz: 13 dB SPL*** |  | >0.05  >0.05  >0.05  **<0.05** | -  -  -  ++ |  |  |
|  |  |  |  | Cis + AAV.d-XIAP-t (N=7) |  |  |  |  |  |  | III:4kHz: 5.1 dB SPL*  **8kHz: 7.4 dB SPL***  **16kHz: 26.7 dB SPL***  **32kHz: 23.9 dB SPL*** |  | >0.05  >0.05  >0.05  >0.05 | -  -  -  + |  |  |
| **Jie 2015 [51]** | viral delivery | albino guinea pigs (8-12 weeks) | 1 | Cis + AAV-6-  myc-XIAP (n=7) | Cis + saline (n=7) | 4mg/kg/d, max 7d | 10 µl of viral vector | 2 w prior to Cis  RWM application | ABR shifts  OHC loss | 7d | Average:32.85 ± 1.44 dB SPL over 4-16kHz  1kHz:10 dB SPL*  2kHz: 5.9 dB SPL*  **4kHz:8.2 dB SPL***  **8kHz: 4 dB SPL***  **16kHz: 11 dB SPL***  32kHz: 31 dB SPL*  899.9 ± 170/cochlea | Average: 54.76 ± 4.87 dB over 4-16kHz  1kHz: 17.5 dB SPL*  2kHz: 13.1 dB SPL*  **4kHz: 15.3 dB SPL***  **8kHz: 19.8 dB SPL***  **16kHz: 25.5 dB SPL***  32kHz: 44.1 dB SPL*  1040.7 ± 171/cochlea | **0.0046**  **<0.001** | -  -  -  +  +  + | NA | - |
| **Kuang 1999 [52]** | GDNF | Albino Guinea pigs | 1 | I: Cis + GDNF (n=4) | I: Cis only (n=4) | 1 mg/kg/d, 15 d  7.5 mg/kg, 2x, 5d interval | I: 1 mg/ml | I: IT injection | OHC loss | 28 d | I: 25 ± 4% | I: 42 ± 4% | **<0.05** | + | NA | NA |
|  |  |  |  | II: Cis + GDNF (n=3) | II: Cis only (n=3) |  | II: 50 ng/ml at 0.5 µl/h | II: Alzet minipump, inner ear |  |  | II: 21 ± 4% | II: 46 ± 3% | **<0.05** |  |  |  |
| **Meen 2010 [53]** | BDNF | Guinea pigs | 1 | Cis + BDNF (n=11) | Cis + saline (n=11) | 12 mg/kg IP, single dose | 0.5 µg of BDNF  1m after cis | Cochleostomy 1m after cis | ABR threshold | 1 + 2 m | 1m: 58.5 ± 12 dB SPL  2m: 58.1 ± 10.9 dB SPL | 1m: 50 ± 9.8 dB SPL  2m: 67.5 ± 10.1 dB SPL | 0.629 | - | NA | NA |
|  |  |  |  |  | cochleostomy only (n = 4) |  | 0.5 µg of saline  1m after cis |  |  |  |  | 1m: 83.2 ± 5.3 dB SPL  2m: 80.0 ± 20 dB SPL |  |  |  |  |
| **Pisani 2025 [54]** | rhBDNF | Male Wistar rats (4-6 weeks) | 1 | Cis + rhBDNF (n=5) | Cis+ vehicle (N=28) | 12 mg/kg IP, single dose | 5 μg/μl suspended in a thermogel vehicle solution | IT injection 1h after Cis | ABR threshold *, OHC  Survival* | 3d | **6kHz:8.9 dB**  **12kHz:6.9 dB**  **16kHz: 4.9 dB**  **20kHz:5.9 dB**  **24kHz:2.9 dB**  **32kHz:4.9 dB**  Apex: 97%  Middle: 90%  Base: 91% | **6kHz:14.9 dB**  **12kHz:15.9 dB**  **16kHz: 18.9 dB**  **20kHz:20.9 dB**  **24kHz:18.9 dB**  **32kHz:21.9 dB**  Apex: 96%  Middle: 70%  Base: 68% | p>0.05  p>0.05  **p< 0.05**  **p< 0.05**  **p< 0.05**  **p< 0.05**  p>0.05  **p< 0.05**  **p< 0.05** | -  -  +  +  +  + | NA | - |
|  |  |  |  | Cis + rhBDNF (n=23) |  |  |  |  |  | 7d | **6kHz:8.7 dB**  **12kHz:8.6 dB**  **16kHz: 9.5 dB**  **20kHz:10.6 dB**  **24kHz:8.5 dB**  **32kHz:9.5 dB** | **6kHz:17.3 dB**  **12kHz:18.6 dB**  **16kHz: 22.5 dB**  **20kHz:25.8 dB**  **24kHz:25.6 dB**  **32kHz:26.1 dB** | **p< 0.05 p< 0.05 p< 0.05 p< 0.05 p< 0.05 p< 0.05** | -  +  +  +  +  + |  |  |
| **Yurtsever 2020 [55]** | PRP | female rats (16-32weeks) | 2 | Cis + PRP (n=4) | Cis + saline (n=4) | 10mg/d for 2d | 0.1-0.3 ml | IT injection | ABR shifts | 4d + 3w | day 4: 45±5.34 dB  ,  week 3: 39.37±4.17 dB | 49.37±3.20 dB  ,  44.37±2.84 dB | 0.083  **0.038** | + | NA | NA |
| **Tsai 2021 [56]** | UCMSC | C57BL/6 Mice (8 weeks) | 2 | Cis + UCMSC (n=6) | Cis only(n=14) | 4 mg/kg/d, 5d | 10 μL, 1.2 μg/μL UCMSC exosome | 3d after Cis,  RWM injection | ABR | 7 d | **12kHz: 21.67 ± 4.01 dB SPL** | **12 kHz: 46.43 ± 3.57 dB SPL** | **>0.05** | ++ | NA | - |
| *AAV: adeno-associated viral vector; ABR: auditory brainstem response; BDNF: brain-derived nerve growth factor; cis: cisplatin; rhBDNF: recombinant human brain-derived neurotrophic factor; GDNF: glial cell line-derived neurotrophic factor; GFP: green fluorescent protein; IP: intraperitoneal; PBS: phosphate buffered saline; PRP: Platelet-rich plasma; RNA: ribonucleic acid; R-PIA: (R)-N-phenyl isopropyl adenosine; RWM: round window membrane; siRNA: small interfering RNA; XIAP: X-linked inhibitor of apoptosis protein*  *Design: 1. Ear vs ear; 2. Subject vs subject* | | | | | | | | | | | | | | | | |

Supplementary Table 2E. Preclinical studies on the efficacy and safety of locally administered miscellaneous otoprotectives.

| **miscellaneous category** | | | | | | | | | | | **End point Result** | | | **Conclusion** | | |
| --- | --- | --- | --- | --- | --- | --- | --- | --- | --- | --- | --- | --- | --- | --- | --- | --- |
| **Author, Year** | **Intervention drug** | **Model (age)** | **Design** | **Study arm, treatment (No. subjects)** | **Comparison arm, treatment (No. subjects)** | **Cisplatin dose, frequency** | **Intervention Drug Specification** | **Intervention Drug administration** | **Evaluation Method** | **FU time**  **post-Tx** | **Hearing function outcomes** | | **p-value** | **Efficacy** | **Safety** | **≥20dB** |
|  |  |  |  |  |  |  |  |  |  |  | **With intervention drug** | **Without intervention drug** |  |  |  |  |
| **Whitworth 2004 [57]** | Adenosine agonists | adult Chinchillas | 2 | I: Cis + R-PIA (n=6) | Cis + PBS (n=6) | 2µl of 0.66mg/ml cis on RWM | I: 10µl R-PIA (1 mM) in PBS | Bullostomy, RWM application | ABR | 72 h | **1kHz: 9 ± 2.3 dB**  **2kHz: 12 ± 2.9 dB 4kHz: 11.7 ± 2.0 dB**  **8kHz: 24 ± 6.7 dB**  16kHz: 39 ± 7.2 dB | **1kHz: 35 ± 8.0 dB**  **2kHz: 45.7 ± 7.8 dB**  **4kHz: 46.5 ± 8.7 dB**  **8kHz: 57.1 ± 8.0 dB**  16kHz: 49.2 ± 5.6 dB | **<0.01**  **<0.001**  **<0.01**  **<0.01**  >0.05 | ++  ++  ++  ++  + | NA | + |
|  |  |  |  | II: Cis + CPPA (n=6) |  |  | II: 10µl CCPA (100 mM) in PBS |  |  |  | **1kHz: 8 ± 1.4 dB**  **2kHz: 16 ± 3.6 dB**  **4kHz: 14 ± 1.8 dB**  **8kHz: 20 ± 5.5 dB**  16kHz: 24 ± 7.3 dB |  | **<0.01**  **<0.01**  **<0.01**  **<0.05**  >0.05 | ++  ++  ++  ++  ++ |  |  |
| **Lee 2010 [58]** | EC | Sprague–Dawley rats | 1 | Cis + EC (n=8) | Cis + saline (n=8) | 14mg/kg IP, single dose | 2 mM EC daily, for 5 d | IT injection | ABR (click) threshold shift | 14 d | 31.5 ± 4.8 dB | 56.8 ± 3.9 dB | **<0.01** | + | NA | NA |
| **Celebi 2013 [59]** | Vit C | Adult female Albino rats | 2 | Cis + Vit C (n=6) | Cis + saline (n=5) | 16mg/kg IP, single dose | 100 mg/mL vit C | 30 min prior to cis  IT injection | DPOAE | 3 d | 2kHz: -9.9 ± 3.9 dB SPL  2.8kHz: -5.1 ± 0.9 dB SPL  4kHz: -1.3 ± 3.1 dB SPL  **6kHz: -1.9 ± 3.0 dB SPL**  **8kHz: -7.4 ± 3.2 dB SPL** | 2kHz: -14.3 ± 1.1 dB SPL  2.8kHz: -6.9 ± 3.3 dB SPL  4kHz: -3.8 ± 1.2 dB SPL  **6kHz: -0.6 ± 1.4 dB SPL**  **8kHz: -9.8 ± 1.5 dB SPL** | **<0.05** | + | + | NA |
| **Shin 2013 [60]** | KR-22332 | Female Sprague–Dawley rats | 1 | Cis + KR-22332 (n=8) | Cis only (n=8) | 14 mg/kg IP, single dose | 2 mM KR-22332, daily for 5 d | IT injection | ABR (click) threshold shift | 14 d | 38.5 ± 11.3 dB | 67.3 ± 9.5 dB | **<0.01** | + | NA | NA |
| **Ozkul 2014**  **[61]** | α-lip | Adult male Guinea pigs | 2 | Cis + α-lip (n=6) | Cis only (n=6) | 12mg/kg IP, single dose | 0.2 mL of 25-mg/mL α-lip | IT injection | DPOAE SNR | 72 h | 1kHz: 3.6 dB  2kHz: 5.8 dB  3kHz: 10 dB  **4kHz: 8.3 dB**  **5kHz: 8 dB**  **6kHz: 13.6 dB**  **7kHz: 16.4 dB**  **8kHz: 19.1 dB** | 1kHz: -11 dB  2kHz: -16.1 dB  3kHz: -13.6 dB  **4kHz: -14.6 dB**  **5kHz: -18 dB**  **6kHz: -20.1 dB**  **7kHz: -23.4 dB**  **8kHz: -26 dB** | **<0.05** | HC- | NA | NA |
| **Chen 2025**  **[62]** | α-lip | Mice (7-8 weeks) | 2 | Cis + PDA@microcarriers-ALA (n=5) | IT puncture (n=5) | NA,  3 d | ≈10 µL of 1 mM PDA@microcarriers | IT injection 1d before Cis | ABR threshold shift *, OHC No.* | 10 d | 4kHz:3.2 dB  8kHz:2.3 dB  **12kHz: -4.1 dB**  **16kHz: 0 dB**  **24kHz: -6.5 dB**  **32kHz: -12 dB**  OHC No.:  Apex; 129  Middle: 122  Base: 119 | 4kHz:23.4dB  8kHz:31.9dB  **12kHz:23.6dB**  **16kHz:22.2dB**  **24kHz:24.2dB**  **32kHz:25.2dB**  OHC No.:  Apex: 102  Middle: 85  Base: 64 | ABR:  NA  OHC:  **p<0.01**  **p<0.0001**  **p<0.0001** | ++  ++  ++  ++  ++ | NA | + |
|  |  |  |  |  | Cis only (n=5) |  |  |  |  |  |  |  |  |  |  |  |
| **Demir 2015 [63]** | Melatonin | Wistar albino rats (12 weeks) | 2 | Cis + melatonin (n=8) | Cis + saline (n=8) | 12mg/kg IP, single dose | 30 min after Cis 0.1 mg/mL for 5 days | 30 min after Cis IT injection | DPOAE threshold shift | 10 d | DPOAE:  3825Hz: 2.1 dB  4549Hz: 1 dB  5434Hz: 2.4 dB  **6367Hz: 1 dB**  **7604Hz: 1.1 dB**  **9071Hz: 1 dB** | DPOAE:  3825Hz: 10 dB  4549Hz: 17 dB  5434Hz: 11 dB  **6367Hz: 13 dB**  **7604Hz: 11.4 dB**  **9071Hz: 15 dB** | **<0.05** |  | NA | - |
|  |  |  |  |  |  |  |  |  | ABR threshold shift |  | ABR:  Click: 3 dB  4kHz: 0 dB  **6kHz: 4 dB**  **8kHz: 7 dB** | ABR:  Click: 21 dB  4kHz: 21 dB  **6kHz: 20 dB**  **8kHz: 25 dB** | **<0.05** | ++  +  + |  |  |
| **Roldán-Fidalgo 2016**  **[64]** | Lutein | Wistar rats | 1 | Cis + lutein (n=8) | Cis + saline (n=8) | 10 mg/kg IP, single dose | 0.03 mL lutein 1 mg/mL | IT injection | ASSR | 5 d | **8kHz: 48.3 ± 12.8 dB SPL***  **12kHz: 53.6 ± 7.5 dB SPL***  **16kHz: 51.7 ± 8.8 dB SPL***  **20kHz: 46.7 ±14.5 dB SPL***  **24kHz: 60.0 ±13.4 dB SPL***  **28kHz: 56.8 ±12.8 dB SPL***  **32kHz: 53.5 ± 8.6 dB SPL*** | **8kHz: 51.9 ± 8.3 dB SPL***  **12kHz: 65.3 ± 5.7 dB SPL***  **16kHz: 60.3 ± 6.7 dB SPL***  **20kHz: 57.0 ±12.6 dB SPL***  **24kHz: 63.7 ±10.9 dB SPL***  **28kHz: 63.7 ± 8.5 dB SPL***  **32kHz: 60.3 ± 6.7 dB SPL*** | >0.05 | -  +  -  +  -  -  - | + | - |
| **Bekmez 2016**  **[65]** | Oxytocin | Wistar albino rats | 2 | Cis + oxytocin (n=6) | Cis + saline (n=6) | 10 mg/kg/d IP, 2d | 0.1-0.3 ml 5 IU/5 ml oxytocin, for 4 d | IT injection | DPOAE | 5 d | 2.7kHz: 0.87 dB SPL  3.2kHz: 8.37 dB SPL  3.8kHz: 3.7 dB SPL  4.5kHz: 8.22 dB SPL  5.4kHz: 6.94 dB SPL  **6.3kHz: 8.6 dB SPL**  **7.6kHz: 10.81 dB SPL** | 2.7kHz: 1.62 dB SPL 3.2kHz: 2.35 dB SPL  3.8kHz: −0.54 dB SPL  4.5kHz: 3.02 dB SPL  5.4kHz: 5.08 dB SPL  **6.3kHz: −0.93 dB SPL**  **7.6kHz: 5.50 dB SPL** | >0.05  **<0.05**  **<0.05**  **<0.05**  **<0.05**  **<0.05**  **<0.05** | + | NA | NA |
| **Teitz 2018 [66]** | Ken | Adult Wistar rats | 2 | Cis + ken (n=6) | Cis + DMSO (n=5) | 13 mg/kg IP, single dose | 310 μM ken in 0.5% DMSO | 1 h prior to cis  IT injection | ABR | 4 d | 4kHz: -2.4 ± 4.0 dB SPL*  **8kHz: -1.8 ± 3.8 dB SPL***  **16kHz: 6.1 ± 6.8 dB SPL***  **22kHz: 2.5 ± 7.3 dB SPL*** | 4kHz: 26.0 ± 8.8 dB SPL*  **8kHz: 39.9 ± 11.0 dB SPL***  **16kHz: 42.2 ± 7.2 dB SPL***  **22kHz:11.10±11.3 dB SPL*** | **<0.05**  **<0.01**  **<0.01**  **<0.05** | ++  ++  ++  - | + | + |
| **Hazlitt 2018**  **[67]** | AT7519 | FVB breeding Mice | 2 | I: Cis + AT7519 (n=8) | I: Cis + PBS/0.5% DMSO (n=7) | 30mg/kg IP, single dose | I: 5 μL AT7519 in PBS + 0.5% DMSO | 1 h prior to cis IT injection, | ABR | 15 d | I: **8kHz: 0.7 ± 3.6 dB**  **16kHz: 0.7 ± 2.1 dB**  **32kHz: 7.7 ± 2.3 dB** | I: **8kHz: 3.6 ± 2.4 dB**  **16kHz: 10.8 ± 2.2 dB**  **32kHz: 18.7 ± 4.1 dB** | >0.05  **0.0075**  **0.0288** | -  +  + | + | - |
|  | AZD5438 |  |  | II: Cis + AZD5438 (n=7) | II: Cis + PBS/0.5% DMSO (n=6) |  | II: 5 μL AZD5438 in PBS + 0.5% DMSO |  |  |  | II: **8kHz: 0.7 ± 1.9 dB**  **16kHz: 0.7 ± 1.7 dB**  **32kHz: 4.2 ± 4.5 dB** | II: **8kHz: 5 ± 1.8 dB**  **16kHz: 8.3 ± 4.1 dB**  **32kHz: 17.5 ± 2.8 dB** | >0.05  >0.05  **0.002** | -  -  + | + |  |
| **Tanyeli 2019 [68]** | FA | Male Wistar albino rats | 2 | Cis + FA (N=6) | Cis + saline (n=6) | 10mg/kg/d IP, 2d | FA 0.15 mL/day, 4d | 1 d prior to cis  IT injection | ABR | 8 d | 4kHz: 13.3 ± 13.6 dB  **8kHz: 13.3 ± 13.6 dB**  **12kHz: 15.0 ± 8.3 dB**  **16kHz: 18.3 ± 9.8 dB** | 4kHz: 25.0 ± 8.3 dB  **8kHz: 28.3 ± 7.5 dB**  **12kHz: 48.3 ± 11.6 dB**  **16kHz: 45.0 ± 13.7 dB** | 0.408  0.166  **0.006**  0.074 | +  +  ++  ++ | NA | + |
| **Nan 2022**  **[69]** | ATX | Adult Mice | 1 | Cis + ATX in plx (n=10) | Cis + plx (n=10) | 4mg/kg/d IP, 3d (2 cycles) | 400 µmol/L ATX  (2 cycles) | 1 d prior to cis  IT injection | ABR | 14 d | 4kHz: 77 ± 11 dB SPL*  8kHz: 56 ± 17 dB SPL*  **16kHz: 44 ± 16 dB SPL***  **24kHz: 44 ± 13 dB SPL***  **32kHz: 67 ± 12 dB SPL*** | 4kHz: 81 ± 10 dB SPL*  8kHz: 69 ± 16 dB SPL*  **16kHz: 64 ± 14 dB SPL***  **24kHz: 68 ± 13 dB SPL***  **32kHz: 82 ± 8 dB SPL*** | >0.05  **<0.05**  **<0.01**  **<0.001**  **<0.01** | -  +  ++  ++  + | NA | + |
| **Wen 2021 [70]** | TUDCA | Male Sprague-Dawle rats | 1 | Cis + TUDCA (n=12) | Cis + PBS (n=12) | 12mg/kg IP, single dose | 0.5 mg/mL TUDCA | 1 hour prior to cis  IT injection | ABR | 3 d | Click: 5.83 ± 1.93 dB SPL **8kHz: 25.42 ± 4.86 dB 16 kHz: 21.67 ± 5.52 dB**  **24kHz: 27.08 ± 4.79 dB 32kHz: 40.83 ± 4.07 dB**  40kHz: 37.50 ± 3.40 dB | Click: 8.33 ± 2.33 dB SPL  **8kHz: 32.50 ± 3.96 dB**  **16kHz: 38.33 ± 3.91 dB**  **24kHz: 47.08 ± 3.72 dB**  **32kHz: 61.25 ± 2.82 dB** 40kHz: 56.25 ± 1.86 dB | >0.05  **<0.05**  **<0.05**  **<0.05**  **<0.05**  **<0.05** | -  +  ++  ++  + | + | + |
| *α-lip: α-lipoic acid; GH@PDA@: methacrylate-gelatin microsphere (GH) conjugate polydopamine (PDA) layer, PDA@microcarriers-ALA: polydopamine (PDA) nanoparticles loaded with 𝜶-lipoic acid; ABR: auditory brainstem response; ASSR: Auditory Steady State Response; ATX: astaxanthine; BL: bilateral; CCPA: 2-chloro-N-cyclopentyladenosine; Cis: cisplatin; DPOAE: distortion product otoacoustic emission; EC: epicatechin; FA: folic acid; IT: intratympanic; IP: intraperitoneal; ken: kenpaullone; KR-22332: 3-amino-3-(4-fluoro-phenyl)-1H-quinoline-2,4-dione; NA: not applicable; PBS: phosphate buffered saline; polox: poloxamer; R-PIA: R-phenylisopropyladenosine; RWM: round window membrane; SNR: signal to noise ratio; TUDCA: tauroursodeoxycholic acid; UL: unilateral.*  **results derived from figure using WebPlotDigitizer*  *Design: 1. Ear vs ear; 2. Subject vs subject* | | | | | | | | | | | | | | | | |

Supplementary Table 3. Clinical studies on the otoprotective efficacy and safety of locally administered otoprotectives.

| **Study Design** | | | | | | | | | | | **End point result** | | | | |
| --- | --- | --- | --- | --- | --- | --- | --- | --- | --- | --- | --- | --- | --- | --- | --- |
| **Author, Year** | **Design** | **Diagnosis** | **Intervention Drug** | **Age range**  **(years)** | **Treatment group** | **Comparison group** | **Cisplatin treatment** | **Drug specification** | **Audiometry** | **FU time,**  **Post-TX** | **Hearing function outcomes** | | **P-value** | **Efficacy** | **Safety** |
|  |  |  |  |  |  |  |  |  |  |  | **with intervention drug** | **without intervention drug** |  |  |  |
| **Marshak 2014 [71]** | 1 | Not specified | Dex | 38-80 | Cis + Dex (n=15) | Cis only (n=15) | Cum. dose  517 ± 184 mg,  3-11 cycles | 10 mg/ml Dex solution  IT injection | PTA ,  DPOAE | 1w | PTA (dB HL):  **0.5-3kHz: 18.3 ± 9**  **4-8kHz: 41.1 ± 19.4**  DPOAE (dB SPL):  **1-3 kHz: 8.9 ± 4.7**  **4-8kHz: 6.7 ± 5.9** | PTA (dB HL):  **0.5-3kHz: 19 ± 8.6**  **4-8kHz: 43.8 ± 18.7**  DPOAE (dB SPL):  **1-3kHz: 9.1 ± 4.6**  **4-8kHz: 5.8 ± 4.8** | >0.05  >0.05 | - | NA |
| **Nasr 2018 [72]** | 1 | Not specified | Dex | 40-65 | Cis + Dex (n=20) | Cis only (n=20) | Cum. dose 546.3 ± 111.58 mg  5.5 ± 1 cycles | Dex 4mg/ml  IT injection | PTA | 1 w | PTA (dB HL):  **500Hz: 13.95 ± 3.6**  **1kHz: 16.2 ± 6.1**  **2kHz: 23.25 ± 8.5**  **3kHz: 22.85 ± 8.6**  **4kHz: 40 ± 11.3**  **6kHz: 39.7 ± 11.9**  **8kHz: 43.15 ± 15.6** | PTA (dB HL):  **500Hz: 15.8 ± 4.3**  **1kHz: 15.25 ± 5.7**  **2kHz: 22.58 ± 4.8**  **3kHz: 20.45 ± 7.1**  **4kHz: 40.85 ± 7.7**  **6kHz: 52.6 ± 7.4**  **8kHz: 55.7 ± 13.3** | 0.92428  0.30682  0.38084  0.17377  0.60988  **0.0002**  **0.0094** | +/- | NA |
| **Moreno 2022 [73]** | 1 | Not specified | Dex | 44.2-74.8 | Cis + Dex (n=23) | Cis only (n=23) | Cum. dose 444.87 ± 235.2 mg | 8mg Dex, daily  Microwick device, in RWN | PTA | 3w | 125Hz: 24.7 ± 12.9 dB  250Hz: 15 ± 11.3 dB  **500Hz: 16.2 ± 7 dB**  **1kHz: 15.6 ± 8.1 dB**  **2kHz: 21.2 ± 12.8 dB**  **3kHz: 34.4 ± 20.4 dB**  **4kHz: 45.9 ± 35.6 dB**  **6kHz: 62.9 ± 18.7 dB**  **8kHz: 69.4 ± 19 dB** | 125Hz: 17.4 ± 8.5 dB  250Hz: 11.5 ± 7.7 dB  **500Hz: 11.5 ± 4.6 dB**  **1kHz: 10.6 ± 7.9 dB**  **2kHz: 15.9 ± 7.6 dB**  **3kHz: 27.6 ± 17.1 dB**  **4kHz: 35.6 ± 16.4 dB**  **6kHz: 46.2 ± 20.2 dB**  **8kHz: 61.8 ± 19.8 dB** | 0.06  0.29  0.01  0.02  0.10  0.43  0.11  0.02  0.21 | - | Otorrhea (n=2), permanent perforation (n=8) |
| **Gupta 2023 [74]** | 1 | HNC | Dex | Mean 58.2±6.3 | Cis + Dex (n=100) | Cis only (n=100) | 50 mg/w, 6 w  Cum. dose 300mg  + RT | 0.5-0.7 ml Dex 4mg/ml  Myringotomy | PTA  DPOAE | 12 w | PTA (dB HL):  250Hz: 27.3 ± 2.5  **500Hz: 27.95 ± 3.2**  **1kHz: 19.9 ± 1.6**  **2kHz: 21.35 ± 2.2**  **4kHz: 35.45 ± 4.6**  **6kHz: 42.1 ± 3.2**  **8kHz: 41.65 ± 5.6**  DPOAE (dB SPL):  **998Hz: 7.4 ± 0.64**  **1481Hz: 7.4 ± 0.85**  **2222Hz: 6.9 ± 0.56**  **2963Hz: 8.9 ± 1.13**  **4444Hz: 7.9 ± 0.44**  **5714Hz: 5.3 ± 0.94**  **8000Hz: 5.5 ± 1.36** | PTA (dB HL):  250Hz: 27.15 ± 2.8  **500Hz: 28.9 ± 3.5**  **1kHz: 20.8 ± 2.6**  **2kHz: 20.25 ± 2.7**  **4kHz: 36.45 ± 4.5**  **6kHz: 45.75 ± 3.3**  **8kHz: 47.55 ± 3.5**  DPOAE (dB SPL):  **998Hz: 7.4 ± 1.30**  **1481Hz: 7.4 ± 0.75**  **2222Hz: 7.8 ± 0.99**  **2963Hz: 7.2 ± 0.83**  **4444Hz: 6.1 ± 0.90**  **5714Hz: 3.4 ± 0.74**  **8000Hz: 3.1 ± 0.93** | PTA  >0.05  >0.05  >0.05  >0.05  >0.05  **<0.005**  **<0.05**  DPOAE  >0.05  >0.05  >0.05  >0.05  >0.05  **<0.005**  **<0.05** | +/- | + |
| **Freyer 2025 [75]** | 1 | neuroblastoma (n=9)  Osteosarcoma\| (n=2) | Dex  OTO-104 | 0.5–14 | Cis + Dex (n=11) | Cis only (n=11) | 1-3 cycles of Cis (117 to 393 mg/m2) | IT injection/ tympanostomy 7–64h before Cis 1-3 doses of 0.2 mL of OTO-104 | PTA change from baseline | 1m | After 1st Cis cycle **2kHz: 0.5±17.1 dB HL**  **4kHz: 9.5±22.9 dB HL**  **6kHz: 17.2±25.6 dB HL**  **8kHz: 20.5±23.5 dB HL** After 2nd Cis cycle **2kHz: -2.9±4.9 dB HL**  **4kHz: 17.1±22.5 dB HL**  **6kHz: 31.0±33.1 dB HL**  **8kHz: 48.3±25.6 dB HL** | After 1st Cis cycle  **2kHz: 4.1±25.3 dB HL**  **4kHz: 9.5±28.6 dB HL**  **6kHz: 17.8±34.5 dB HL**  **8kHz: 22.5±32.1 dB HL** After 2nd Cis cycle **2kHz: -2.1±4.9 dB HL**  **4kHz: 14.3±17.7 dB HL**  **6kHz: 33.0±30.5 dB HL**  **8kHz: 45.8±17.4 dB HL** | 0.25  0.90  0.93  0.72  0.80  0.44  0.48  0.70 | - | + |
| **Sarafraz 2018 [76]** | 1 | Solid tumors  (no brain) | NAC | 6-60 | Cis + NAC (n=57) | | Dose NA,  1-3 cycles Cis | 10% NAC solution  IT injection  24 mg/ml Dex  IT injection | PTA | 6 m | 1 Cis injection (NAC)  0.25kHz: 19.7 ± 1.7 dB  **0.5kHz: 20.9 ± 5.3 dB**  **1kHz: 21.7 ± 6.3 dB**  **2kHz: 22.8 ± 6.9 dB**  **4kHz: 23.8 ± 7.9 dB**  **8kHz: 24.7 ± 8.3 dB**  2 Cis injections (NAC)  0.25kHz: 20.4 ± 4 dB  **0.5kHz: 22 ± 6.3 dB**  **1kHz: 23.1 ± 8 dB**  **2kHz: 24.5 ± 8.1 dB**  **4kHz: 24.9 ± 8.5 dB**  **8kHz: 25.7 ± 9.4 dB**  3 Cis injections (NAC)  0.25kHz: 20 ± 2.2 dB  **0.5kHz: 20.8 ± 2 dB**  **1kHz: 20.8 ± 2.1 dB**  **2kHz: 22.2 ± 2.7 dB**  **4kHz: 22.7 ± 2.8 dB**  **8kHz: 23.8 ± 2.8 dB** | 1 Cis injection (Dex)  0.25kHz: 20.9 ± 2.5 dB  **0.5kHz: 22 ± 5.4 dB**  **1kHz: 23 ± 6.3 dB**  **2kHz: 24.2 ± 7.1 dB**  **4kHz: 25.5 ± 8.8 dB**  **8kHz: 27.3 ± 9.6 dB**  2 Cis injections (Dex)  0.25kHz: 21.3 ± 2.7 dB  **0.5kHz: 22.1 ± 4.6 dB**  **1kHz: 23.9 ± 7 dB**  **2kHz: 24.7 ± 7.1 dB**  **4kHz: 27.4 ± 8.1 dB**  **8kHz: 31.7 ± 8.4 dB**  3 Cis injections (Dex)  0.25kHz: 21.4 ± 3.1 dB  **0.5kHz: 22.4 ± 2.9 dB**  **1kHz: 22.3 ± 2.3 dB**  **2kHz: 23.6 ± 2.5 dB**  **4kHz: 25 ± 3.5 dB**  **8kHz: 34.7 ± 5.4 dB** | >0.05  >0.05  >0.05  >0.05  >0.05  >0.05  >0.05  >0.05  >0.05  >0.05  >0.05  >0.05  >0.05  >0.05  >0.05  >0.05  >0.05  **<0.001** | NAC +/- | NAC +  Dex: tinnitus n=20 |
|  |  |  | Dex |  | Cis + Dex (n=57) | |  |  |  |  |  |  |  | Dex – |  |
| **Riga 2013 [77]** | 1 | Solid tumors  (no brain) | NAC | 16-77 | Cis + NAC (n=20) | Cis only (n=20) | 50-100 mg/m^2^, 14-28d.  Cum. dose  120-720 mg/m^2^ | 0.4-0.8 ml 10% NAC solution  IT injection | PTA change from baseline | 1m | 0.25kHz: 1 ± 7.4 dB HL  **0.5kHz: 1.3 ± 7.2 dB HL**  **1kHz: 2.3 ± 6 dB HL**  **2kHz: 0.8 ± 8.3 dB HL**  **4kHz: 0.8 ± 11.6 dB HL**  **8kHz: 0.8 ± 11.3 dB HL** | 0.25kHz: 1.5 ± 8.3 dB HL  **0.5kHz: 0.3 ± 5.6 dB HL**  **1kHz: 4 ± 6.6 dB HL**  **2kHz: 4.3 ± 11.4 dB HL**  **4kHz: 1.3 ± 9.4 dB HL**  **8kHz: 7.8 ± 11.8 dB HL** | 0.8  0.6  0.4  0.2  0.8 **0.005** | +/- | + |
| **Yoo 2014 [78]** | 1 | HNC | NAC | 29-68 | Cis + NAC (n=11) | Cis only (n=11) | 100 mg/m^2^, 2-6 doses + RT | 2% L-NAC solution  IT injection | PTA change from baseline | 1-2m | 0.25kHz: 9.5 dB  **0.5 kHz: 5.5 dB**  **1 kHz: 3.2 dB**  **2 kHz: 4.5 dB**  **3 kHz: 15.0 dB**  **4 kHz: 19.1 dB**  **6 kHz: 30.5 dB**  **8 kHz: 36.4 dB**  9 kHz: 40.0 dB  10 kHz: 37.7 dB  11.2 kHz: 35.0 dB  12.5 kHz: 21.8 dB  14 kHz: 13.2 dB  16 kHz: 9.1 dB  18 kHz: 8.6 dB  20 kHz: 5.5 dB | 0.25kHz: 6.4 dB  **0.5 kHz: 5.5 dB**  **1 kHz: 2.7 dB**  **2 kHz: 3.6 dB**  **3 kHz: 20.5 dB**  **4 kHz: 25dB**  **6 kHz: 32.7 dB**  **8 kHz: 41.4 dB**  9 kHz: 45.5 dB  10 kHz: 44.5 dB  11.2 kHz: 40.0 dB  12.5 kHz: 27.7 dB  14 kHz: 16.8 dB  16 kHz: 10.0 dB  18 kHz: 9.1 dB  20 kHz: 7.7dB | NA | - | + |
| *Cis: cisplatin; Cum.: cumulative; Dex: dexamethasone; DPOAE: distortion product otoacoustic emission; HNC: head and neck cancer; IT: intratympanic; NAC: N-acetylcysteine; PTA: pure tone audiometry; RT: radiotherapy; RWN: round window niche*  *Design: 1. Ear vs ear; 2. Subject vs subject* | | | | | | | | | | | | | | | |

**References:**

1. Daldal, A., O. Odabasi, and B. Serbetcioglu, *The protective effect of intratympanic dexamethasone on cisplatin-induced ototoxicity in guinea pigs.* Otolaryngol Head Neck Surg, 2007. **137**(5): p. 747-52.

2. Hill, G.W., D.K. Morest, and K. Parham, *Cisplatin-Induced Ototoxicity.* Otology & Neurotology, 2008. **29**(7): p. 1005-1011.

3. Murphy, D. and S.J. Daniel, *Intratympanic dexamethasone to prevent cisplatin ototoxicity: a guinea pig model.* Otolaryngol Head Neck Surg, 2011. **145**(3): p. 452-7.

4. Parham, K., *Can intratympanic dexamethasone protect against cisplatin ototoxicity in mice with age-related hearing loss?* Otolaryngol Head Neck Surg, 2011. **145**(4): p. 635-40.

5. Shafik, A.G., et al., *Effect of intratympanic dexamethasone administration on cisplatin-induced ototoxicity in adult guinea pigs.* Auris Nasus Larynx, 2013. **40**(1): p. 51-60.

6. Hughes, A.L., et al., *Dexamethasone otoprotection in a multidose cisplatin ototoxicity mouse model.* Otolaryngol Head Neck Surg, 2014. **150**(1): p. 115-20.

7. Sun, C., et al., *A single dose of dexamethasone encapsulated in polyethylene glycol-coated polylactic acid nanoparticles attenuates cisplatin-induced hearing loss following round window membrane administration.* Int J Nanomedicine, 2015. **10**: p. 3567-79.

8. Fernandez, R., et al., *The Sustained-Exposure Dexamethasone Formulation OTO-104 Offers Effective Protection against Cisplatin-Induced Hearing Loss.* Audiol Neurootol, 2016. **21**(1): p. 22-9.

9. Martin-Saldana, S., et al., *pH-sensitive polymeric nanoparticles with antioxidant and anti-inflammatory properties against cisplatin-induced hearing loss.* J Control Release, 2018. **270**: p. 53-64.

10. Chen, Y., et al., *Dexamethasone-loaded injectable silk-polyethylene glycol hydrogel alleviates cisplatin-induced ototoxicity.* Int J Nanomedicine, 2019. **14**: p. 4211-4227.

11. Mustafa, R.A., et al., *Programmable NIR Responsive Nanocomposite Enables Noninvasive Intratympanic Delivery of Dexamethasone to Reverse Cisplatin Induced Hearing Loss.* Adv Sci (Weinh), 2025. **12**(24): p. e2407067.

12. Dindelegan, M.G., et al., *Hydrogel Matrix Containing Microcarriers for Dexamethasone Delivery to Protect Against Cisplatin-Induced Hearing Loss.* Cureus, 2024. **16**(10): p. e71142.

13. Martin-Saldana, S., et al., *Otoprotective properties of 6alpha-methylprednisolone-loaded nanoparticles against cisplatin: In vitro and in vivo correlation.* Nanomedicine, 2016. **12**(4): p. 965-976.

14. Ramaswamy, B., et al., *Magnetic Nanoparticle Mediated Steroid Delivery Mitigates Cisplatin Induced Hearing Loss.* Front Cell Neurosci, 2017. **11**: p. 268.

15. Pierstorff, E., et al., *Prevention of cisplatin-induced hearing loss by extended release fluticasone propionate intracochlear implants.* Int J Pediatr Otorhinolaryngol, 2019. **121**: p. 157-163.

16. Zhao, W., et al., *The Antioxidant Ergothioneine Alleviates Cisplatin-Induced Hearing Loss Through the Nrf2 Pathway.* Antioxid Redox Signal, 2025. **42**(1-3): p. 97-114.

17. Paksoy, M., et al., *The protective effects of intratympanic dexamethasone and vitamin E on cisplatin-induced ototoxicity are demonstrated in rats.* Med Oncol, 2011. **28**(2): p. 615-21.

18. Ozel, H.E., et al., *Comparison of the protective effects of intratympanic dexamethasone and methylprednisolone against cisplatin-induced ototoxicity.* J Laryngol Otol, 2016. **130**(3): p. 225-34.

19. Martin-Saldana, S., et al., *Polymeric nanoparticles loaded with dexamethasone or alpha-tocopheryl succinate to prevent cisplatin-induced ototoxicity.* Acta Biomater, 2017. **53**: p. 199-210.

20. Simsek, G., et al., *Comparison of the protective efficacy between intratympanic dexamethasone and resveratrol treatments against cisplatin-induced ototoxicity: an experimental study.* Eur Arch Otorhinolaryngol, 2019. **276**(12): p. 3287-3293.

21. Tas, B.M., et al., *Efficacy of 2 Different Intratympanic Steroid Regimen on Prevention of Cisplatin Ototoxicity: An Experimental Study.* Ear Nose Throat J, 2021. **100**(6): p. 417-422.

22. Yi, X., et al., *Magnetic/Acoustic Dual-Controlled Microrobot Overcoming Oto-Biological Barrier for On-Demand Multidrug Delivery against Hearing Loss.* Small, 2024. **20**(44): p. e2401369.

23. Mohan, S., et al., *Targeted amelioration of cisplatin-induced ototoxicity in guinea pigs.* Otolaryngol Head Neck Surg, 2014. **151**(5): p. 836-9.

24. Chen, B.C., et al., *Optimal N-acetylcysteine concentration for intratympanic injection to prevent cisplatin-induced ototoxicity in guinea pigs.* Acta Otolaryngol, 2022. **142**(2): p. 127-131.

25. Choe, W.T., N. Chinosornvatana, and K.W. Chang, *Prevention of cisplatin ototoxicity using transtympanic N-acetylcysteine and lactate.* Otol Neurotol, 2004. **25**(6): p. 910-5.

26. Nader, M.E., Y. Theoret, and I. Saliba, *The role of intratympanic lactate injection in the prevention of cisplatin-induced ototoxicity.* Laryngoscope, 2010. **120**(6): p. 1208-13.

27. Saliba, I., et al., *Are intratympanic injections of N-acetylcysteine and methylprednisolone protective against Cisplatin-induced ototoxicity?* J Otolaryngol Head Neck Surg, 2010. **39**(3): p. 236-43.

28. Li, G., et al., *Round window membrane delivery of L-methionine provides protection from cisplatin ototoxicity without compromising chemotherapeutic efficacy.* Neurotoxicology, 2001. **22**(2): p. 163-76.

29. Korver, K.D., et al., *Round window application of D-methionine provides complete cisplatin otoprotection.* Otolaryngol Head Neck Surg, 2002. **126**(6): p. 683-9.

30. Wimmer, C., et al., *Round window application of D-methionine, sodium thiosulfate, brain-derived neurotrophic factor, and fibroblast growth factor-2 in cisplatin-induced ototoxicity.* Otol Neurotol, 2004. **25**(1): p. 33-40.

31. Ekborn, A., et al., *Intracochlear administration of thiourea protects against cisplatin-induced outer hair cell loss in the guinea pig.* Hear Res, 2003. **181**(1-2): p. 109-15.

32. Wang, J., et al., *Caspase inhibitors, but not c-Jun NH2-terminal kinase inhibitor treatment, prevent cisplatin-induced hearing loss.* Cancer Res, 2004. **64**(24): p. 9217-24.

33. Tanaka, F., C.A. Whitworth, and L.P. Rybak, *Round window pH manipulation alters the ototoxicity of systemic cisplatin.* Hear Res, 2004. **187**(1-2): p. 44-50.

34. More, S.S., et al., *Role of the copper transporter, CTR1, in platinum-induced ototoxicity.* J Neurosci, 2010. **30**(28): p. 9500-9.

35. Saliba, I. and F. El Fata, *Is intratympanic injection of erdosteine protective against cisplatin-induced ototoxicity?* Neurotox Res, 2012. **21**(3): p. 302-8.

36. Spankovich, C., et al., *Assessment of thermal treatment via irrigation of external ear to reduce cisplatin-induced hearing loss.* Hear Res, 2016. **332**: p. 55-60.

37. Stanford, J.K., et al., *Cool OtOprotective Ear Lumen (COOL) Therapy for Cisplatin-induced Hearing Loss.* Otol Neurotol, 2021. **42**(3): p. 466-474.

38. Roldan-Fidalgo, A., et al., *Effect of intratympanic dimethyl sulphoxide (DMSO) in an in vivo model of cisplatin-related ototoxicity.* Eur Arch Otorhinolaryngol, 2014. **271**(12): p. 3121-6.

39. Naples, J.G. and K. Parham, *Cisplatin-Induced Ototoxicity and the Effects of Intratympanic Diltiazem in a Mouse Model.* Otolaryngol Head Neck Surg, 2016. **154**(1): p. 144-9.

40. Naples, J., et al., *Prestin as an Otologic Biomarker of Cisplatin Ototoxicity in a Guinea Pig Model.* Otolaryngol Head Neck Surg, 2018. **158**(3): p. 541-546.

41. Naples, J.G., et al., *Intratympanic Diltiazem-Chitosan Hydrogel as an Otoprotectant Against Cisplatin-Induced Ototoxicity in a Mouse Model.* Otol Neurotol, 2020. **41**(1): p. 115-122.

42. Guven, S.G., et al., *The Effects of Memantine on Cisplatin-Induced Ototoxicity.* Audiol Neurootol, 2025. **30**(3): p. 207-221.

43. Zhou, Z.B., et al., *Bioadhesive drug-loaded microparticles prolong drug retention in the middle ear and ameliorate cisplatin-induced hearing loss.* J Control Release, 2025. **383**: p. 113728.

44. Mukherjea, D., et al., *Transtympanic administration of short interfering (si)RNA for the NOX3 isoform of NADPH oxidase protects against cisplatin-induced hearing loss in the rat.* Antioxid Redox Signal, 2010. **13**(5): p. 589-98.

45. Kaur, T., et al., *Short interfering RNA against STAT1 attenuates cisplatin-induced ototoxicity in the rat by suppressing inflammation.* Cell Death Dis, 2011. **2**(7): p. e180.

46. Ghosh, S., et al., *The Endocannabinoid/Cannabinoid Receptor 2 System Protects Against Cisplatin-Induced Hearing Loss.* Front Cell Neurosci, 2018. **12**: p. 271.

47. Sheehan, K., et al., *Trans-Tympanic Drug Delivery for the Treatment of Ototoxicity.* J Vis Exp, 2018(133).

48. Al Aameri, R.F.H., et al., *Targeting CXCL1 chemokine signaling for treating cisplatin ototoxicity.* Front Immunol, 2023. **14**: p. 1125948.

49. Cooper, L.B., et al., *AAV-mediated delivery of the caspase inhibitor XIAP protects against cisplatin ototoxicity.* Otol Neurotol, 2006. **27**(4): p. 484-90.

50. Chan, D.K., et al., *Protection against cisplatin-induced ototoxicity by adeno-associated virus-mediated delivery of the X-linked inhibitor of apoptosis protein is not dependent on caspase inhibition.* Otol Neurotol, 2007. **28**(3): p. 417-25.

51. Jie, H., et al., *Cochlear protection against cisplatin by viral transfection of X-linked inhibitor of apoptosis protein across round window membrane.* Gene Ther, 2015. **22**(7): p. 546-52.

52. Kuang, R., et al., *Glial cell line-derived neurotrophic factor. Potential for otoprotection.* Ann N Y Acad Sci, 1999. **884**: p. 270-91.

53. Meen, E., B. Blakley, and T. Quddusi, *Does intracochlear brain-derived nerve growth factor improve auditory brainstem click thresholds in sensorineural hearing loss?* J Otolaryngol Head Neck Surg, 2010. **39**(3): p. 232-5.

54. Pisani, A., et al., *Early transtympanic administration of rhBDNF exerts a multifaceted neuroprotective effect against cisplatin-induced hearing loss.* Br J Pharmacol, 2025. **182**(3): p. 546-563.

55. Yurtsever, K.N., et al., *The Protective Effect of Platelet Rich Plasma Against Cisplatin-Induced Ototoxicity.* J Craniofac Surg, 2020. **31**(5): p. e506-e509.

56. Tsai, S.C., et al., *Umbilical Cord Mesenchymal Stromal Cell-Derived Exosomes Rescue the Loss of Outer Hair Cells and Repair Cochlear Damage in Cisplatin-Injected Mice.* Int J Mol Sci, 2021. **22**(13).

57. Whitworth, C.A., et al., *Protection against cisplatin ototoxicity by adenosine agonists.* Biochem Pharmacol, 2004. **67**(9): p. 1801-7.

58. Lee, J.S., et al., *Epicatechin protects the auditory organ by attenuating cisplatin-induced ototoxicity through inhibition of ERK.* Toxicol Lett, 2010. **199**(3): p. 308-16.

59. Celebi, S., et al., *The effect of intratympanic vitamin C administration on cisplatin-induced ototoxicity.* Eur Arch Otorhinolaryngol, 2013. **270**(4): p. 1293-7.

60. Shin, Y.S., et al., *A novel synthetic compound, 3-amino-3-(4-fluoro-phenyl)-1H-quinoline-2,4-dione, inhibits cisplatin-induced hearing loss by the suppression of reactive oxygen species: in vitro and in vivo study.* Neuroscience, 2013. **232**: p. 1-12.

61. Ozkul, Y., et al., *Evaluation of the protective effect of alpha-lipoic acid on cisplatin ototoxicity using distortion-product otoacoustic emission measurements: an experimental animal study.* J Craniofac Surg, 2014. **25**(4): p. 1515-8.

62. Chen, H., et al., *Polydopamine Nanohydrogel Decorated Adhesive and Responsive Hierarchical Microcarriers for Deafness Protection.* Adv Sci (Weinh), 2025. **12**(29): p. e2407637.

63. Demir, M.G., et al., *Effect of Transtympanic Injection of Melatonin on Cisplatin-Induced Ototoxicity.* J Int Adv Otol, 2015. **11**(3): p. 202-6.

64. Roldan-Fidalgo, A., et al., *In vitro and in vivo effects of lutein against cisplatin-induced ototoxicity.* Exp Toxicol Pathol, 2016. **68**(4): p. 197-204.

65. Bekmez Bilmez, Z.E., et al., *Oxytocin as a protective agent in cisplatin-induced ototoxicity.* Cancer Chemother Pharmacol, 2016. **77**(4): p. 875-9.

66. Teitz, T., et al., *CDK2 inhibitors as candidate therapeutics for cisplatin- and noise-induced hearing loss.* J Exp Med, 2018. **215**(4): p. 1187-1203.

67. Hazlitt, R.A., et al., *Development of Second-Generation CDK2 Inhibitors for the Prevention of Cisplatin-Induced Hearing Loss.* J Med Chem, 2018. **61**(17): p. 7700-7709.

68. Tanyeli, T.T., et al., *Effect of Folic Acid on Cisplatin-Induced Ototoxicity: A Functional and Morphological Study.* J Int Adv Otol, 2019. **15**(2): p. 237-246.

69. Nan, B., et al., *Astaxanthine attenuates cisplatin ototoxicity in vitro and protects against cisplatin-induced hearing loss in vivo.* Acta Pharm Sin B, 2022. **12**(1): p. 167-181.

70. Wen, Y., et al., *Tauroursodeoxycholic acid attenuates cisplatin-induced ototoxicity by inhibiting the accumulation and aggregation of unfolded or misfolded proteins in the endoplasmic reticulum.* Toxicology, 2021. **453**: p. 152736.

71. Marshak, T., et al., *Prevention of Cisplatin-Induced Hearing Loss by Intratympanic Dexamethasone: A Randomized Controlled Study.* Otolaryngol Head Neck Surg, 2014. **150**(6): p. 983-90.

72. Nasr, W., et al., *Treatment of cisplatin-induced ototoxicity by intra-tympanic corticosteroid injection.* Indian J Otol, 2018. **24**(1).

73. Moreno, I. and A. Belinchon, *Evaluating the Efficacy of Intratympanic Dexamethasone in Protecting Against Irreversible Hearing Loss in Patients on Cisplatin-Based Cancer Treatment: A Randomized Controlled Phase IIIB Clinical Trial.* Ear Hear, 2022. **43**(2): p. 676-684.

74. Gupta, D., et al., *Intratympanic Dexamethasone Role in Hearing Protection in Cancer Patients.* Cureus, 2023. **15**(8): p. e44299.

75. Freyer, D.R., et al., *Feasibility and Safety of Intratympanic Administration of Sustained-Exposure Dexamethasone Thermosensitive Gel (OTO-104) for Prevention of Cisplatin-Induced Hearing Loss in Children: A Multisite Phase 2 Randomized Clinical Trial.* Pediatr Blood Cancer, 2025. **72**(6): p. e31680.

76. Sarafraz, Z., A. Ahmadi, and A. Daneshi, *Transtympanic Injections of N-acetylcysteine and Dexamethasone for Prevention of Cisplatin-Induced Ototoxicity: Double Blind Randomized Clinical Trial.* Int Tinnitus J, 2018. **22**(1): p. 40-45.

77. Riga, M.G., et al., *Transtympanic injections of N-acetylcysteine for the prevention of cisplatin-induced ototoxicity: a feasible method with promising efficacy.* Am J Clin Oncol, 2013. **36**(1): p. 1-6.

78. Yoo, J., et al., *Cisplatin otoprotection using transtympanic L-N-acetylcysteine: a pilot randomized study in head and neck cancer patients.* Laryngoscope, 2014. **124**(3): p. E87-94.
